# Supplementary material for: Transient Gene Expression is an Effective Experimental Tool for the Research into the Fine Mechanisms of Plant Gene Function: Advantages, Limitations, and Solutions
Source: Plants (Basel). 2020 Sep 11;9(9):1187. doi: 10.3390/plants9091187 (PMC7569937; doi:10.3390/plants9091187)
Supplement: Supplementary file 1 [file plants-09-01187-s001.zip › Table 1S.pdf]

Table 1S. Usage of transient expression technology: plant species, experiment goal and Agrobacterium strains

	Plant species	Experiment goals	Agrobacterium strains	Reference
1	*Agrostis stolonifera*	The physiological role of the gene product in plant growth and development	EHA101	[1]
2	*Arabidopsis thaliana*	Localization of a gene product in a plant cell; The physiological role of the gene product in defense mechanisms when exposed to environmental factors; The physiological role of the gene product in plant growth and development	GV3101, C58C1, EHA C58	[2] [3] [4] [5] [6] [7] [8] [9]
3	*Beta vulgaris*	The physiological role of the gene product in plant growth and development	EHA105	[10]
4	*Brassica oleracea var. italica*	The physiological role of the gene product in defense mechanisms when exposed to environmental factors	GV2260, LBA4404, A208, EHA105	[11]
5	*Capsicum annuum*	Study of protein-protein interaction; Localization of a gene product in a plant cell; The physiological role of the gene product in defense mechanisms when exposed to environmental factors; The physiological role of the gene product in plant growth and development	GV3101, EHA105	[12] [13] [14] [15] [16] [17]
6	*Catharanthus roseus*	The physiological role of the gene product in plant growth and development	GV3101, A.rhizogenesLBA9402/12	[18] [19]
7	*Citrus limon*	The physiological role of the gene product in defense mechanisms when exposed to environmental factors; The physiological role of the gene product in plant growth and development	EHA105	[20] [21]
8	*Citrus paradisi*	The physiological role of the gene product in defense mechanisms when exposed to environmental factors	GV3101, AGL1	[22] [23]
9	*Cucumis sativus*	The physiological role of the gene product in plant growth and development	EHA105	[24]
10	*Daucus carota*	The physiological role of the gene product in plant growth and development	LBA1334	[25]

11	*Eleusine coracana*	The physiological role of the gene product in defense mechanisms when exposed to environmental factors	LBA4404	[26]
12	*Fragaria × ananassa*	The physiological role of the gene product in defense mechanisms when exposed to environmental factors;<br>The physiological role of the gene product in plant growth and development	LBA4404, EHA105	[27] [28] [28] [29]
13	*Fragaria vesca*	The physiological role of the gene product in plant growth and development	GV3101	[30]
14	*Glycine max*	The physiological role of the gene product in plant growth and development	A.rhizogenes K599	[31]
15	*Helianthus annuus*	The physiological role of the gene product in defense mechanisms when exposed to environmental factors	GV3101, LBA4404, EHA105	[32] [33]
16	*Hordeum vulgare*	The physiological role of the gene product in defense mechanisms when exposed to environmental factors	AGL1	[34]
17	*Juglans regia*	The physiological role of the gene product in plant growth and development	EHA105	[35] [36]
18	*Lactuca sativa*	The physiological role of the gene product in plant growth and development	LBA4404, C58	[37] [38]
19	*Lilium longflorum*	The physiological role of the gene product in plant growth and development	LBA4404	[39]
20	*Nicotiana benthamiana*	Study of protein-protein interaction;<br>Localization of a gene product in a plant cell;<br>The physiological role of the gene product in defense mechanisms when exposed to environmental factors;<br>The physiological role of the gene product in plant growth and development	GV2260., C58, ICF320, LBA1334, pGV3850, AGL0, A4, EHA105, GV226, AGL1, C58C1, COR308, LBA4404, ABI, GV2260, ICH011, C58pMP90, GV303, GV3101, C58C1	[40] [41] [42] [43] [44] [45] [46] [47] [48] [49] [50] [51] [52] [53] [54] [55] [56] [57] [58] [59] [60] [61] [62] [63] [64] [65] [66] [67] [68] [69] [70] [71] [72] [73] [74] [75] [76] [77] [78] [79] [80] [81] [82] [83] [84] [85] [86] [87] [88] [89] [90] [91] [92] [93] [94] [95] [96] [97] [98] [99] [100] [101] [102] [103] [104] [105] [106] [107] [108] [109] [110] [111] [111] [112] [113] [114] [115] [116] [117] [118] [119] [120] [121] [122] [123] [124] [125] [126] [127] [128] [129] [130] [131] [12] [132] [133] [134] [135] [38] [136] [137] [138] [139] [140] [141] [142] [143] [144] [145] [146] [147] [148] [149] [150] [151] [152] [153] [154] [155] [156] [157] [158] [159] [160] [161] [162] [163] [164] [165] [166] [167] [168] [169] [170] [171] [172] [173] [174] [175] [176] [177] [178] [179]

				[180] [181] [182] [183] [184] [185] [186] [187] [188] [189] [190] [191] [192] [193] [194] [195] [196] [197] [198] [199] [200] [201] [202] [32] [203] [204] [205] [206] [207] [208] [209] [210] [211] [212] [213] [214] [215] [216] [217] [218] [219] [220] [221] [222] [223] [224] [225] [226] [227] [228] [229] [230] [231] [232] [233] [234] [235] [236] [237] [238] [239] [240] [241] [242] [243] [244] [245] [246] [247] [248] [249] [250] [251] [252] [253] [254] [255] [256] [257] [258] [259] [260] [261] [262] [263] [264] [265] [266] [267] [268] [269] [270] [271] [272] [273] [274] [275] [276] [277] [278] [279] [280] [281] [282] [283] [284] [285] [286] [287] [288] [289] [290] [291] [292] [293] [294] [295] [296] [297] [298] [299] [300] [301] [302] [303] [304] [305] [306] [307] [308] [309] [310] [311] [312] [313] [314] [315] [316] [317] [318] [319] [320] [321] [322] [323] [324] [325] [326] [327] [328] [329] [330] [331] [332] [16] [333] [334] [335] [336] [337] [338] [339] [340] [341] [342] [343] [344] [345] [346] [347] [348] [349] [350] [351] [352] [353] [354] [355] [356] [357] [358] [359] [360] [361] [362] [363] [364] [365] [366] [367] [368] [369] [370] [371] [372] [373] [374] [375]
21	*Nicotiana clevelandii*	Localization of a gene product in a plant cell	GV3101	[359]
22	*Nicotiana excelsior*	The physiological role of the gene product in plant growth and development	GV3101	[376]
23	*Nicotiana tabacum*	Localization of a gene product in a plant cell;<br>The physiological role of the gene product in defense mechanisms when exposed to environmental factors;<br>The physiological role of the gene product in plant growth and development	C58C1, LBA4404, GV3101, EHA105, C58C1-313	[377] [306] [77] [378] [379] [380] [381] [382] [383] [384] [385] [386] [239] [266] [387] [272] [388] [389] [390] [391] [392] [393] [394] [5] [10] [395] [396] [397] [398] [399] [359] [400] [401] [402] [403] [404] [405] [406]
24	*Ocimum kilimandscharicum*	The physiological role of the gene product in plant growth and development	GV3101	[407]
25	*Oryza sativa*	The physiological role of the gene product in defense mechanisms when exposed to environmental factors;<br>The physiological role of the gene product in plant growth and development	LBA4404	[408]
26	*Papaver somniferum*	The physiological role of the gene product in	C58	[409]

		plant growth and development		
27	*Petunia hybrida*	The physiological role of the gene product in defense mechanisms when exposed to environmental factors	LBA4404	[379]
28	*Populus tremula* × *alba*	The physiological role of the gene product in plant growth and development	GV3101	[179]
29	*Prunus persica*	The physiological role of the gene product in plant growth and development	GV3101	[410]
30	*Robinia pseudoacacia*	The physiological role of the gene product in defense mechanisms when exposed to environmental factors	AGL1	[411]
31	*Sesbania grandiflora*	The physiological role of the gene product in defense mechanisms when exposed to environmental factors	C58C1	[412]
32	*Solanum capsicum*	The physiological role of the gene product in plant growth and development	GV3101	[413]
33	*Solanum chilense*	The physiological role of the gene product in plant growth and development	LBA4404	[396]
34	*Solanum lycopersicum*	Localization of a gene product in a plant cell;<br>The physiological role of the gene product in defense mechanisms when exposed to environmental factors;<br>The physiological role of the gene product in plant growth and development	GV3101, LBA4404, EHA105	[414] [415] [266] [272] [298] [416] [396] [5]
35	*Solanum peruvianum*	The physiological role of the gene product in plant growth and development	LBA4404	[396]
36	*Solanum torvum*	The physiological role of the gene product in defense mechanisms when exposed to environmental factors	GV3101	[315]
37	*Tamarix hispida*	The physiological role of the gene product in plant growth and development	GV3101	[417] [418] [419]
38	*Theobroma cacao*	The physiological role of the gene product in plant growth and development	AGL1	[420] [421]
	Tropaeolum majus	The physiological role of the gene product in plant growth and development	GV3101	[252]

40	*Urtica dioica*	The physiological role of the gene product in plant growth and development		[422]
41	*Vitis pseudoreticulat*	The physiological role of the gene product in defense mechanisms when exposed to environmental factors	GV3101	[423]
42	*Vitis pseudoreticulata*	The physiological role of the gene product in plant growth and development	GV3101	[424]
43	*Vitis vinifera*	The physiological role of the gene product in defense mechanisms when exposed to environmental factors;<br>The physiological role of the gene product in plant growth and development	GV3101, LBA4404, EHA105	[425] [423] [426] [427] [428] [429]

Rtferences to Table 1S

1. Fu, D.; Huang, B.; Xiao, Y.; Muthukrishnan, S.; Liang, G.H. Overexpression of barley hva1 gene in creeping bentgrass for improving drought tolerance. *Plant Cell Reports* **2007**, *26*, 467-477, doi:10.1007/s00299-006-0258-7.
2. Wang, Y.-C.; Yu, M.; Shih, P.-Y.; Wu, H.-Y.; Lai, E.-M. Stable pH Suppresses Defense Signaling and is the Key to Enhance Agrobacterium-Mediated Transient Expression in Arabidopsis Seedlings. *Scientific Reports* **2018**, *8*, 17071, doi:10.1038/s41598-018-34949-9.
3. Wang, F.-P.; Wang, X.-F.; Zhang, J.; Ma, F.; Hao, Y.-J. MdMYB58 Modulates Fe Homeostasis by Directly Binding to the MdMATE43 Promoter in Plants. *Plant and Cell Physiology* **2018**, *59*, 2476-2489, doi:10.1093/pcp/pcy168.
4. Yoshimura, K.; Nakane, T.; Kume, S.; Shiomi, Y.; Maruta, T.; Ishikawa, T.; Shigeoka, S. Transient expression analysis revealed the importance of VTC2 expression level in light/dark regulation of ascorbate biosynthesis in Arabidopsis. *Bioscience, biotechnology, and biochemistry* **2014**, *78*, 60-66.
5. Kawazu, K.; Wasano, N.; Konno, K.; Ohashi, Y.; Mochizuki, A.; Mitsuhara, I. Evaluation of anti-herbivory genes using an Agrobacterium-mediated transient expression system. *Plant Biotechnology* **2012**, *29*, 495-499, doi:https://doi.org/10.5511/plantbiotechnology.12.0711a.
6. Tsuda, K.; Qi, Y.; Nguyen, L.V.; Bethke, G.; Tsuda, Y.; Glazebrook, J.; Katagiri, F. An efficient Agrobacterium-mediated transient transformation of Arabidopsis. *The Plant Journal* **2012**, *69*, 713-719, doi:10.1111/j.1365-313X.2011.04819.x.
7. Marion, J.; Bach, L.; Bellec, Y.; Meyer, C.; Gissot, L.; Faure, J.-D. Systematic analysis of protein subcellular localization and interaction using high-throughput transient transformation of Arabidopsis seedlings. *The Plant Journal* **2008**, *56*, 169-179, doi:10.1111/j.1365-313X.2008.03596.x.
8. Mudgett, M.B.; Chesnokova, O.; Dahlbeck, D.; Clark, E.T.; Rossier, O.; Bonas, U.; Staskawicz, B.J. Molecular signals required for type III secretion and translocation of the Xanthomonas campestris AvrBs2 protein to pepper plants. *Proceedings of the National Academy of Sciences* **2000**, *97*, 13324-13329, doi:10.1073/pnas.230450797.
9. Choi, H.W.; Lee, D.H.; Hwang, B.K. The Pepper Calmodulin Gene CaCaM1 Is Involved in Reactive Oxygen Species and Nitric Oxide Generation Required for Cell Death and the Defense Response. *Molecular Plant-Microbe Interactions®* **2009**, *22*, 1389-1400, doi:10.1094/mpmi-22-11-1389.
10. Lacroix, B.; Citovsky, V. Extracellular VirB5 enhances T-DNA transfer from Agrobacterium to the host plant. *PLoS One* **2011**, *6*, e25578, doi:10.1371/journal.pone.0025578.
11. Wagner, B.; Fuchs, H.; Adhami, F.; Ma, Y.; Scheiner, O.; Breiteneder, H. Plant virus expression systems for transient production of recombinant allergens in Nicotiana benthamiana. *Methods* **2004**, *32*, 227-234, doi:10.1016/j.ymeth.2003.08.005.
12. Noman, A.; Liu, Z.; Yang, S.; Shen, L.; Hussain, A.; Ashraf, M.F.; Khan, M.I.; He, S. Expression and functional evaluation of CaZNF830 during pepper response to Ralstonia solanacearum or high temperature and humidity. *Microbial Pathogenesis* **2018**, *118*, 336-346, doi:https://doi.org/10.1016/j.micpath.2018.03.044.
13. Kim, N.H.; Hwang, B.K. Pepper pathogenesis-related protein 4c is a plasma membrane-localized cysteine protease inhibitor that is required for plant cell death and defense signaling. *The Plant Journal* **2015**, *81*, 81-94, doi:10.1111/tpj.12709.
14. Choi, H.W.; Kim, D.S.; Kim, N.H.; Jung, H.W.; Ham, J.H.; Hwang, B.K. Xanthomonas Filamentous Hemagglutinin-Like Protein Fha1 Interacts with Pepper Hypersensitive-Induced Reaction Protein CaHIR1 and Functions as a Virulence Factor in Host Plants. *Molecular Plant-Microbe Interactions®* **2013**, *26*, 1441-1454, doi:10.1094/mpmi-07-13-0204-r.
15. Hwang, I.S.; Choi, D.S.; Kim, N.H.; Kim, D.S.; Hwang, B.K. The pepper cysteine/histidine-rich DC1 domain protein CaDC1 binds both RNA and DNA and is required for plant cell death and defense response. *New Phytologist* **2014**, *201*, 518-530, doi:10.1111/nph.12521.
16. Kim, D.S.; Jeun, Y.; Hwang, B.K. The pepper patatin-like phospholipase CaPLP1 functions in plant cell death and defense signaling. *Plant Mol Biol* **2014**, *84*, 329-344, doi:10.1007/s11103-013-0137-x.

17. Lee, B.-J.; Kwon, S.J.; Kim, S.-K.; Kim, K.-J.; Park, C.-J.; Kim, Y.-J.; Park, O.K.; Paek, K.-H. Functional study of hot pepper 26S proteasome subunit RPN7 induced by Tobacco mosaic virus from nuclear proteome analysis. *Biochemical and Biophysical Research Communications* **2006**, *351*, 405-411, doi:https://doi.org/10.1016/j.bbrc.2006.10.071.
18. Han, J.; Liu, H.-t.; Wang, S.-c.; Wang, C.-r.; Miao, G.-p. A class I TGA transcription factor from Tripterygium wilfordii Hook.f. modulates the biosynthesis of secondary metabolites in both native and heterologous hosts. *Plant Science* **2020**, *290*, 110293, doi:https://doi.org/10.1016/j.plantsci.2019.110293.
19. Mertens, J.; Van Moerkercke, A.; Vanden Bossche, R.; Pollier, J.; Goossens, A. Clade IVa Basic Helix–Loop–Helix Transcription Factors Form Part of a Conserved Jasmonate Signaling Circuit for the Regulation of Bioactive Plant Terpenoid Biosynthesis. *Plant and Cell Physiology* **2016**, *57*, 2564-2575, doi:10.1093/pcp/pcw168.
20. Sendín, L.; Filippone, M.; Orce, I.; Rigano, L.; Enrique, R.; Peña, L.; Vojnov, A.; Marano, M.; Castagnaro, A. Transient expression of pepper Bs2 gene in Citrus limon as an approach to evaluate its utility for management of citrus canker disease. *Plant pathology* **2012**, *61*, 648-657.
21. Enrique, R.; Siciliano, F.; Favaro, M.A.; Gerhardt, N.; Roeschlin, R.; Rigano, L.; Sendin, L.; Castagnaro, A.; Vojnov, A.; Marano, M.R. Novel demonstration of RNAi in citrus reveals importance of citrus callose synthase in defence against Xanthomonas citri subsp. citri. *Plant Biotechnology Journal* **2011**, *9*, 394-407, doi:10.1111/j.1467-7652.2010.00555.x.
22. Shi, Q.; Febres, V.J.; Jones, J.B.; Moore, G.A. A survey of FLS2 genes from multiple citrus species identifies candidates for enhancing disease resistance to Xanthomonas citri ssp. citri. *Hortic Res* **2016**, *3*, 16022, doi:10.1038/hortres.2016.22.
23. Figueiredo, J.F.L.; Römer, P.; Lahaye, T.; Graham, J.H.; White, F.F.; Jones, J.B. Agrobacterium-mediated transient expression in citrus leaves: a rapid tool for gene expression and functional gene assay. *Plant Cell Reports* **2011**, *30*, 1339-1345, doi:10.1007/s00299-011-1045-7.
24. Pimenta Lange, M.J.; Lange, T. Ovary-derived precursor gibberellin A9 is essential for female flower development in cucumber. *Development* **2016**, *143*, 4425-4429, doi:10.1242/dev.135947.
25. Baranski, R.; Klocke, E.; Ryschka, U. Monitoring the expression of green fluorescent protein in carrot. *Acta Physiologiae Plantarum* **2007**, *29*, 239-246, doi:10.1007/s11738-007-0030-9.
26. Ignacimuthu, S.; Ceasar, S.A. Development of transgenic finger millet (Eleusine coracana (L.) Gaertn.) resistant to leaf blast disease. *J Biosci* **2012**, *37*, 135-147, doi:10.1007/s12038-011-9178-y.
27. Zhao, Y.; Mao, W.; Chen, Y.; Wang, W.; Dai, Z.; Dou, Z.; Zhang, K.; Wei, L.; Li, T.; Zeng, B., et al. Optimization and standardization of transient expression assays for gene functional analyses in strawberry fruits. *Hortic Res* **2019**, *6*, 53, doi:10.1038/s41438-019-0135-5.
28. Cheng, J.; Wen, S.; Xiao, S.; Lu, B.; Ma, M.; Bie, Z. Overexpression of the tonoplast sugar transporter CmTST2 in melon fruit increases sugar accumulation. *J Exp Bot* **2018**, *69*, 511-523, doi:10.1093/jxb/erx440.
29. Aragüez, I.; Osorio, S.; Hoffmann, T.; Rambla, J.L.; Medina-Escobar, N.; Granell, A.; Botella, M.Á.; Schwab, W.; Valpuesta, V. Eugenol Production in Achenes and Receptacles of Strawberry Fruits Is Catalyzed by Synthases Exhibiting Distinct Kinetics. *Plant Physiology* **2013**, *163*, 946-958, doi:10.1104/pp.113.224352.
30. Xie, Y.-G.; Ma, Y.-Y.; Bi, P.-P.; Wei, W.; Liu, J.; Hu, Y.; Gou, Y.-J.; Zhu, D.; Wen, Y.-Q.; Feng, J.-Y. Transcription factor FvTCP9 promotes strawberry fruit ripening by regulating the biosynthesis of abscisic acid and anthocyanins. *Plant Physiology and Biochemistry* **2020**, *146*, 374-383, doi:https://doi.org/10.1016/j.plaphy.2019.11.004.
31. Huang, J.; Gu, L.; Zhang, Y.; Yan, T.; Kong, G.; Kong, L.; Guo, B.; Qiu, M.; Wang, Y.; Jing, M., et al. An oomycete plant pathogen reprograms host pre-mRNA splicing to subvert immunity. *Nature Communications* **2017**, *8*, 2051, doi:10.1038/s41467-017-02233-5.
32. Gascuel, Q.; Buendia, L.; Pecrix, Y.; Blanchet, N.; Munos, S.; Vear, F.; Godiard, L. RXLR and CRN Effectors from the Sunflower Downy Mildew Pathogen Plasmopara halstedii Induce Hypersensitive-Like Responses in Resistant Sunflower Lines. *Front Plant Sci* **2016**, *7*, 1887, doi:10.3389/fpls.2016.01887.

33. Jung, S.K.; Lindenmuth, B.E.; McDonald, K.A.; Hwang, H.; Bui, M.Q.; Falk, B.W.; Uratsu, S.L.; Phu, M.L.; Dandekar, A.M. Agrobacterium tumefaciens mediated transient expression of plant cell wall-degrading enzymes in detached sunflower leaves. *Biotechnol Prog* **2014**, *30*, 905-915, doi:10.1002/btpr.1888.
34. Patel, M.; Johnson, J.S.; Brettell, R.I.S.; Jacobsen, J.; Xue, G.-P. Transgenic barley expressing a fungal xylanase gene in the endosperm of the developing grains. *Molecular Breeding* **2000**, *6*, 113-124, doi:10.1023/A:1009640427515.
35. Yang, G.; Gao, X.; Ma, K.; Li, D.; Jia, C.; Zhai, M.; Xu, Z. The walnut transcription factor JrGRAS2 contributes to high temperature stress tolerance involving in Dof transcriptional regulation and HSP protein expression. *BMC Plant Biology* **2018**, *18*, 367, doi:10.1186/s12870-018-1568-y.
36. Yang, G.; Zhang, W.; Liu, Z.; Yi-Maer, A.Y.; Zhai, M.; Xu, Z. Both JrWRKY2 and JrWRKY7 of Juglans regia mediate responses to abiotic stresses and abscisic acid through formation of homodimers and interaction. *Plant Biol (Stuttg)* **2017**, *19*, 268-278, doi:10.1111/plb.12524.
37. Simmons, C.W.; VanderGheynst, J.S. Transient co-expression of post-transcriptional gene silencing suppressors and beta-glucuronidase in harvested lettuce leaf tissue does not improve recombinant protein accumulation in planta. *Biotechnol Lett* **2007**, *29*, 641-645, doi:10.1007/s10529-006-9279-0.
38. Rosenthal, S.H.; Diamos, A.G.; Mason, H.S. An intronless form of the tobacco extensin gene terminator strongly enhances transient gene expression in plant leaves. *Plant Molecular Biology* **2018**, *96*, 429-443, doi:10.1007/s11103-018-0708-y.
39. Kim, S.S.; Shin, D.I.; Park, H.S. Transient beta-glucuronidase expression in lily (Lilium longflorum L.) pollen via wounding-assisted Agrobacterium-mediated transformation. *Biotechnol Lett* **2007**, *29*, 965-969, doi:10.1007/s10529-007-9326-5.
40. Qi, X.; Tang, X.; Liu, W.; Fu, X.; Luo, H.; Ghimire, S.; Zhang, N.; Si, H. A potato RING-finger protein gene StRFP2 is involved in drought tolerance. *Plant Physiol Biochem* **2020**, *146*, 438-446, doi:10.1016/j.plaphy.2019.11.042.
41. Xu, Q.; Tang, C.; Wang, X.; Sun, S.; Zhao, J.; Kang, Z.; Wang, X. An effector protein of the wheat stripe rust fungus targets chloroplasts and suppresses chloroplast function. *Nat Commun* **2019**, *10*, 5571, doi:10.1038/s41467-019-13487-6.
42. Situ, J.; Jiang, L.; Fan, X.; Yang, W.; Li, W.; Xi, P.; Deng, Y.; Kong, G.; Jiang, Z. An RXLR effector PlAvh142 from Peronophythora litchii triggers plant cell death and contributes to virulence. *Mol Plant Pathol* **2020**, *21*, 415-428, doi:10.1111/mpp.12905.
43. Grijalva-Manay, R.; Dorca-Fornell, C.; Enriquez-Villacreses, W.; Mino-Castro, G.; Oliva, R.; Ochoa, V.; Proano-Tuma, K.; Armijos-Jaramillo, V. DnaJ molecules as potential effectors in Meloidogyne arenaria. An unexplored group of proteins in plant parasitic nematodes. *Commun Integr Biol* **2019**, *12*, 151-161, doi:10.1080/19420889.2019.1676138.
44. Anwar, M.; Yu, W.; Yao, H.; Zhou, P.; Allan, A.C.; Zeng, L. NtMYB3, an R2R3-MYB from Narcissus, Regulates Flavonoid Biosynthesis. In *International journal of molecular sciences*, 2019; Vol. 20.
45. Xu, J.; Lee, Y.J.; Liu, B. Establishment of a mitotic model system by transient expression of the D-type cyclin in differentiated leaf cells of tobacco (Nicotiana benthamiana). *New Phytol* **2020**, *226*, 1213-1220, doi:10.1111/nph.16309.
46. Ding, L.N.; Li, M.; Guo, X.J.; Tang, M.Q.; Cao, J.; Wang, Z.; Liu, R.; Zhu, K.M.; Guo, L.; Liu, S.Y., et al. Arabidopsis GDSL1 overexpression enhances rapeseed Sclerotinia sclerotiorum resistance and the functional identification of its homolog in Brassica napus. *Plant Biotechnol J* **2020**, *18*, 1255-1270, doi:10.1111/pbi.13289.
47. Miao, X.; Zhang, L.; Hu, X.; Nan, S.; Chen, X.; Fu, H. Cloning and functional analysis of the FAD2 gene family from desert shrub Artemisia sphaerocephala. *BMC Plant Biol* **2019**, *19*, 481, doi:10.1186/s12870-019-2083-5.
48. Patton, M.F.; Bak, A.; Sayre, J.M.; Heck, M.L.; Casteel, C.L. A polerovirus, Potato leafroll virus, alters plant-vector interactions using three viral proteins. *Plant Cell Environ* **2020**, *43*, 387-399, doi:10.1111/pce.13684.
49. Lei, X.; Lan, X.; Ye, W.; Liu, Y.; Song, S.; Lu, J. Plasmopara viticola effector PvRXLR159 suppresses immune responses in Nicotiana benthamiana. *Plant Signal Behav* **2019**, *14*, 1682220, doi:10.1080/15592324.2019.1682220.

50. Wang, X.; Zeng, L.; Liao, Y.; Li, J.; Tang, J.; Yang, Z. Formation of alpha-Farnesene in Tea (Camellia sinensis) Leaves Induced by Herbivore-Derived Wounding and Its Effect on Neighboring Tea Plants. *Int J Mol Sci* **2019**, *20*, doi:10.3390/ijms20174151.
51. Chen, J.; Li, H.; Yang, K.; Wang, Y.; Yang, L.; Hu, L.; Liu, R.; Shi, Z. Melatonin facilitates lateral root development by coordinating PAO-derived hydrogen peroxide and Rboh-derived superoxide radical. *Free Radic Biol Med* **2019**, *143*, 534-544, doi:10.1016/j.freeradbiomed.2019.09.011.
52. Qi, X.; Qu, Y.; Gao, R.; Jiang, J.; Fang, W.; Guan, Z.; Zhang, F.; Zhao, S.; Chen, S.; Chen, F., et al. The Heterologous Expression of a Chrysanthemum nankingense TCP Transcription Factor Blocks Cell Division in Yeast and Arabidopsis thaliana. *Int J Mol Sci* **2019**, *20*, doi:10.3390/ijms20194848.
53. Zhang, D.; Yang, H.; Wang, X.; Qiu, Y.; Tian, L.; Qi, X.; Qu, L.Q. Cytochrome P450 family member CYP96B5 hydroxylates alkanes to primary alcohols and is involved in rice leaf cuticular wax synthesis. *New Phytol* **2020**, *225*, 2094-2107, doi:10.1111/nph.16267.
54. Zhang, J.; Li, L.; Huang, L.; Zhang, M.; Chen, Z.; Zheng, Q.; Zhao, H.; Chen, X.; Jiang, M.; Tan, M. Maize NAC-domain retained splice variants act as dominant negatives to interfere with the full-length NAC counterparts. *Plant Sci* **2019**, *289*, 110256, doi:10.1016/j.plantsci.2019.110256.
55. Jimenez-Guerrero, I.; Perez-Montano, F.; Da Silva, G.M.; Wagner, N.; Shkedy, D.; Zhao, M.; Pizarro, L.; Bar, M.; Walcott, R.; Sessa, G., et al. Show me your secret(ed) weapons: a multifaceted approach reveals a wide arsenal of type III-secreted effectors in the cucurbit pathogenic bacterium Acidovorax citrulli and novel effectors in the Acidovorax genus. *Mol Plant Pathol* **2020**, *21*, 17-37, doi:10.1111/mpp.12877.
56. Qi, M.; Mei, Y.; Grayczyk, J.P.; Darben, L.M.; Rieker, M.E.G.; Seitz, J.M.; Voegele, R.T.; Whitham, S.A.; Link, T.I. Candidate Effectors From Uromyces appendiculatus, the Causal Agent of Rust on Common Bean, Can Be Discriminated Based on Suppression of Immune Responses. *Front Plant Sci* **2019**, *10*, 1182, doi:10.3389/fpls.2019.01182.
57. Liu, M.; Ma, Y.; Du, Q.; Hou, X.; Wang, M.; Lu, S. Functional Analysis of Polyprenyl Diphosphate Synthase Genes Involved in Plastoquinone and Ubiquinone Biosynthesis in Salvia miltiorrhiza. *Front Plant Sci* **2019**, *10*, 893, doi:10.3389/fpls.2019.00893.
58. Zhao, J.; Li, L.; Liu, Q.; Liu, P.; Li, S.; Yang, D.; Chen, Y.; Pagnotta, S.; Favery, B.; Abad, P., et al. A MIF-like effector suppresses plant immunity and facilitates nematode parasitism by interacting with plant annexins. *J Exp Bot* **2019**, *70*, 5943-5958, doi:10.1093/jxb/erz348.
59. Kim, H.S.; Bian, X.; Lee, C.J.; Kim, S.E.; Park, S.C.; Xie, Y.; Guo, X.; Kwak, S.S. IbMPK3/IbMPK6-mediated IbSPF1 phosphorylation promotes tolerance to bacterial pathogen in sweetpotato. *Plant Cell Rep* **2019**, *38*, 1403-1415, doi:10.1007/s00299-019-02451-9.
60. van Eerde, A.; Varnai, A.; Jameson, J.K.; Paruch, L.; Moen, A.; Anonsen, J.H.; Chylenski, P.; Steen, H.S.; Heldal, I.; Bock, R., et al. In-depth characterization of Trichoderma reesei cellobiohydrolase TrCel7A produced in Nicotiana benthamiana reveals limitations of cellulase production in plants by host-specific post-translational modifications. *Plant Biotechnol J* **2020**, *18*, 631-643, doi:10.1111/pbi.13227.
61. Kovalskaya, N.Y.; Herndon, E.E.; Foster-Frey, J.A.; Donovan, D.M.; Hammond, R.W. Antimicrobial activity of bacteriophage derived triple fusion protein against Staphylococcus aureus. *AIMS Microbiol* **2019**, *5*, 158-175, doi:10.3934/microbiol.2019.2.158.
62. Price, A.M.; Doner, N.M.; Gidda, S.K.; Jambunathan, S.; James, C.N.; Schami, A.; Yurchenko, O.; Mullen, R.T.; Dyer, J.M.; Puri, V., et al. Mouse Fat-Specific Protein 27 (FSP27) expressed in plant cells localizes to lipid droplets and promotes lipid droplet accumulation and fusion. *Biochimie* **2020**, *169*, 41-53, doi:10.1016/j.biochi.2019.08.002.
63. Guo, X.; Zhong, D.; Xie, W.; He, Y.; Zheng, Y.; Lin, Y.; Chen, Z.; Han, Y.; Tian, D.; Liu, W., et al. Functional Identification of Novel Cell Death-inducing Effector Proteins from Magnaporthe oryzae. *Rice (N Y)* **2019**, *12*, 59, doi:10.1186/s12284-019-0312-z.
64. Zhu, Y.X.; Yang, L.; Liu, N.; Yang, J.; Zhou, X.K.; Xia, Y.C.; He, Y.; He, Y.Q.; Gong, H.J.; Ma, D.F., et al. Genome-wide identification, structure characterization, and expression pattern profiling of aquaporin gene family in cucumber. *BMC Plant Biol* **2019**, *19*, 345, doi:10.1186/s12870-019-1953-1.
65. Wang, A.; Pan, L.; Niu, X.; Shu, X.; Yi, X.; Yamamoto, N.; Li, S.; Deng, Q.; Zhu, J.; Liang, Y., et al. Comparative secretome analysis of different smut fungi and identification of plant cell death-

inducing secreted proteins from Tilletia horrida. *BMC Plant Biol* **2019**, *19*, 360, doi:10.1186/s12870-019-1924-6.
66. Khaksar, G.; Sangchay, W.; Pinsorn, P.; Sangpong, L.; Sirikantaramas, S. Genome-wide analysis of the Dof gene family in durian reveals fruit ripening-associated and cultivar-dependent Dof transcription factors. *Sci Rep* **2019**, *9*, 12109, doi:10.1038/s41598-019-48601-7.
67. Islam, M.R.; Son, N.; Lee, J.; Lee, D.W.; Sohn, E.J.; Hwang, I. Production of bacteriophage-encoded endolysin, LysP11, in Nicotiana benthamiana and its activity as a potent antimicrobial agent against Erysipelothrix rhusiopathiae. *Plant Cell Rep* **2019**, *38*, 1485-1499, doi:10.1007/s00299-019-02459-1.
68. Berthold, F.; Roujol, D.; Hemmer, C.; Jamet, E.; Ritzenthaler, C.; Hoffmann, L.; Schmitt-Keichinger, C. Inside or outside? A new collection of Gateway vectors allowing plant protein subcellular localization or over-expression. *Plasmid* **2019**, *105*, 102436, doi:10.1016/j.plasmid.2019.102436.
69. Zhou, D.; Shen, Y.; Zhou, P.; Fatima, M.; Lin, J.; Yue, J.; Zhang, X.; Chen, L.Y.; Ming, R. Papaya CpbHLH1/2 regulate carotenoid biosynthesis-related genes during papaya fruit ripening. *Hortic Res* **2019**, *6*, 80, doi:10.1038/s41438-019-0162-2.
70. Wang, H.; Liu, Y.; Peng, Z.; Li, J.; Huang, W.; Liu, Y.; Wang, X.; Xie, S.; Sun, L.; Han, E., et al. Ectopic Expression of Poplar ABC Transporter PtoABCG36 Confers Cd Tolerance in Arabidopsis thaliana. *Int J Mol Sci* **2019**, *20*, doi:10.3390/ijms20133293.
71. Diao, P.; Zhang, Q.; Sun, H.; Ma, W.; Cao, A.; Yu, R.; Wang, J.; Niu, Y.; Wuriyanghan, H. miR403a and SA Are Involved in NbAGO2 Mediated Antiviral Defenses Against TMV Infection in Nicotiana benthamiana. *Genes (Basel)* **2019**, *10*, doi:10.3390/genes10070526.
72. Yin, X.; Shang, B.; Dou, M.; Liu, R.; Chen, T.; Xiang, G.; Li, Y.; Liu, G.; Xu, Y. The Nuclear-Localized RxLR Effector PvAvh74 From Plasmopara viticola Induces Cell Death and Immunity Responses in Nicotiana benthamiana. *Front Microbiol* **2019**, *10*, 1531, doi:10.3389/fmicb.2019.01531.
73. Zhou, X.; Sun, K.; Zhou, X.; Jackson, A.O.; Li, Z. The Matrix Protein of a Plant Rhabdovirus Mediates Superinfection Exclusion by Inhibiting Viral Transcription. *J Virol* **2019**, *93*, doi:10.1128/JVI.00680-19.
74. Kopertekh, L.; Schiemann, J. Enhanced foreign protein accumulation in Nicotiana benthamiana leaves co-infiltrated with a TMV vector and plant cell cycle regulator genes. *Transgenic Res* **2019**, *28*, 411-417, doi:10.1007/s11248-019-00128-3.
75. Fang, A.; Gao, H.; Zhang, N.; Zheng, X.; Qiu, S.; Li, Y.; Zhou, S.; Cui, F.; Sun, W. A Novel Effector Gene SCRE2 Contributes to Full Virulence of Ustilaginoidea virens to Rice. *Front Microbiol* **2019**, *10*, 845, doi:10.3389/fmicb.2019.00845.
76. Bruns, A.N.; Li, S.; Mohannath, G.; Bisaro, D.M. Phosphorylation of Arabidopsis eIF4E and eIFiso4E by SnRK1 inhibits translation. *FEBS J* **2019**, *286*, 3778-3796, doi:10.1111/febs.14935.
77. Gengenbach, B.B.; Keil, L.L.; Opdensteinen, P.; Muschen, C.R.; Melmer, G.; Lentzen, H.; Buhrmann, J.; Buyel, J.F. Comparison of microbial and transient expression (tobacco plants and plant-cell packs) for the production and purification of the anticancer mistletoe lectin viscumin. *Biotechnol Bioeng* **2019**, *116*, 2236-2249, doi:10.1002/bit.27076.
78. Li, C.; Zhao, M.; Ma, X.; Wen, Z.; Ying, P.; Peng, M.; Ning, X.; Xia, R.; Wu, H.; Li, J. The HD-Zip transcription factor LcHB2 regulates litchi fruit abscission through the activation of two cellulase genes. *J Exp Bot* **2019**, *70*, 5189-5203, doi:10.1093/jxb/erz276.
79. Levin, E.; Raphael, G.; Ma, J.; Ballester, A.R.; Feygenberg, O.; Norelli, J.; Aly, R.; Gonzalez-Candelas, L.; Wisniewski, M.; Droby, S. Identification and Functional Analysis of NLP-Encoding Genes from the Postharvest Pathogen Penicillium expansum. *Microorganisms* **2019**, *7*, doi:10.3390/microorganisms7060175.
80. Yue, X.; Zhang, G.; Zhao, Z.; Yue, J.; Pu, X.; Sui, M.; Zhan, Y.; Shi, Y.; Wang, Z.; Meng, G., et al. A Cryophyte Transcription Factor, CbABF1, Confers Freezing, and Drought Tolerance in Tobacco. *Front Plant Sci* **2019**, *10*, 699, doi:10.3389/fpls.2019.00699.
81. Ren, Y.; Armstrong, M.; Qi, Y.; McLellan, H.; Zhong, C.; Du, B.; Birch, P.R.J.; Tian, Z. Phytophthora infestans RXLR Effectors Target Parallel Steps in an Immune Signal Transduction Pathway. *Plant Physiol* **2019**, *180*, 2227-2239, doi:10.1104/pp.18.00625.

82. Grossi-de-Sa, M.; Petitot, A.S.; Xavier, D.A.; Sa, M.E.L.; Mezzalira, I.; Beneventi, M.A.; Martins, N.F.; Baimey, H.K.; Albuquerque, E.V.S.; Grossi-de-Sa, M.F., et al. Rice susceptibility to root-knot nematodes is enhanced by the Meloidogyne incognita MSP18 effector gene. *Planta* **2019**, *250*, 1215-1227, doi:10.1007/s00425-019-03205-3.
83. Hunter, J.G.L.; Wilde, S.; Tafoya, A.M.; Horsman, J.; Yousif, M.; Diamos, A.G.; Roland, K.L.; Mason, H.S. Evaluation of a toxoid fusion protein vaccine produced in plants to protect poultry against necrotic enteritis. *PeerJ* **2019**, *7*, e6600, doi:10.7717/peerj.6600.
84. Lan, X.; Liu, Y.; Song, S.; Yin, L.; Xiang, J.; Qu, J.; Lu, J. Plasmopara viticola effector PvRXLR131 suppresses plant immunity by targeting plant receptor-like kinase inhibitor BKI1. *Mol Plant Pathol* **2019**, *20*, 765-783, doi:10.1111/mpp.12790.
85. Bernabe-Orts, J.M.; Casas-Rodrigo, I.; Minguet, E.G.; Landolfi, V.; Garcia-Carpintero, V.; Gianoglio, S.; Vazquez-Vilar, M.; Granell, A.; Orzaez, D. Assessment of Cas12a-mediated gene editing efficiency in plants. *Plant Biotechnol J* **2019**, *17*, 1971-1984, doi:10.1111/pbi.13113.
86. Yusa, A.; Neriya, Y.; Hashimoto, M.; Yoshida, T.; Fujimoto, Y.; Hosoe, N.; Keima, T.; Tokumaru, K.; Maejima, K.; Netsu, O., et al. Functional conservation of EXA1 among diverse plant species for the infection by a family of plant viruses. *Sci Rep* **2019**, *9*, 5958, doi:10.1038/s41598-019-42400-w.
87. Baba, S.A.; Ashraf, N. Functional characterization of flavonoid 3'-hydroxylase, CsF3'H, from Crocus sativus L: Insights into substrate specificity and role in abiotic stress. *Arch Biochem Biophys* **2019**, *667*, 70-78, doi:10.1016/j.abb.2019.04.012.
88. Irmisch, S.; Ruebsam, H.; Jancsik, S.; Man Saint Yuen, M.; Madilao, L.L.; Bohlmann, J. Flavonol Biosynthesis Genes and Their Use in Engineering the Plant Antidiabetic Metabolite Montbretin A. *Plant Physiol* **2019**, *180*, 1277-1290, doi:10.1104/pp.19.00254.
89. Matsuo, K.; Atsumi, G. CRISPR/Cas9-mediated knockout of the RDR6 gene in Nicotiana benthamiana for efficient transient expression of recombinant proteins. *Planta* **2019**, *250*, 463-473, doi:10.1007/s00425-019-03180-9.
90. Pang, E.L.; Peyret, H.; Ramirez, A.; Loh, H.S.; Lai, K.S.; Fang, C.M.; Rosenberg, W.M.; Lomonossoff, G.P. Epitope Presentation of Dengue Viral Envelope Glycoprotein Domain III on Hepatitis B Core Protein Virus-Like Particles Produced in Nicotiana benthamiana. *Front Plant Sci* **2019**, *10*, 455, doi:10.3389/fpls.2019.00455.
91. Schmidt, S.; Smertenko, A. Identification and characterization of the land-plant-specific microtubule nucleation factor MACET4. *J Cell Sci* **2019**, *132*, doi:10.1242/jcs.232819.
92. Sultana, M.M.; Dutta, A.K.; Tanaka, Y.; Aboulela, M.; Nishimura, K.; Sugiura, S.; Niwa, T.; Maeo, K.; Goto-Yamada, S.; Kimura, T., et al. Gateway binary vectors with organelle-targeted fluorescent proteins for highly sensitive reporter assay in gene expression analysis of plants. *J Biotechnol* **2019**, *297*, 19-27, doi:10.1016/j.jbiotec.2019.03.015.
93. Luo, F.; Ling, Y.; Li, D.S.; Tang, T.; Liu, Y.C.; Liu, Y.; Li, S.H. Characterization of a sesquiterpene cyclase from the glandular trichomes of Leucosceptrum canum for sole production of cedrol in Escherichia coli and Nicotiana benthamiana. *Phytochemistry* **2019**, *162*, 121-128, doi:10.1016/j.phytochem.2019.03.009.
94. Gomes, M.; Alvarez, M.A.; Quellis, L.R.; Becher, M.L.; Castro, J.M.A.; Gameiro, J.; Caporrino, M.C.; Moura-da-Silva, A.M.; de Oliveira Santos, M. Expression of an scFv antibody fragment in Nicotiana benthamiana and in vitro assessment of its neutralizing potential against the snake venom metalloproteinase BaP1 from Bothrops asper. *Toxicon* **2019**, *160*, 38-46, doi:10.1016/j.toxicon.2019.02.011.
95. Li, X.; Liang, Y.; Gao, B.; Mijiti, M.; Bozorov, T.A.; Yang, H.; Zhang, D.; Wood, A.J. ScDREB10, an A-5c type of DREB Gene of the Desert Moss Syntrichia caninervis, Confers Osmotic and Salt Tolerances to Arabidopsis. *Genes (Basel)* **2019**, *10*, doi:10.3390/genes10020146.
96. Turnbull, D.; Wang, H.; Breen, S.; Malec, M.; Naqvi, S.; Yang, L.; Welsh, L.; Hemsley, P.; Zhendong, T.; Brunner, F., et al. AVR2 Targets BSL Family Members, Which Act as Susceptibility Factors to Suppress Host Immunity. *Plant Physiol* **2019**, *180*, 571-581, doi:10.1104/pp.18.01143.
97. Deng, S.; Mai, Y.; Shui, L.; Niu, J. WRINKLED1 transcription factor orchestrates the regulation of carbon partitioning for C18:1 (oleic acid) accumulation in Siberian apricot kernel. *Sci Rep* **2019**, *9*, 2693, doi:10.1038/s41598-019-39236-9.

98. Yang, S.; Dai, Y.; Chen, Y.; Yang, J.; Yang, D.; Liu, Q.; Jian, H. A Novel G16B09-Like Effector From Heterodera avenae Suppresses Plant Defenses and Promotes Parasitism. *Front Plant Sci* **2019**, *10*, 66, doi:10.3389/fpls.2019.00066.
99. Vicente, C.S.L.; Nemchinov, L.G.; Mota, M.; Eisenback, J.D.; Kamo, K.; Vieira, P. Identification and characterization of the first pectin methylesterase gene discovered in the root lesion nematode Pratylenchus penetrans. *PLoS One* **2019**, *14*, e0212540, doi:10.1371/journal.pone.0212540.
100. Cai, Y.; Whitehead, P.; Chappell, J.; Chapman, K.D. Mouse lipogenic proteins promote the co-accumulation of triacylglycerols and sesquiterpenes in plant cells. *Planta* **2019**, *250*, 79-94, doi:10.1007/s00425-019-03148-9.
101. Melo, B.P.; Fraga, O.T.; Silva, J.C.F.; Ferreira, D.O.; Brustolini, O.J.B.; Carpinetti, P.A.; Machado, J.P.B.; Reis, P.A.B.; Fontes, E.P.B. Revisiting the Soybean GmNAC Superfamily. *Front Plant Sci* **2018**, *9*, 1864, doi:10.3389/fpls.2018.01864.
102. Wen, Z.; Raffaello, T.; Zeng, Z.; Pavicic, M.; Asiegbu, F.O. Chlorophyll fluorescence imaging for monitoring effects of Heterobasidion parviporum small secreted protein induced cell death and in planta defense gene expression. *Fungal Genet Biol* **2019**, *126*, 37-49, doi:10.1016/j.fgb.2019.02.003.
103. Pecrix, Y.; Buendia, L.; Penouilh-Suzette, C.; Marechaux, M.; Legrand, L.; Bouchez, O.; Rengel, D.; Gouzy, J.; Cottret, L.; Vear, F., et al. Sunflower resistance to multiple downy mildew pathotypes revealed by recognition of conserved effectors of the oomycete Plasmopara halstedii. *Plant J* **2019**, *97*, 730-748, doi:10.1111/tpj.14157.
104. Sheshukova, E.V.; Komarova, T.V.; Ershova, N.M.; Bronstein, A.M.; Dorokhov, Y.L. The Expression of Matryoshka Gene Encoding a Homologue of Kunitz Peptidase Inhibitor Is Regulated Both at the Level of Transcription and Translation. *Biochemistry (Mosc)* **2018**, *83*, 1255-1262, doi:10.1134/S0006297918100103.
105. Sathish, S.; Preethy, K.S.; Venkatesh, R.; Sathishkumar, R. Rapid enhancement of alpha-tocopherol content in Nicotiana benthamiana by transient expression of Arabidopsis thaliana Tocopherol cyclase and Homogentisate phytyl transferase genes. *3 Biotech* **2018**, *8*, 485, doi:10.1007/s13205-018-1496-4.
106. Zheng, X.; Li, X.; Wang, B.; Cheng, D.; Li, Y.; Li, W.; Huang, M.; Tan, X.; Zhao, G.; Song, B., et al. A systematic screen of conserved Ralstonia solanacearum effectors reveals the role of RipAB, a nuclear-localized effector that suppresses immune responses in potato. *Mol Plant Pathol* **2019**, *20*, 547-561, doi:10.1111/mpp.12774.
107. Wang, S.; McLellan, H.; Bukharova, T.; He, Q.; Murphy, F.; Shi, J.; Sun, S.; van Weymers, P.; Ren, Y.; Thilliez, G., et al. Phytophthora infestans RXLR effectors act in concert at diverse subcellular locations to enhance host colonization. *J Exp Bot* **2019**, *70*, 343-356, doi:10.1093/jxb/ery360.
108. Huang, G.; Liu, Z.; Gu, B.; Zhao, H.; Jia, J.; Fan, G.; Meng, Y.; Du, Y.; Shan, W. An RXLR effector secreted by Phytophthora parasitica is a virulence factor and triggers cell death in various plants. *Mol Plant Pathol* **2019**, *20*, 356-371, doi:10.1111/mpp.12760.
109. Pelgrom, A.J.E.; Eikelhof, J.; Elberse, J.; Meisrimler, C.N.; Raedts, R.; Klein, J.; Van den Ackerveken, G. Recognition of lettuce downy mildew effector BLR38 in Lactuca serriola LS102 requires two unlinked loci. *Mol Plant Pathol* **2019**, *20*, 240-253, doi:10.1111/mpp.12751.
110. Sun, X.; Yu, G.; Li, J.; Liu, J.; Wang, X.; Zhu, G.; Zhang, X.; Pan, H. AcERF2, an ethylene-responsive factor of Atriplex canescens, positively modulates osmotic and disease resistance in Arabidopsis thaliana. *Plant Sci* **2018**, *274*, 32-43, doi:10.1016/j.plantsci.2018.05.004.
111. Mei, Y.; Wright, K.M.; Haegeman, A.; Bauters, L.; Diaz-Granados, A.; Goverse, A.; Gheysen, G.; Jones, J.T.; Mantelin, S. The Globodera pallida SPRYSEC Effector GpSPRY-414-2 That Suppresses Plant Defenses Targets a Regulatory Component of the Dynamic Microtubule Network. *Front Plant Sci* **2018**, *9*, 1019, doi:10.3389/fpls.2018.01019.
112. Li, Z.; Tian, Y.; Xu, J.; Fu, X.; Gao, J.; Wang, B.; Han, H.; Wang, L.; Peng, R.; Yao, Q. A tomato ERF transcription factor, SlERF84, confers enhanced tolerance to drought and salt stress but negatively regulates immunity against Pseudomonas syringae pv. tomato DC3000. *Plant Physiol Biochem* **2018**, *132*, 683-695, doi:10.1016/j.plaphy.2018.08.022.

113. Aboobucker, S.I.; Suza, W.P.; Lorence, A. Characterization of Two Arabidopsis L-Gulono-1,4-lactone Oxidases, AtGulLO3 and AtGulLO5, Involved in Ascorbate Biosynthesis. *React Oxyg Species (Apex)* **2017**, *4*, 389-417, doi:10.20455/ros.2017.861.
114. Ofori, P.A.; Geisler, M.; di Donato, M.; Pengchao, H.; Otagaki, S.; Matsumoto, S.; Shiratake, K. Tomato ATP-Binding Cassette Transporter SlABCB4 Is Involved in Auxin Transport in the Developing Fruit. *Plants (Basel)* **2018**, *7*, doi:10.3390/plants7030065.
115. Reis, R.S.; Litholdo, C.G., Jr.; Bally, J.; Roberts, T.H.; Waterhouse, P.M. A conditional silencing suppression system for transient expression. *Sci Rep* **2018**, *8*, 9426, doi:10.1038/s41598-018-27778-3.
116. Naalden, D.; Haegeman, A.; de Almeida-Engler, J.; Birhane Eshetu, F.; Bauters, L.; Gheysen, G. The Meloidogyne graminicola effector Mg16820 is secreted in the apoplast and cytoplasm to suppress plant host defense responses. *Mol Plant Pathol* **2018**, *19*, 2416-2430, doi:10.1111/mpp.12719.
117. Li, W.; Zhou, F.; Pichersky, E. Jasmone Hydroxylase, a Key Enzyme in the Synthesis of the Alcohol Moiety of Pyrethrin Insecticides. *Plant Physiol* **2018**, *177*, 1498-1509, doi:10.1104/pp.18.00748.
118. Daumann, M.; Hickl, D.; Zimmer, D.; DeTar, R.A.; Kunz, H.H.; Mohlmann, T. Characterization of filament-forming CTP synthases from Arabidopsis thaliana. *Plant J* **2018**, *96*, 316-328, doi:10.1111/tpj.14032.
119. Hu, P.; Liu, J.; Xu, J.; Zhou, C.; Cao, S.; Zhou, W.; Huang, Z.; Yuan, S.; Wang, X.; Xiao, J., et al. A malectin-like/leucine-rich repeat receptor protein kinase gene, RLK-V, regulates powdery mildew resistance in wheat. *Mol Plant Pathol* **2018**, *19*, 2561-2574, doi:10.1111/mpp.12729.
120. Zeng, H.; Xie, Y.; Liu, G.; Lin, D.; He, C.; Shi, H. Molecular identification of GAPDHs in cassava highlights the antagonism of MeGAPCs and MeATG8s in plant disease resistance against cassava bacterial blight. *Plant Mol Biol* **2018**, *97*, 201-214, doi:10.1007/s11103-018-0733-x.
121. Yang, J.; Wu, C.; Yu, Y.; Mao, H.; Bao, Y.; Kang, Y.; Qi, Z. A mongolian pine specific endoplasmic reticulum localized CALMODULIN-LIKE calcium binding protein enhances arabidopsis growth. *J Plant Physiol* **2018**, *226*, 1-11, doi:10.1016/j.jplph.2018.04.006.
122. Gimenez-Ibanez, S.; Hann, D.R.; Chang, J.H.; Segonzac, C.; Boller, T.; Rathjen, J.P. Differential Suppression of Nicotiana benthamiana Innate Immune Responses by Transiently Expressed Pseudomonas syringae Type III Effectors. *Front Plant Sci* **2018**, *9*, 688, doi:10.3389/fpls.2018.00688.
123. Sasaki, N.; Takashima, E.; Nyunoya, H. Altered Subcellular Localization of a Tobacco Membrane Raft-Associated Remorin Protein by Tobamovirus Infection and Transient Expression of Viral Replication and Movement Proteins. *Front Plant Sci* **2018**, *9*, 619, doi:10.3389/fpls.2018.00619.
124. Ali, S.; Ahmad Nasir, I.; Rafiq, M.; Javed Butt, S.; Ihsan, F.; Qayyum Rao, A.; Husnain, T. Sugarcane Mosaic Virus-Based Gene Silencing in Nicotiana benthamiana. *Iran J Biotechnol* **2017**, *15*, 260-267, doi:10.15171/ijb.1536.
125. Sun, Q.; Li, J.; Cheng, W.; Guo, H.; Liu, X.; Gao, H. AtPAP2, a Unique Member of the PAP Family, Functions in the Plasma Membrane. *Genes (Basel)* **2018**, *9*, doi:10.3390/genes9050257.
126. Robin, G.P.; Kleemann, J.; Neumann, U.; Cabre, L.; Dallery, J.F.; Lapalu, N.; O'Connell, R.J. Subcellular Localization Screening of Colletotrichum higginsianum Effector Candidates Identifies Fungal Proteins Targeted to Plant Peroxisomes, Golgi Bodies, and Microtubules. *Front Plant Sci* **2018**, *9*, 562, doi:10.3389/fpls.2018.00562.
127. Wang, B.; Wang, G.; Shen, F.; Zhu, S. A Glycine-Rich RNA-Binding Protein, CsGR-RBP3, Is Involved in Defense Responses Against Cold Stress in Harvested Cucumber (Cucumis sativus L.) Fruit. *Front Plant Sci* **2018**, *9*, 540, doi:10.3389/fpls.2018.00540.
128. Shi, Q.; Mao, Z.; Zhang, X.; Zhang, X.; Wang, Y.; Ling, J.; Lin, R.; Li, D.; Kang, X.; Sun, W., et al. A Meloidogyne incognita effector MiISE5 suppresses programmed cell death to promote parasitism in host plant. *Sci Rep* **2018**, *8*, 7256, doi:10.1038/s41598-018-24999-4.
129. Vaira, A.M.; Lim, H.S.; Bauchan, G.; Gulbronson, C.J.; Miozzi, L.; Vinals, N.; Natilla, A.; Hammond, J. The interaction of Lolium latent virus major coat protein with ankyrin repeat protein NbANKr redirects it to chloroplasts and modulates virus infection. *J Gen Virol* **2018**, *99*, 730-742, doi:10.1099/jgv.0.001043.

130. Pan, Y.; Wei, J.; Yao, C.; Reng, H.; Gao, Z. SsSm1, a Cerato-platanin family protein, is involved in the hyphal development and pathogenic process of Sclerotinia sclerotiorum. *Plant Sci* **2018**, *270*, 37-46, doi:10.1016/j.plantsci.2018.02.001.
131. Murphy, F.; He, Q.; Armstrong, M.; Giuliani, L.M.; Boevink, P.C.; Zhang, W.; Tian, Z.; Birch, P.R.J.; Gilroy, E.M. The Potato MAP3K StVIK Is Required for the Phytophthora infestans RXLR Effector Pi17316 to Promote Disease. *Plant Physiol* **2018**, *177*, 398-410, doi:10.1104/pp.18.00028.
132. Zhao, Q.; Xiang, X.; Liu, D.; Yang, A.; Wang, Y. Tobacco Transcription Factor NtbHLH123 Confers Tolerance to Cold Stress by Regulating the NtCBF Pathway and Reactive Oxygen Species Homeostasis. *Front Plant Sci* **2018**, *9*, 381, doi:10.3389/fpls.2018.00381.
133. Wei, Y.; Liu, G.; Chang, Y.; He, C.; Shi, H. Heat shock transcription factor 3 regulates plant immune response through modulation of salicylic acid accumulation and signalling in cassava. *Mol Plant Pathol* **2018**, *19*, 2209-2220, doi:10.1111/mpp.12691.
134. Bradai, M.; Mahjoubi, H.; Chini, A.; Chaboute, M.E.; Hanin, M.; Ebel, C. Genome wide identification of wheat and Brachypodium type one protein phosphatases and functional characterization of durum wheat TdPP1a. *PLoS One* **2018**, *13*, e0191272, doi:10.1371/journal.pone.0191272.
135. Siwinska, J.; Siatkowska, K.; Olry, A.; Grosjean, J.; Hehn, A.; Bourgaud, F.; Meharg, A.A.; Carey, M.; Lojkowska, E.; Ihnatowicz, A. Scopoletin 8-hydroxylase: a novel enzyme involved in coumarin biosynthesis and iron-deficiency responses in Arabidopsis. *J Exp Bot* **2018**, *69*, 1735-1748, doi:10.1093/jxb/ery005.
136. Liu, T.Y.; Chou, W.C.; Chen, W.Y.; Chu, C.Y.; Dai, C.Y.; Wu, P.Y. Detection of membrane protein-protein interaction in planta based on dual-intein-coupled tripartite split-GFP association. *Plant J* **2018**, *94*, 426-438, doi:10.1111/tpj.13874.
137. Pitino, M.; Allen, V.; Duan, Y. LasDelta5315 Effector Induces Extreme Starch Accumulation and Chlorosis as Ca. Liberibacter asiaticus Infection in Nicotiana benthamiana. *Front Plant Sci* **2018**, *9*, 113, doi:10.3389/fpls.2018.00113.
138. Yan, Y.; He, X.; Hu, W.; Liu, G.; Wang, P.; He, C.; Shi, H. Functional analysis of MeCIPK23 and MeCBL1/9 in cassava defense response against Xanthomonas axonopodis pv. manihotis. *Plant Cell Rep* **2018**, *37*, 887-900, doi:10.1007/s00299-018-2276-7.
139. Wang, B.; Song, N.; Zhang, Q.; Wang, N.; Kang, Z. TaMAPK4 Acts as a Positive Regulator in Defense of Wheat Stripe-Rust Infection. *Front Plant Sci* **2018**, *9*, 152, doi:10.3389/fpls.2018.00152.
140. Alcantara, A.; Seitner, D.; Navarrete, F.; Djamei, A. A high-throughput screening method to identify proteins involved in unfolded protein response of the endoplasmic reticulum in plants. *Plant Methods* **2020**, *16*, 4, doi:10.1186/s13007-020-0552-3.
141. Zhang, Q.; Wang, B.; Wei, J.; Wang, X.; Han, Q.; Kang, Z. TaNTF2, a contributor for wheat resistance to the stripe rust pathogen. *Plant Physiol Biochem* **2018**, *123*, 260-267, doi:10.1016/j.plaphy.2017.12.020.
142. Erickson, J.L.; Adlung, N.; Lampe, C.; Bonas, U.; Schattat, M.H. The Xanthomonas effector XopL uncovers the role of microtubules in stromule extension and dynamics in Nicotiana benthamiana. *Plant J* **2018**, *93*, 856-870, doi:10.1111/tpj.13813.
143. An, J.P.; Li, R.; Qu, F.J.; You, C.X.; Wang, X.F.; Hao, Y.J. An apple NAC transcription factor negatively regulates cold tolerance via CBF-dependent pathway. *J Plant Physiol* **2018**, *221*, 74-80, doi:10.1016/j.jplph.2017.12.009.
144. Olmedo, P.; Moreno, A.A.; Sanhueza, D.; Balic, I.; Silva-Sanzana, C.; Zepeda, B.; Verdonk, J.C.; Arriagada, C.; Meneses, C.; Campos-Vargas, R. A catechol oxidase AcPPO from cherimoya (Annona cherimola Mill.) is localized to the Golgi apparatus. *Plant Sci* **2018**, *266*, 46-54, doi:10.1016/j.plantsci.2017.10.012.
145. Ji, X.J.; Mao, X.; Hao, Q.T.; Liu, B.L.; Xue, J.A.; Li, R.Z. Splice Variants of the Castor WRI1 Gene Upregulate Fatty Acid and Oil Biosynthesis When Expressed in Tobacco Leaves. *Int J Mol Sci* **2018**, *19*, doi:10.3390/ijms19010146.
146. Poon, S.; Harris, K.S.; Jackson, M.A.; McCorkelle, O.C.; Gilding, E.K.; Durek, T.; van der Weerden, N.L.; Craik, D.J.; Anderson, M.A. Co-expression of a cyclizing asparaginyl endopeptidase enables

efficient production of cyclic peptides in planta. *J Exp Bot* **2018**, *69*, 633-641, doi:10.1093/jxb/erx422.
147. Yin, J.; Gu, B.; Huang, G.; Tian, Y.; Quan, J.; Lindqvist-Kreuze, H.; Shan, W. Conserved RXLR Effector Genes of Phytophthora infestans Expressed at the Early Stage of Potato Infection Are Suppressive to Host Defense. *Front Plant Sci* **2017**, *8*, 2155, doi:10.3389/fpls.2017.02155.
148. Wang, L.; Tan, H.; Wu, M.; Jimenez-Gongora, T.; Tan, L.; Lozano-Duran, R. Dynamic Virus-Dependent Subnuclear Localization of the Capsid Protein from a Geminivirus. *Front Plant Sci* **2017**, *8*, 2165, doi:10.3389/fpls.2017.02165.
149. Choi, S.; Jayaraman, J.; Segonzac, C.; Park, H.J.; Park, H.; Han, S.W.; Sohn, K.H. Pseudomonas syringae pv. actinidiae Type III Effectors Localized at Multiple Cellular Compartments Activate or Suppress Innate Immune Responses in Nicotiana benthamiana. *Front Plant Sci* **2017**, *8*, 2157, doi:10.3389/fpls.2017.02157.
150. Kumar, R.M.S.; Ji, G.; Guo, H.; Zhao, L.; Zheng, B. Over-expression of a grafting-responsive gene from hickory increases abiotic stress tolerance in Arabidopsis. *Plant Cell Rep* **2018**, *37*, 541-552, doi:10.1007/s00299-018-2250-4.
151. An, J.P.; Yao, J.F.; Wang, X.N.; You, C.X.; Wang, X.F.; Hao, Y.J. MdHY5 positively regulates cold tolerance via CBF-dependent and CBF-independent pathways in apple. *J Plant Physiol* **2017**, *218*, 275-281, doi:10.1016/j.jplph.2017.09.001.
152. Yan, Y.; Wang, P.; He, C.; Shi, H. MeWRKY20 and its interacting and activating autophagy-related protein 8 (MeATG8) regulate plant disease resistance in cassava. *Biochem Biophys Res Commun* **2017**, *494*, 20-26, doi:10.1016/j.bbrc.2017.10.091.
153. Rattanapisit, K.; Abdulheem, S.; Chaikeawkaew, D.; Kubera, A.; Mason, H.S.; Ma, J.K.; Pavasant, P.; Phoolcharoen, W. Recombinant human osteopontin expressed in Nicotiana benthamiana stimulates osteogenesis related genes in human periodontal ligament cells. *Sci Rep* **2017**, *7*, 17358, doi:10.1038/s41598-017-17666-7.
154. Chen, X.R.; Huang, S.X.; Zhang, Y.; Sheng, G.L.; Zhang, B.Y.; Li, Q.Y.; Zhu, F.; Xu, J.Y. Transcription profiling and identification of infection-related genes in Phytophthora cactorum. *Mol Genet Genomics* **2018**, *293*, 541-555, doi:10.1007/s00438-017-1400-7.
155. Almeida, A.; Dong, L.; Khakimov, B.; Bassard, J.E.; Moses, T.; Lota, F.; Goossens, A.; Appendino, G.; Bak, S. A Single Oxidosqualene Cyclase Produces the Seco-Triterpenoid alpha-Onocerin. *Plant Physiol* **2018**, *176*, 1469-1484, doi:10.1104/pp.17.01369.
156. Zhu, X.; Xiao, K.; Cui, H.; Hu, J. Overexpression of the Prunus sogdiana NBS-LRR Subgroup Gene PsoRPM2 Promotes Resistance to the Root-Knot Nematode Meloidogyne incognita in Tobacco. *Front Microbiol* **2017**, *8*, 2113, doi:10.3389/fmicb.2017.02113.
157. Yang, T.; Fang, L.; Sanders, S.; Jayanthi, S.; Rajan, G.; Podicheti, R.; Thallapuranam, S.K.; Mockaitis, K.; Medina-Bolivar, F. Stilbenoid prenyltransferases define key steps in the diversification of peanut phytoalexins. *J Biol Chem* **2018**, *293*, 28-46, doi:10.1074/jbc.RA117.000564.
158. Brown, D.; Feeney, M.; Ahmadi, M.; Lonoce, C.; Sajari, R.; Di Cola, A.; Frigerio, L. Subcellular localization and interactions among rubber particle proteins from Hevea brasiliensis. *J Exp Bot* **2017**, *68*, 5045-5055, doi:10.1093/jxb/erx331.
159. Wei, Y.; Liu, G.; Bai, Y.; Xia, F.; He, C.; Shi, H.; Foyer, C. Two transcriptional activators of N-acetylserotonin O-methyltransferase 2 and melatonin biosynthesis in cassava. *J Exp Bot* **2017**, *68*, 4997-5006, doi:10.1093/jxb/erx305.
160. Paskevicius, S.; Starkevic, U.; Misiunas, A.; Vitkauskiene, A.; Gleba, Y.; Razanskiene, A. Plant-expressed pyocins for control of Pseudomonas aeruginosa. *PLoS One* **2017**, *12*, e0185782, doi:10.1371/journal.pone.0185782.
161. Mamedov, T.; Cicek, K.; Gulec, B.; Ungor, R.; Hasanova, G. In vivo production of non-glycosylated recombinant proteins in Nicotiana benthamiana plants by co-expression with Endo-beta-N-acetylglucosaminidase H (Endo H) of Streptomyces plicatus. *PLoS One* **2017**, *12*, e0183589, doi:10.1371/journal.pone.0183589.
162. Larrimore, K.E.; Kazan, I.C.; Kannan, L.; Kendle, R.P.; Jamal, T.; Barcus, M.; Bolia, A.; Brimijoin, S.; Zhan, C.G.; Ozkan, S.B., et al. Plant-expressed cocaine hydrolase variants of

butyrylcholinesterase exhibit altered allosteric effects of cholinesterase activity and increased inhibitor sensitivity. *Sci Rep* **2017**, *7*, 10419, doi:10.1038/s41598-017-10571-z.
163. Kettles, G.J.; Bayon, C.; Sparks, C.A.; Canning, G.; Kanyuka, K.; Rudd, J.J. Characterization of an antimicrobial and phytotoxic ribonuclease secreted by the fungal wheat pathogen Zymoseptoria tritici. *New Phytol* **2018**, *217*, 320-331, doi:10.1111/nph.14786.
164. Fresquet-Corrales, S.; Roque, E.; Sarrion-Perdigones, A.; Rochina, M.; Lopez-Gresa, M.P.; Diaz-Mula, H.M.; Belles, J.M.; Tomas-Barberan, F.; Beltran, J.P.; Canas, L.A. Metabolic engineering to simultaneously activate anthocyanin and proanthocyanidin biosynthetic pathways in Nicotiana spp. *PLoS One* **2017**, *12*, e0184839, doi:10.1371/journal.pone.0184839.
165. Baudin, M.; Hassan, J.A.; Schreiber, K.J.; Lewis, J.D. Analysis of the ZAR1 Immune Complex Reveals Determinants for Immunity and Molecular Interactions. *Plant Physiol* **2017**, *174*, 2038-2053, doi:10.1104/pp.17.00441.
166. Fang, Y.L.; Peng, Y.L.; Fan, J. The Nep1-like protein family of Magnaporthe oryzae is dispensable for the infection of rice plants. *Sci Rep* **2017**, *7*, 4372, doi:10.1038/s41598-017-04430-0.
167. Huang, A.C.; Kautsar, S.A.; Hong, Y.J.; Medema, M.H.; Bond, A.D.; Tantillo, D.J.; Osbourn, A. Unearthing a sesterterpene biosynthetic repertoire in the Brassicaceae through genome mining reveals convergent evolution. *Proc Natl Acad Sci U S A* **2017**, *114*, E6005-E6014, doi:10.1073/pnas.1705567114.
168. Patel, J.; Ariyaratne, M.; Ahmed, S.; Ge, L.; Phuntumart, V.; Kalinoski, A.; Morris, P.F. Dual functioning of plant arginases provides a third route for putrescine synthesis. *Plant Sci* **2017**, *262*, 62-73, doi:10.1016/j.plantsci.2017.05.011.
169. Andersen-Ranberg, J.; Kongstad, K.T.; Nafisi, M.; Staerk, D.; Okkels, F.T.; Mortensen, U.H.; Lindberg Moller, B.; Frandsen, R.J.N.; Kannangara, R. Synthesis of C-Glucosylated Octaketide Anthraquinones in Nicotiana benthamiana by Using a Multispecies-Based Biosynthetic Pathway. *Chembiochem* **2017**, *18*, 1893-1897, doi:10.1002/cbic.201700331.
170. Liu, Y.; Ke, L.; Wu, G.; Xu, Y.; Wu, X.; Xia, R.; Deng, X.; Xu, Q. miR3954 is a trigger of phasiRNAs that affects flowering time in citrus. *Plant J* **2017**, *92*, 263-275, doi:10.1111/tpj.13650.
171. Luck, K.; Jia, Q.; Huber, M.; Handrick, V.; Wong, G.K.; Nelson, D.R.; Chen, F.; Gershenzon, J.; Kollner, T.G. CYP79 P450 monooxygenases in gymnosperms: CYP79A118 is associated with the formation of taxiphyllin in Taxus baccata. *Plant Mol Biol* **2017**, *95*, 169-180, doi:10.1007/s11103-017-0646-0.
172. Raffaello, T.; Asiegbu, F.O. Small secreted proteins from the necrotrophic conifer pathogen Heterobasidion annosum s.l. (HaSSPs) induce cell death in Nicotiana benthamiana. *Sci Rep* **2017**, *7*, 8000, doi:10.1038/s41598-017-08010-0.
173. Adkar-Purushothama, C.R.; Iyer, P.S.; Perreault, J.P. Potato spindle tuber viroid infection triggers degradation of chloride channel protein CLC-b-like and Ribosomal protein S3a-like mRNAs in tomato plants. *Sci Rep* **2017**, *7*, 8341, doi:10.1038/s41598-017-08823-z.
174. Liu, J.; Cheng, X.; Liu, P.; Sun, J. miR156-Targeted SBP-Box Transcription Factors Interact with DWARF53 to Regulate TEOSINTE BRANCHED1 and BARREN STALK1 Expression in Bread Wheat. *Plant Physiol* **2017**, *174*, 1931-1948, doi:10.1104/pp.17.00445.
175. Li, Y.; Wang, H.; Li, X.; Liang, G.; Yu, D. Two DELLA-interacting proteins bHLH48 and bHLH60 regulate flowering under long-day conditions in Arabidopsis thaliana. *J Exp Bot* **2017**, *68*, 2757-2767, doi:10.1093/jxb/erx143.
176. Fan, Z.Q.; Tan, X.L.; Shan, W.; Kuang, J.F.; Lu, W.J.; Chen, J.Y. BrWRKY65, a WRKY Transcription Factor, Is Involved in Regulating Three Leaf Senescence-Associated Genes in Chinese Flowering Cabbage. *Int J Mol Sci* **2017**, *18*, doi:10.3390/ijms18061228.
177. Forman, V.; Callari, R.; Folly, C.; Heider, H.; Hamberger, B. Production of Putative Diterpene Carboxylic Acid Intermediates of Triptolide in Yeast. *Molecules* **2017**, *22*, doi:10.3390/molecules22060981.
178. Peng, Q.; Su, Y.; Ling, H.; Ahmad, W.; Gao, S.; Guo, J.; Que, Y.; Xu, L. A sugarcane pathogenesis-related protein, ScPR10, plays a positive role in defense responses under Sporisorium scitamineum, SrMV, SA, and MeJA stresses. *Plant Cell Rep* **2017**, *36*, 1427-1440, doi:10.1007/s00299-017-2166-4.

179. Ramos-Sanchez, J.M.; Triozzi, P.M.; Moreno-Cortes, A.; Conde, D.; Perales, M.; Allona, I. Real-time monitoring of PtaHMGB activity in poplar transactivation assays. *Plant Methods* **2017**, *13*, 50, doi:10.1186/s13007-017-0199-x.
180. Chen, K.; Liu, H.; Lou, Q.; Liu, Y. Ectopic Expression of the Grape Hyacinth (Muscari armeniacum) R2R3-MYB Transcription Factor Gene, MaAN2, Induces Anthocyanin Accumulation in Tobacco. *Front Plant Sci* **2017**, *8*, 965, doi:10.3389/fpls.2017.00965.
181. Yauk, Y.K.; Souleyre, E.J.F.; Matich, A.J.; Chen, X.; Wang, M.Y.; Plunkett, B.; Dare, A.P.; Espley, R.V.; Tomes, S.; Chagne, D., et al. Alcohol acyl transferase 1 links two distinct volatile pathways that produce esters and phenylpropenes in apple fruit. *Plant J* **2017**, *91*, 292-305, doi:10.1111/tpj.13564.
182. Franco-Orozco, B.; Berepiki, A.; Ruiz, O.; Gamble, L.; Griffe, L.L.; Wang, S.; Birch, P.R.J.; Kanyuka, K.; Avrova, A. A new proteinaceous pathogen-associated molecular pattern (PAMP) identified in Ascomycete fungi induces cell death in Solanaceae. *New Phytol* **2017**, *214*, 1657-1672, doi:10.1111/nph.14542.
183. Beloshistov, R.E.; Dreizler, K.; Galiullina, R.A.; Tuzhikov, A.I.; Serebryakova, M.V.; Reichardt, S.; Shaw, J.; Taliansky, M.E.; Pfannstiel, J.; Chichkova, N.V., et al. Phytaspase-mediated precursor processing and maturation of the wound hormone systemin. *New Phytol* **2018**, *218*, 1167-1178, doi:10.1111/nph.14568.
184. Widana Gamage, S.M.K.; Dietzgen, R.G. Intracellular Localization, Interactions and Functions of Capsicum Chlorosis Virus Proteins. *Front Microbiol* **2017**, *8*, 612, doi:10.3389/fmicb.2017.00612.
185. Ponndorf, D.; Broer, I.; Nausch, H. Expression of CphB- and CphE-type cyanophycinases in cyanophycin-producing tobacco and comparison of their ability to degrade cyanophycin in plant and plant extracts. *Transgenic Res* **2017**, *26*, 491-499, doi:10.1007/s11248-017-0019-0.
186. Fang, X.; Li, C.Y.; Yang, Y.; Cui, M.Y.; Chen, X.Y.; Yang, L. Identification of a Novel (-)-5-Epieremophilene Synthase from Salvia miltiorrhiza via Transcriptome Mining. *Front Plant Sci* **2017**, *8*, 627, doi:10.3389/fpls.2017.00627.
187. DeFalco, T.A.; Toyota, M.; Phan, V.; Karia, P.; Moeder, W.; Gilroy, S.; Yoshioka, K. Using GCaMP3 to Study Ca2+ Signaling in Nicotiana Species. *Plant Cell Physiol* **2017**, *58*, 1173-1184, doi:10.1093/pcp/pcx053.
188. Ekchaweng, K.; Evangelisti, E.; Schornack, S.; Tian, M.; Churngchow, N. The plant defense and pathogen counterdefense mediated by Hevea brasiliensis serine protease HbSPA and Phytophthora palmivora extracellular protease inhibitor PpEPI10. *PLoS One* **2017**, *12*, e0175795, doi:10.1371/journal.pone.0175795.
189. Fantino, E.; Segretin, M.E.; Santin, F.; Mirkin, F.G.; Ulloa, R.M. Analysis of the potato calcium-dependent protein kinase family and characterization of StCDPK7, a member induced upon infection with Phytophthora infestans. *Plant Cell Rep* **2017**, *36*, 1137-1157, doi:10.1007/s00299-017-2144-x.
190. Brendolise, C.; Montefiori, M.; Dinis, R.; Peeters, N.; Storey, R.D.; Rikkerink, E.H. A novel hairpin library-based approach to identify NBS-LRR genes required for effector-triggered hypersensitive response in Nicotiana benthamiana. *Plant Methods* **2017**, *13*, 32, doi:10.1186/s13007-017-0181-7.
191. Chen, Z.; Yuan, Y.; Fu, D.; Shen, C.; Yang, Y. Identification and Expression Profiling of the Auxin Response Factors in Dendrobium officinale under Abiotic Stresses. *Int J Mol Sci* **2017**, *18*, doi:10.3390/ijms18050927.
192. Wang, H.; Ren, Y.; Zhou, J.; Du, J.; Hou, J.; Jiang, R.; Wang, H.; Tian, Z.; Xie, C. The Cell Death Triggered by the Nuclear Localized RxLR Effector PITG_22798 from Phytophthora infestans Is Suppressed by the Effector AVR3b. *Int J Mol Sci* **2017**, *18*, doi:10.3390/ijms18020409.
193. Pateraki, I.; Andersen-Ranberg, J.; Jensen, N.B.; Wubshet, S.G.; Heskes, A.M.; Forman, V.; Hallström, B.; Hamberger, B.; Motawia, M.S.; Olsen, C.E., et al. Total biosynthesis of the cyclic AMP booster forskolin from Coleus forskohlii. *eLife* **2017**, *6*, e23001, doi:10.7554/eLife.23001.
194. Turnbull, D.; Yang, L.; Naqvi, S.; Breen, S.; Welsh, L.; Stephens, J.; Morris, J.; Boevink, P.C.; Hedley, P.E.; Zhan, J., et al. RXLR Effector AVR2 Up-Regulates a Brassinosteroid-Responsive bHLH Transcription Factor to Suppress Immunity. *Plant Physiol* **2017**, *174*, 356-369, doi:10.1104/pp.16.01804.

195. Zhan, Y.; Sun, X.; Rong, G.; Hou, C.; Huang, Y.; Jiang, D.; Weng, X. Identification of two transcription factors activating the expression of OsXIP in rice defence response. *BMC Biotechnol* **2017**, *17*, 26, doi:10.1186/s12896-017-0344-7.
196. Ahmed, S.; Ariyaratne, M.; Patel, J.; Howard, A.E.; Kalinoski, A.; Phuntumart, V.; Morris, P.F. Altered expression of polyamine transporters reveals a role for spermidine in the timing of flowering and other developmental response pathways. *Plant Sci* **2017**, *258*, 146-155, doi:10.1016/j.plantsci.2016.12.002.
197. Gushchin, V.A.; Karlin, D.G.; Makhotenko, A.V.; Khromov, A.V.; Erokhina, T.N.; Solovyev, A.G.; Morozov, S.Y.; Agranovsky, A.A. A conserved region in the Closterovirus 1a polyprotein drives extensive remodeling of endoplasmic reticulum membranes and induces motile globules in Nicotiana benthamiana cells. *Virology* **2017**, *502*, 106-113, doi:10.1016/j.virol.2016.12.006.
198. Xu, B.; Lei, L.; Zhu, X.; Zhou, Y.; Xiao, Y. Identification and characterization of L-lysine decarboxylase from Huperzia serrata and its role in the metabolic pathway of lycopodium alkaloid. *Phytochemistry* **2017**, *136*, 23-30, doi:10.1016/j.phytochem.2016.12.022.
199. An, D.; Kim, H.; Ju, S.; Go, Y.S.; Kim, H.U.; Suh, M.C. Expression of Camelina WRINKLED1 Isoforms Rescue the Seed Phenotype of the Arabidopsis wri1 Mutant and Increase the Triacylglycerol Content in Tobacco Leaves. *Front Plant Sci* **2017**, *8*, 34, doi:10.3389/fpls.2017.00034.
200. Klein, A.P.; Sattely, E.S. Biosynthesis of cabbage phytoalexins from indole glucosinolate. *Proc Natl Acad Sci U S A* **2017**, *114*, 1910-1915, doi:10.1073/pnas.1615625114.
201. Yang, B.; Wang, Q.; Jing, M.; Guo, B.; Wu, J.; Wang, H.; Wang, Y.; Lin, L.; Wang, Y.; Ye, W., et al. Distinct regions of the Phytophthora essential effector Avh238 determine its function in cell death activation and plant immunity suppression. *New Phytol* **2017**, *214*, 361-375, doi:10.1111/nph.14430.
202. Yin, J.L.; Wong, W.S.; Jang, I.C.; Chua, N.H. Co-expression of peppermint geranyl diphosphate synthase small subunit enhances monoterpene production in transgenic tobacco plants. *New Phytol* **2017**, *213*, 1133-1144, doi:10.1111/nph.14280.
203. Jimenez-Lopez, J.C.; Melser, S.; DeBoer, K.; Thatcher, L.F.; Kamphuis, L.G.; Foley, R.C.; Singh, K.B. Narrow-Leafed Lupin (Lupinus angustifolius) beta1- and beta6-Conglutin Proteins Exhibit Antifungal Activity, Protecting Plants against Necrotrophic Pathogen Induced Damage from Sclerotinia sclerotiorum and Phytophthora nicotianae. *Front Plant Sci* **2016**, *7*, 1856, doi:10.3389/fpls.2016.01856.
204. Adlung, N.; Prochaska, H.; Thieme, S.; Banik, A.; Bluher, D.; John, P.; Nagel, O.; Schulze, S.; Gantner, J.; Delker, C., et al. Non-host Resistance Induced by the Xanthomonas Effector XopQ Is Widespread within the Genus Nicotiana and Functionally Depends on EDS1. *Front Plant Sci* **2016**, *7*, 1796, doi:10.3389/fpls.2016.01796.
205. Malik, H.J.; Raza, A.; Amin, I.; Scheffler, J.A.; Scheffler, B.E.; Brown, J.K.; Mansoor, S. RNAi-mediated mortality of the whitefly through transgenic expression of double-stranded RNA homologous to acetylcholinesterase and ecdysone receptor in tobacco plants. *Sci Rep* **2016**, *6*, 38469, doi:10.1038/srep38469.
206. Knop, K.; Stepien, A.; Barciszewska-Pacak, M.; Taube, M.; Bielewicz, D.; Michalak, M.; Borst, J.W.; Jarmolowski, A.; Szweykowska-Kulinska, Z. Active 5' splice sites regulate the biogenesis efficiency of Arabidopsis microRNAs derived from intron-containing genes. *Nucleic Acids Res* **2017**, *45*, 2757-2775, doi:10.1093/nar/gkw895.
207. Kim, H.S.; Park, S.C.; Ji, C.Y.; Park, S.; Jeong, J.C.; Lee, H.S.; Kwak, S.S. Molecular characterization of biotic and abiotic stress-responsive MAP kinase genes, IbMPK3 and IbMPK6, in sweetpotato. *Plant Physiol Biochem* **2016**, *108*, 37-48, doi:10.1016/j.plaphy.2016.06.036.
208. Wei, Y.; Hu, W.; Wang, Q.; Liu, W.; Wu, C.; Zeng, H.; Yan, Y.; Li, X.; He, C.; Shi, H. Comprehensive transcriptional and functional analyses of melatonin synthesis genes in cassava reveal their novel role in hypersensitive-like cell death. *Sci Rep* **2016**, *6*, 35029, doi:10.1038/srep35029.
209. Gao, S.; Yu, H.N.; Wu, Y.F.; Liu, X.Y.; Cheng, A.X.; Lou, H.X. Cloning and functional characterization of a phenolic acid decarboxylase from the liverwort Conocephalum japonicum. *Biochem Biophys Res Commun* **2016**, *481*, 239-244, doi:10.1016/j.bbrc.2016.10.131.

210. Darbani, B.; Motawia, M.S.; Olsen, C.E.; Nour-Eldin, H.H.; Moller, B.L.; Rook, F. The biosynthetic gene cluster for the cyanogenic glucoside dhurrin in Sorghum bicolor contains its co-expressed vacuolar MATE transporter. *Sci Rep* **2016**, *6*, 37079, doi:10.1038/srep37079.
211. Atabekova, A.K.; Pankratenko, A.V.; Makarova, S.S.; Lazareva, E.A.; Owens, R.A.; Solovyev, A.G.; Morozov, S.Y. Phylogenetic and functional analyses of a plant protein related to human B-cell receptor-associated proteins. *Biochimie* **2017**, *132*, 28-37, doi:10.1016/j.biochi.2016.10.009.
212. Pelot, K.A.; Mitchell, R.; Kwon, M.; Hagelthorn, D.M.; Wardman, J.F.; Chiang, A.; Bohlmann, J.; Ro, D.K.; Zerbe, P. Biosynthesis of the psychotropic plant diterpene salvinorin A: Discovery and characterization of the Salvia divinorum clerodienyl diphosphate synthase. *Plant J* **2017**, *89*, 885-897, doi:10.1111/tpj.13427.
213. Peng, X.; Wang, H.; Jang, J.C.; Xiao, T.; He, H.; Jiang, D.; Tang, X. OsWRKY80-OsWRKY4 Module as a Positive Regulatory Circuit in Rice Resistance Against Rhizoctonia solani. *Rice (N Y)* **2016**, *9*, 63, doi:10.1186/s12284-016-0137-y.
214. Chen, J.; Deng, F.; Deng, M.; Han, J.; Chen, J.; Wang, L.; Yan, S.; Tong, K.; Liu, F.; Tian, M. Identification and Characterization of a Chloroplast-Targeted Obg GTPase in Dendrobium officinale. *DNA Cell Biol* **2016**, *35*, 802-811, doi:10.1089/dna.2016.3413.
215. Bao, W.; Cao, B.; Zhang, Y.; Wuriyanghan, H. Silencing of Mythimna separata chitinase genes via oral delivery of in planta-expressed RNAi effectors from a recombinant plant virus. *Biotechnol Lett* **2016**, *38*, 1961-1966, doi:10.1007/s10529-016-2186-0.
216. Boyle, P.C.; Schwizer, S.; Hind, S.R.; Kraus, C.M.; De la Torre Diaz, S.; He, B.; Martin, G.B. Detecting N-myristoylation and S-acylation of host and pathogen proteins in plants using click chemistry. *Plant Methods* **2016**, *12*, 38, doi:10.1186/s13007-016-0138-2.
217. Qiu, A.; Liu, Z.; Li, J.; Chen, Y.; Guan, D.; He, S. The Ectopic Expression of CaRop1 Modulates the Response of Tobacco Plants to Ralstonia solanacearum and Aphids. *Front Plant Sci* **2016**, *7*, 1177, doi:10.3389/fpls.2016.01177.
218. Kettles, G.J.; Bayon, C.; Canning, G.; Rudd, J.J.; Kanyuka, K. Apoplastic recognition of multiple candidate effectors from the wheat pathogen Zymoseptoria tritici in the nonhost plant Nicotiana benthamiana. *New Phytol* **2017**, *213*, 338-350, doi:10.1111/nph.14215.
219. Reuter, L.; Ritala, A.; Linder, M.; Joensuu, J. Novel Hydrophobin Fusion Tags for Plant-Produced Fusion Proteins. *PLoS One* **2016**, *11*, e0164032, doi:10.1371/journal.pone.0164032.
220. Hwang, S.T.; Choi, D. A novel rice protein family of OsHIGDs may be involved in early signalling of hypoxia-promoted stem growth in deepwater rice. *Plant Cell Rep* **2016**, *35*, 2021-2031, doi:10.1007/s00299-016-2013-z.
221. Sharpee, W.; Oh, Y.; Yi, M.; Franck, W.; Eyre, A.; Okagaki, L.H.; Valent, B.; Dean, R.A. Identification and characterization of suppressors of plant cell death (SPD) effectors from Magnaporthe oryzae. *Mol Plant Pathol* **2017**, *18*, 850-863, doi:10.1111/mpp.12449.
222. Hewezi, T.; Piya, S.; Qi, M.; Balasubramaniam, M.; Rice, J.H.; Baum, T.J. Arabidopsis miR827 mediates post-transcriptional gene silencing of its ubiquitin E3 ligase target gene in the syncytium of the cyst nematode Heterodera schachtii to enhance susceptibility. *Plant J* **2016**, *88*, 179-192, doi:10.1111/tpj.13238.
223. Khunjan, U.; Ekchaweng, K.; Panrat, T.; Tian, M.; Churngchow, N. Molecular Cloning of HbPR-1 Gene from Rubber Tree, Expression of HbPR-1 Gene in Nicotiana benthamiana and Its Inhibition of Phytophthora palmivora. *PLoS One* **2016**, *11*, e0157591, doi:10.1371/journal.pone.0157591.
224. Vivancos, J.; Deshmukh, R.; Gregoire, C.; Remus-Borel, W.; Belzile, F.; Belanger, R.R. Identification and characterization of silicon efflux transporters in horsetail (Equisetum arvense). *J Plant Physiol* **2016**, *200*, 82-89, doi:10.1016/j.jplph.2016.06.011.
225. Matousek, J.; Kocabek, T.; Patzak, J.; Briza, J.; Siglova, K.; Mishra, A.K.; Duraisamy, G.S.; Tycova, A.; Ono, E.; Krofta, K. The "putative" role of transcription factors from HlWRKY family in the regulation of the final steps of prenylflavonid and bitter acids biosynthesis in hop (Humulus lupulus L.). *Plant Mol Biol* **2016**, *92*, 263-277, doi:10.1007/s11103-016-0510-7.
226. Liu, J.; Guan, T.; Zheng, P.; Chen, L.; Yang, Y.; Huai, B.; Li, D.; Chang, Q.; Huang, L.; Kang, Z. An extracellular Zn-only superoxide dismutase from Puccinia striiformis confers enhanced resistance to host-derived oxidative stress. *Environ Microbiol* **2016**, *18*, 4118-4135, doi:10.1111/1462-2920.13451.

227. Xie, J.; Li, S.; Mo, C.; Wang, G.; Xiao, X.; Xiao, Y. A Novel Meloidogyne incognita Effector Misp12 Suppresses Plant Defense Response at Latter Stages of Nematode Parasitism. *Front Plant Sci* **2016**, *7*, 964, doi:10.3389/fpls.2016.00964.
228. Pitino, M.; Armstrong, C.M.; Cano, L.M.; Duan, Y. Transient Expression of Candidatus Liberibacter Asiaticus Effector Induces Cell Death in Nicotiana benthamiana. *Front Plant Sci* **2016**, *7*, 982, doi:10.3389/fpls.2016.00982.
229. Cao, S.; Chen, H.; Zhang, C.; Tang, Y.; Liu, J.; Qi, H. Heterologous Expression and Biochemical Characterization of Two Lipoxygenases in Oriental Melon, Cucumis melo var. makuwa Makino. *PLoS One* **2016**, *11*, e0153801, doi:10.1371/journal.pone.0153801.
230. Mukaihara, T.; Hatanaka, T.; Nakano, M.; Oda, K. Ralstonia solanacearum Type III Effector RipAY Is a Glutathione-Degrading Enzyme That Is Activated by Plant Cytosolic Thioredoxins and Suppresses Plant Immunity. *mBio* **2016**, *7*, e00359-00316, doi:10.1128/mBio.00359-16.
231. Ji, C.Y.; Kim, Y.H.; Kim, H.S.; Ke, Q.; Kim, G.W.; Park, S.C.; Lee, H.S.; Jeong, J.C.; Kwak, S.S. Molecular characterization of tocopherol biosynthetic genes in sweetpotato that respond to stress and activate the tocopherol production in tobacco. *Plant Physiol Biochem* **2016**, *106*, 118-128, doi:10.1016/j.plaphy.2016.04.037.
232. Liu, M.; Peng, Y.; Li, H.; Deng, L.; Wang, X.; Kang, Z. TaSYP71, a Qc-SNARE, Contributes to Wheat Resistance against Puccinia striiformis f. sp. tritici. *Front Plant Sci* **2016**, *7*, 544, doi:10.3389/fpls.2016.00544.
233. Xu, R.X.; Gao, S.; Zhao, Y.; Lou, H.X.; Cheng, A.X. Functional characterization of a Mg(2+)-dependent O-methyltransferase with coumarin as preferred substrate from the liverwort Plagiochasma appendiculatum. *Plant Physiol Biochem* **2016**, *106*, 269-277, doi:10.1016/j.plaphy.2016.05.018.
234. Ono, N.N.; Qin, X.; Wilson, A.E.; Li, G.; Tian, L. Two UGT84 Family Glycosyltransferases Catalyze a Critical Reaction of Hydrolyzable Tannin Biosynthesis in Pomegranate (Punica granatum). *PLoS One* **2016**, *11*, e0156319, doi:10.1371/journal.pone.0156319.
235. Xiang, J.; Li, X.; Wu, J.; Yin, L.; Zhang, Y.; Lu, J. Studying the Mechanism of Plasmopara viticola RxLR Effectors on Suppressing Plant Immunity. *Front Microbiol* **2016**, *7*, 709, doi:10.3389/fmicb.2016.00709.
236. De Oliveira, A.S.; Koolhaas, I.; Boiteux, L.S.; Caldararu, O.F.; Petrescu, A.J.; Oliveira Resende, R.; Kormelink, R. Cell death triggering and effector recognition by Sw-5 SD-CNL proteins from resistant and susceptible tomato isolines to Tomato spotted wilt virus. *Mol Plant Pathol* **2016**, *17*, 1442-1454, doi:10.1111/mpp.12439.
237. Villarroel, C.A.; Jonckheere, W.; Alba, J.M.; Glas, J.J.; Dermauw, W.; Haring, M.A.; Van Leeuwen, T.; Schuurink, R.C.; Kant, M.R. Salivary proteins of spider mites suppress defenses in Nicotiana benthamiana and promote mite reproduction. *Plant J* **2016**, *86*, 119-131, doi:10.1111/tpj.13152.
238. Lai, B.; Du, L.N.; Liu, R.; Hu, B.; Su, W.B.; Qin, Y.H.; Zhao, J.T.; Wang, H.C.; Hu, G.B. Two LcbHLH Transcription Factors Interacting with LcMYB1 in Regulating Late Structural Genes of Anthocyanin Biosynthesis in Nicotiana and Litchi chinensis During Anthocyanin Accumulation. *Front Plant Sci* **2016**, *7*, 166, doi:10.3389/fpls.2016.00166.
239. Hamel, L.P.; Sekine, K.T.; Wallon, T.; Sugiwaka, Y.; Kobayashi, K.; Moffett, P. The Chloroplastic Protein THF1 Interacts with the Coiled-Coil Domain of the Disease Resistance Protein N' and Regulates Light-Dependent Cell Death. *Plant Physiol* **2016**, *171*, 658-674, doi:10.1104/pp.16.00234.
240. Yang, L.; McLellan, H.; Naqvi, S.; He, Q.; Boevink, P.C.; Armstrong, M.; Giuliani, L.M.; Zhang, W.; Tian, Z.; Zhan, J., et al. Potato NPH3/RPT2-Like Protein StNRL1, Targeted by a Phytophthora infestans RXLR Effector, Is a Susceptibility Factor. *Plant Physiol* **2016**, *171*, 645-657, doi:10.1104/pp.16.00178.
241. Rolland, V.; Badger, M.R.; Price, G.D. Redirecting the Cyanobacterial Bicarbonate Transporters BicA and SbtA to the Chloroplast Envelope: Soluble and Membrane Cargos Need Different Chloroplast Targeting Signals in Plants. *Front Plant Sci* **2016**, *7*, 185, doi:10.3389/fpls.2016.00185.
242. Zhang, Y.; Dong, J. Imaging Spatial Reorganization of a MAPK Signaling Pathway Using the Tobacco Transient Expression System. *J Vis Exp* **2016**, 10.3791/53790, doi:10.3791/53790.

243. Ruge, H.; Flosdorff, S.; Ebersberger, I.; Chigri, F.; Vothknecht, U.C. The calmodulin-like proteins AtCML4 and AtCML5 are single-pass membrane proteins targeted to the endomembrane system by an N-terminal signal anchor sequence. *J Exp Bot* **2016**, *67*, 3985-3996, doi:10.1093/jxb/erw101.
244. Han, Z.; Yu, H.; Zhao, Z.; Hunter, D.; Luo, X.; Duan, J.; Tian, L. AtHD2D Gene Plays a Role in Plant Growth, Development, and Response to Abiotic Stresses in Arabidopsis thaliana. *Front Plant Sci* **2016**, *7*, 310, doi:10.3389/fpls.2016.00310.
245. Wieczorek, P.; Obrepalska-Steplowska, A. The N-terminal fragment of the tomato torrado virus RNA1-encoded polyprotein induces a hypersensitive response (HR)-like reaction in Nicotiana benthamiana. *Arch Virol* **2016**, *161*, 1849-1858, doi:10.1007/s00705-016-2841-8.
246. Matic, S.; Pegoraro, M.; Noris, E. The C2 protein of tomato yellow leaf curl Sardinia virus acts as a pathogenicity determinant and a 16-amino acid domain is responsible for inducing a hypersensitive response in plants. *Virus Res* **2016**, *215*, 12-19, doi:10.1016/j.virusres.2016.01.014.
247. Zhuo, K.; Chen, J.; Lin, B.; Wang, J.; Sun, F.; Hu, L.; Liao, J. A novel Meloidogyne enterolobii effector MeTCTP promotes parasitism by suppressing programmed cell death in host plants. *Mol Plant Pathol* **2017**, *18*, 45-54, doi:10.1111/mpp.12374.
248. Niu, J.; Liu, P.; Liu, Q.; Chen, C.; Guo, Q.; Yin, J.; Yang, G.; Jian, H. Msp40 effector of root-knot nematode manipulates plant immunity to facilitate parasitism. *Sci Rep* **2016**, *6*, 19443, doi:10.1038/srep19443.
249. Miao, M.; Niu, X.; Kud, J.; Du, X.; Avila, J.; Devarenne, T.P.; Kuhl, J.C.; Liu, Y.; Xiao, F. The ubiquitin ligase SEVEN IN ABSENTIA (SINA) ubiquitinates a defense-related NAC transcription factor and is involved in defense signaling. *New Phytol* **2016**, *211*, 138-148, doi:10.1111/nph.13890.
250. Crocoll, C.; Mirza, N.; Reichelt, M.; Gershenzon, J.; Halkier, B.A. Optimization of Engineered Production of the Glucoraphanin Precursor Dihomomethionine in Nicotiana benthamiana. *Front Bioeng Biotechnol* **2016**, *4*, 14, doi:10.3389/fbioe.2016.00014.
251. Lim, S.H.; Song, J.H.; Kim, D.H.; Kim, J.K.; Lee, J.Y.; Kim, Y.M.; Ha, S.H. Activation of anthocyanin biosynthesis by expression of the radish R2R3-MYB transcription factor gene RsMYB1. *Plant Cell Rep* **2016**, *35*, 641-653, doi:10.1007/s00299-015-1909-3.
252. Pitzschke, A.; Xue, H.; Persak, H.; Datta, S.; Seifert, G.J. Post-Translational Modification and Secretion of Azelaic Acid Induced 1 (AZI1), a Hybrid Proline-Rich Protein from Arabidopsis. *Int J Mol Sci* **2016**, *17*, doi:10.3390/ijms17010085.
253. Liehr, T.; Schreyer, I.; Kuechler, A.; Manolakos, E.; Singer, S.; Dufke, A.; Wilhelm, K.; Jancuskova, T.; Cmejla, R.; Othman, M.A.K., et al. Parental origin of deletions and duplications - about the necessity to check for cryptic inversions. *Mol Cytogenet* **2018**, *11*, 20, doi:10.1186/s13039-018-0369-1.
254. Rodriguez-Medina, C.; Boissinot, S.; Chapuis, S.; Gereige, D.; Rastegar, M.; Erdinger, M.; Revers, F.; Ziegler-Graff, V.; Brault, V. A protein kinase binds the C-terminal domain of the readthrough protein of Turnip yellows virus and regulates virus accumulation. *Virology* **2015**, *486*, 44-53, doi:10.1016/j.virol.2015.08.031.
255. Kovalskaya, N.; Foster-Frey, J.; Donovan, D.M.; Bauchan, G.; Hammond, R.W. Antimicrobial Activity of Bacteriophage Endolysin Produced in Nicotiana benthamiana Plants. *J Microbiol Biotechnol* **2016**, *26*, 160-170, doi:10.4014/jmb.1505.05060.
256. Pascual, M.B.; Canovas, F.M.; Avila, C. The NAC transcription factor family in maritime pine (Pinus Pinaster): molecular regulation of two genes involved in stress responses. *BMC Plant Biol* **2015**, *15*, 254, doi:10.1186/s12870-015-0640-0.
257. Nakano, M.; Yoshioka, H.; Ohnishi, K.; Hikichi, Y.; Kiba, A. Cell death-inducing stresses are required for defense activation in DS1-phosphatidic acid phosphatase-silenced Nicotiana benthamiana. *J Plant Physiol* **2015**, *184*, 15-19, doi:10.1016/j.jplph.2015.06.007.
258. Roy, S.; Banerjee, V.; Das, K.P. Understanding the Physical and Molecular Basis of Stability of Arabidopsis DNA Pol lambda under UV-B and High NaCl Stress. *PLoS One* **2015**, *10*, e0133843, doi:10.1371/journal.pone.0133843.

259. Xu, R.X.; Zhao, Y.; Gao, S.; Zhang, Y.Y.; Li, D.D.; Lou, H.X.; Cheng, A.X. Functional characterization of a plastidal cation-dependent O-methyltransferase from the liverwort Plagiochasma appendiculatum. *Phytochemistry* **2015**, *118*, 33-41, doi:10.1016/j.phytochem.2015.08.002.
260. Denkovskiene, E.; Paskevicius, S.; Werner, S.; Gleba, Y.; Razanskiene, A. Inducible Expression of Agrobacterium Virulence Gene VirE2 for Stringent Regulation of T-DNA Transfer in Plant Transient Expression Systems. *Mol Plant Microbe Interact* **2015**, *28*, 1247-1255, doi:10.1094/MPMI-05-15-0102-R.
261. Zhang, C.; Liu, X.J.; Wu, K.C.; Zheng, L.P.; Ding, Z.M.; Li, F.; Zou, P.; Yang, L.; Wu, J.G.; Wu, Z.J. Rice grassy stunt virus nonstructural protein p5 serves as a viral suppressor of RNA silencing and interacts with nonstructural protein p3. *Arch Virol* **2015**, *160*, 2769-2779, doi:10.1007/s00705-015-2560-6.
262. Bell, A.; Moreau, C.; Chinoy, C.; Spanner, R.; Dalmais, M.; Le Signor, C.; Bendahmane, A.; Klenell, M.; Domoney, C. SGRL can regulate chlorophyll metabolism and contributes to normal plant growth and development in Pisum sativum L. *Plant Mol Biol* **2015**, *89*, 539-558, doi:10.1007/s11103-015-0372-4.
263. Distefano, A.M.; Valinas, M.A.; Scuffi, D.; Lamattina, L.; Ten Have, A.; Garcia-Mata, C.; Laxalt, A.M. Phospholipase D delta knock-out mutants are tolerant to severe drought stress. *Plant Signal Behav* **2015**, *10*, e1089371, doi:10.1080/15592324.2015.1089371.
264. Yu, H.Q.; Yong, T.M.; Li, H.J.; Liu, Y.P.; Zhou, S.F.; Fu, F.L.; Li, W.C. Overexpression of a phospholipase Dalpha gene from Ammopiptanthus nanus enhances salt tolerance of phospholipase Dalpha1-deficient Arabidopsis mutant. *Planta* **2015**, *242*, 1495-1509, doi:10.1007/s00425-015-2390-5.
265. Jain, M.; Fleites, L.A.; Gabriel, D.W. Prophage-Encoded Peroxidase in 'Candidatus Liberibacter asiaticus' Is a Secreted Effector That Suppresses Plant Defenses. *Mol Plant Microbe Interact* **2015**, *28*, 1330-1337, doi:10.1094/MPMI-07-15-0145-R.
266. Chen, X.R.; Li, Y.P.; Li, Q.Y.; Xing, Y.P.; Liu, B.B.; Tong, Y.H.; Xu, J.Y. SCR96, a small cysteine-rich secretory protein of Phytophthora cactorum, can trigger cell death in the Solanaceae and is important for pathogenicity and oxidative stress tolerance. *Mol Plant Pathol* **2016**, *17*, 577-587, doi:10.1111/mpp.12303.
267. Meng, Y.; Zhang, Q.; Zhang, M.; Gu, B.; Huang, G.; Wang, Q.; Shan, W. The protein disulfide isomerase 1 of Phytophthora parasitica (PpPDI1) is associated with the haustoria-like structures and contributes to plant infection. *Front Plant Sci* **2015**, *6*, 632, doi:10.3389/fpls.2015.00632.
268. Dong, L.; Jongedijk, E.; Bouwmeester, H.; Van Der Krol, A. Monoterpene biosynthesis potential of plant subcellular compartments. *New Phytol* **2016**, *209*, 679-690, doi:10.1111/nph.13629.
269. Tian, X.; Wang, Z.; Li, X.; Lv, T.; Liu, H.; Wang, L.; Niu, H.; Bu, Q. Characterization and Functional Analysis of Pyrabactin Resistance-Like Abscisic Acid Receptor Family in Rice. *Rice (N Y)* **2015**, *8*, 28, doi:10.1186/s12284-015-0061-6.
270. Cai, Y.; Goodman, J.M.; Pyc, M.; Mullen, R.T.; Dyer, J.M.; Chapman, K.D. Arabidopsis SEIPIN Proteins Modulate Triacylglycerol Accumulation and Influence Lipid Droplet Proliferation. *Plant Cell* **2015**, *27*, 2616-2636, doi:10.1105/tpc.15.00588.
271. Wang, Y.; Cordewener, J.H.; America, A.H.; Shan, W.; Bouwmeester, K.; Govers, F. Arabidopsis Lectin Receptor Kinases LecRK-IX.1 and LecRK-IX.2 Are Functional Analogs in Regulating Phytophthora Resistance and Plant Cell Death. *Mol Plant Microbe Interact* **2015**, *28*, 1032-1048, doi:10.1094/MPMI-02-15-0025-R.
272. Mafurah, J.J.; Ma, H.; Zhang, M.; Xu, J.; He, F.; Ye, T.; Shen, D.; Chen, Y.; Rajput, N.A.; Dou, D. A Virulence Essential CRN Effector of Phytophthora capsici Suppresses Host Defense and Induces Cell Death in Plant Nucleus. *PLoS One* **2015**, *10*, e0127965, doi:10.1371/journal.pone.0127965.
273. Guo, T.W.; Vimalesvaran, D.; Thompson, J.R.; Perry, K.L.; Krenz, B. Subcellular localization of grapevine red blotch-associated virus ORFs V2 and V3. *Virus Genes* **2015**, *51*, 156-158, doi:10.1007/s11262-015-1205-x.
274. Li, D.; Zhang, H.; Song, Q.; Wang, L.; Liu, S.; Hong, Y.; Huang, L.; Song, F. Tomato Sl3-MMP, a member of the Matrix metalloproteinase family, is required for disease resistance against Botrytis cinerea and Pseudomonas syringae pv. tomato DC3000. *BMC Plant Biol* **2015**, *15*, 143, doi:10.1186/s12870-015-0536-z.

275. Blondeau, K.; Blaise, F.; Graille, M.; Kale, S.D.; Linglin, J.; Ollivier, B.; Labarde, A.; Lazar, N.; Daverdin, G.; Balesdent, M.H., et al. Crystal structure of the effector AvrLm4-7 of Leptosphaeria maculans reveals insights into its translocation into plant cells and recognition by resistance proteins. *Plant J* **2015**, *83*, 610-624, doi:10.1111/tpj.12913.
276. Mou, S.; Shi, L.; Lin, W.; Liu, Y.; Shen, L.; Guan, D.; He, S. Over-Expression of Rice CBS Domain Containing Protein, OsCBSX3, Confers Rice Resistance to Magnaporthe oryzae Inoculation. *Int J Mol Sci* **2015**, *16*, 15903-15917, doi:10.3390/ijms160715903.
277. Bouton, C.; Geldreich, A.; Ramel, L.; Ryabova, L.A.; Dimitrova, M.; Keller, M. Cauliflower mosaic virus Transcriptome Reveals a Complex Alternative Splicing Pattern. *PLoS One* **2015**, *10*, e0132665, doi:10.1371/journal.pone.0132665.
278. Manosalva, P.; Manohar, M.; Kogel, K.H.; Kang, H.G.; Klessig, D.F. The GHKL ATPase MORC1 Modulates Species-Specific Plant Immunity in Solanaceae. *Mol Plant Microbe Interact* **2015**, *28*, 927-942, doi:10.1094/MPMI-12-14-0401-R.
279. Chi, Y.; Yang, Y.; Li, G.; Wang, F.; Fan, B.; Chen, Z. Identification and characterization of a novel group of legume-specific, Golgi apparatus-localized WRKY and Exo70 proteins from soybean. *J Exp Bot* **2015**, *66*, 3055-3070, doi:10.1093/jxb/erv104.
280. Singh, P.; Savithri, H.S. GBNV encoded movement protein (NSm) remodels ER network via C-terminal coiled coil domain. *Virology* **2015**, *482*, 133-146, doi:10.1016/j.virol.2015.01.030.
281. Chen, C.; Liu, S.; Liu, Q.; Niu, J.; Liu, P.; Zhao, J.; Jian, H. An ANNEXIN-like protein from the cereal cyst nematode Heterodera avenae suppresses plant defense. *PLoS One* **2015**, *10*, e0122256, doi:10.1371/journal.pone.0122256.
282. Matic, S.; Quaglino, E.; Arata, L.; Riccardo, F.; Pegoraro, M.; Vallino, M.; Cavallo, F.; Noris, E. The rat ErbB2 tyrosine kinase receptor produced in plants is immunogenic in mice and confers protective immunity against ErbB2(+) mammary cancer. *Plant Biotechnol J* **2016**, *14*, 153-159, doi:10.1111/pbi.12367.
283. Yan, J.; Aboshi, T.; Teraishi, M.; Strickler, S.R.; Spindel, J.E.; Tung, C.W.; Takata, R.; Matsumoto, F.; Maesaka, Y.; McCouch, S.R., et al. The Tyrosine Aminomutase TAM1 Is Required for beta-Tyrosine Biosynthesis in Rice. *Plant Cell* **2015**, *27*, 1265-1278, doi:10.1105/tpc.15.00058.
284. Kung, Y.J.; You, B.J.; Raja, J.A.; Chen, K.C.; Huang, C.H.; Bau, H.J.; Yang, C.F.; Huang, C.H.; Chang, C.P.; Yeh, S.D. Nucleotide sequence-homology-independent breakdown of transgenic resistance by more virulent virus strains and a potential solution. *Sci Rep* **2015**, *5*, 9804, doi:10.1038/srep09804.
285. Jutras, P.V.; D'Aoust, M.A.; Couture, M.M.; Vezina, L.P.; Goulet, M.C.; Michaud, D.; Sainsbury, F. Modulating secretory pathway pH by proton channel co-expression can increase recombinant protein stability in plants. *Biotechnol J* **2015**, *10*, 1478-1486, doi:10.1002/biot.201500056.
286. Cavallini, E.; Matus, J.T.; Finezzo, L.; Zenoni, S.; Loyola, R.; Guzzo, F.; Schlechter, R.; Ageorges, A.; Arce-Johnson, P.; Tornielli, G.B. The phenylpropanoid pathway is controlled at different branches by a set of R2R3-MYB C2 repressors in grapevine. *Plant Physiol* **2015**, *167*, 1448-1470, doi:10.1104/pp.114.256172.
287. Li, X.J.; Li, M.; Zhou, Y.; Hu, S.; Hu, R.; Chen, Y.; Li, X.B. Overexpression of cotton RAV1 gene in Arabidopsis confers transgenic plants high salinity and drought sensitivity. *PLoS One* **2015**, *10*, e0118056, doi:10.1371/journal.pone.0118056.
288. Lim, C.W.; Baek, W.; Lim, S.; Han, S.W.; Lee, S.C. Expression and Functional Roles of the Pepper Pathogen-Induced bZIP Transcription Factor CabZIP2 in Enhanced Disease Resistance to Bacterial Pathogen Infection. *Mol Plant Microbe Interact* **2015**, *28*, 825-833, doi:10.1094/MPMI-10-14-0313-R.
289. Yu, H.Q.; Zhang, Y.Y.; Yong, T.M.; Liu, Y.P.; Zhou, S.F.; Fu, F.L.; Li, W.C. Cloning and functional validation of molybdenum cofactor sulfurase gene from Ammopiptanthus nanus. *Plant Cell Rep* **2015**, *34*, 1165-1176, doi:10.1007/s00299-015-1775-z.
290. Lin, Y.T.; Wei, H.M.; Lu, H.Y.; Lee, Y.I.; Fu, S.F. Developmental- and Tissue-Specific Expression of NbCMT3-2 Encoding a Chromomethylase in Nicotiana benthamiana. *Plant Cell Physiol* **2015**, *56*, 1124-1143, doi:10.1093/pcp/pcv036.

291. Luan, M.; Xu, M.; Lu, Y.; Zhang, L.; Fan, Y.; Wang, L. Expression of zma-miR169 miRNAs and their target ZmNF-YA genes in response to abiotic stress in maize leaves. *Gene* **2015**, *555*, 178-185, doi:10.1016/j.gene.2014.11.001.
292. Liu, H.; Chang, Q.; Feng, W.; Zhang, B.; Wu, T.; Li, N.; Yao, F.; Ding, X.; Chu, Z. Domain dissection of AvrRxo1 for suppressor, avirulence and cytotoxicity functions. *PLoS One* **2014**, *9*, e113875, doi:10.1371/journal.pone.0113875.
293. Yu, S.; Pilot, G. Testing the efficiency of plant artificial microRNAs by transient expression in Nicotiana benthamiana reveals additional action at the translational level. *Front Plant Sci* **2014**, *5*, 622, doi:10.3389/fpls.2014.00622.
294. Lin-Wang, K.; McGhie, T.K.; Wang, M.; Liu, Y.; Warren, B.; Storey, R.; Espley, R.V.; Allan, A.C. Engineering the anthocyanin regulatory complex of strawberry (Fragaria vesca). *Front Plant Sci* **2014**, *5*, 651, doi:10.3389/fpls.2014.00651.
295. Zhou, Y.; Zhou, H.; Lin-Wang, K.; Vimolmangkang, S.; Espley, R.V.; Wang, L.; Allan, A.C.; Han, Y. Transcriptome analysis and transient transformation suggest an ancient duplicated MYB transcription factor as a candidate gene for leaf red coloration in peach. *BMC Plant Biol* **2014**, *14*, 388, doi:10.1186/s12870-014-0388-y.
296. Buyel, J.F.; Buyel, J.J.; Haase, C.; Fischer, R. The impact of Pseudomonas syringae type III effectors on transient protein expression in tobacco. *Plant Biol (Stuttg)* **2015**, *17*, 484-492, doi:10.1111/plb.12264.
297. Feng, C.Z.; Chen, Y.; Wang, C.; Kong, Y.H.; Wu, W.H.; Chen, Y.F. Arabidopsis RAV1 transcription factor, phosphorylated by SnRK2 kinases, regulates the expression of ABI3, ABI4, and ABI5 during seed germination and early seedling development. *Plant J* **2014**, *80*, 654-668, doi:10.1111/tpj.12670.
298. Ruocco, M.; Lanzuise, S.; Lombardi, N.; Woo, S.L.; Vinale, F.; Marra, R.; Varlese, R.; Manganiello, G.; Pascale, A.; Scala, V., et al. Multiple roles and effects of a novel Trichoderma hydrophobin. *Mol Plant Microbe Interact* **2015**, *28*, 167-179, doi:10.1094/MPMI-07-14-0194-R.
299. Kim, D.S.; Kim, N.H.; Hwang, B.K. GLYCINE-RICH RNA-BINDING PROTEIN1 interacts with RECEPTOR-LIKE CYTOPLASMIC PROTEIN KINASE1 and suppresses cell death and defense responses in pepper (Capsicum annuum). *New Phytol* **2015**, *205*, 786-800, doi:10.1111/nph.13105.
300. Lund, C.H.; Bromley, J.R.; Stenbaek, A.; Rasmussen, R.E.; Scheller, H.V.; Sakuragi, Y. A reversible Renilla luciferase protein complementation assay for rapid identification of protein-protein interactions reveals the existence of an interaction network involved in xyloglucan biosynthesis in the plant Golgi apparatus. *J Exp Bot* **2015**, *66*, 85-97, doi:10.1093/jxb/eru401.
301. Zhang, M.; Li, Q.; Liu, T.; Liu, L.; Shen, D.; Zhu, Y.; Liu, P.; Zhou, J.M.; Dou, D. Two cytoplasmic effectors of Phytophthora sojae regulate plant cell death via interactions with plant catalases. *Plant Physiol* **2015**, *167*, 164-175, doi:10.1104/pp.114.252437.
302. Dafny-Yelin, M.; Levy, A.; Dafny, R.; Tzfira, T. Blocking single-stranded transferred DNA conversion to double-stranded intermediates by overexpression of yeast DNA REPLICATION FACTOR A. *Plant Physiol* **2015**, *167*, 153-163, doi:10.1104/pp.114.250639.
303. Li, X.; Zhang, Y.; Huang, L.; Ouyang, Z.; Hong, Y.; Zhang, H.; Li, D.; Song, F. Tomato SlMKK2 and SlMKK4 contribute to disease resistance against Botrytis cinerea. *BMC Plant Biol* **2014**, *14*, 166, doi:10.1186/1471-2229-14-166.
304. Gallage, N.J.; Hansen, E.H.; Kannangara, R.; Olsen, C.E.; Motawia, M.S.; Jorgensen, K.; Holme, I.; Hebelstrup, K.; Grisoni, M.; Moller, B.L. Vanillin formation from ferulic acid in Vanilla planifolia is catalysed by a single enzyme. *Nat Commun* **2014**, *5*, 4037, doi:10.1038/ncomms5037.
305. Zhang, W.; Kollwig, G.; Stecyk, E.; Apelt, F.; Dirks, R.; Kragler, F. Graft-transmissible movement of inverted-repeat-induced siRNA signals into flowers. *Plant J* **2014**, *80*, 106-121, doi:10.1111/tpj.12622.
306. Graumann, K.; Vanrobays, E.; Tutois, S.; Probst, A.V.; Evans, D.E.; Tatout, C. Characterization of two distinct subfamilies of SUN-domain proteins in Arabidopsis and their interactions with the novel KASH-domain protein AtTIK. *J Exp Bot* **2014**, *65*, 6499-6512, doi:10.1093/jxb/eru368.
307. Oh, T.K.; Oh, S.; Kim, S.; Park, J.S.; Vinod, N.; Jang, K.M.; Kim, S.C.; Choi, C.W.; Ko, S.M.; Jeong, D.K., et al. Expression of Aspergillus nidulans phy gene in Nicotiana benthamiana produces

active phytase with broad specificities. *Int J Mol Sci* **2014**, *15*, 15571-15591, doi:10.3390/ijms150915571.
308. Kiba, A.; Galis, I.; Hojo, Y.; Ohnishi, K.; Yoshioka, H.; Hikichi, Y. SEC14 phospholipid transfer protein is involved in lipid signaling-mediated plant immune responses in Nicotiana benthamiana. *PLoS One* **2014**, *9*, e98150, doi:10.1371/journal.pone.0098150.
309. Lee, H.A.; Kim, S.Y.; Oh, S.K.; Yeom, S.I.; Kim, S.B.; Kim, M.S.; Kamoun, S.; Choi, D. Multiple recognition of RXLR effectors is associated with nonhost resistance of pepper against Phytophthora infestans. *New Phytol* **2014**, *203*, 926-938, doi:10.1111/nph.12861.
310. Wu, W.; Cheng, Z.; Liu, M.; Yang, X.; Qiu, D. C3HC4-type RING finger protein NbZFP1 is involved in growth and fruit development in Nicotiana benthamiana. *PLoS One* **2014**, *9*, e99352, doi:10.1371/journal.pone.0099352.
311. Divi, U.K.; El Tahchy, A.; Vanhercke, T.; Petrie, J.R.; Robles-Martinez, J.A.; Singh, S.P. Transcriptional and biochemical responses of monoacylglycerol acyltransferase-mediated oil synthesis and associated senescence-like responses in Nicotiana benthamiana. *Front Plant Sci* **2014**, *5*, 204, doi:10.3389/fpls.2014.00204.
312. Meshcheriakova, Y.A.; Saxena, P.; Lomonossoff, G.P. Fine-tuning levels of heterologous gene expression in plants by orthogonal variation of the untranslated regions of a nonreplicating transient expression system. *Plant Biotechnol J* **2014**, *12*, 718-727, doi:10.1111/pbi.12175.
313. Senechal, F.; Graff, L.; Surcouf, O.; Marcelo, P.; Rayon, C.; Bouton, S.; Mareck, A.; Mouille, G.; Stintzi, A.; Hofte, H., et al. Arabidopsis PECTIN METHYLESTERASE17 is co-expressed with and processed by SBT3.5, a subtilisin-like serine protease. *Ann Bot* **2014**, *114*, 1161-1175, doi:10.1093/aob/mcu035.
314. Meng, W.; Hsiao, A.S.; Gao, C.; Jiang, L.; Chye, M.L. Subcellular localization of rice acyl-CoA-binding proteins (ACBPs) indicates that OsACBP6::GFP is targeted to the peroxisomes. *New Phytol* **2014**, *203*, 469-482, doi:10.1111/nph.12809.
315. Nahar, K.; Matsumoto, I.; Taguchi, F.; Inagaki, Y.; Yamamoto, M.; Toyoda, K.; Shiraishi, T.; Ichinose, Y.; Mukaihara, T. Ralstonia solanacearum type III secretion system effector Rip36 induces a hypersensitive response in the nonhost wild eggplant Solanum torvum. *Mol Plant Pathol* **2014**, *15*, 297-303, doi:10.1111/mpp.12079.
316. Zheng, X.; McLellan, H.; Fraiture, M.; Liu, X.; Boevink, P.C.; Gilroy, E.M.; Chen, Y.; Kandel, K.; Sessa, G.; Birch, P.R., et al. Functionally redundant RXLR effectors from Phytophthora infestans act at different steps to suppress early flg22-triggered immunity. *PLoS Pathog* **2014**, *10*, e1004057, doi:10.1371/journal.ppat.1004057.
317. Gao, Y.; Zan, X.L.; Wu, X.F.; Yao, L.; Chen, Y.L.; Jia, S.W.; Zhao, K.J. Identification of fungus-responsive cis-acting element in the promoter of Brassica juncea chitinase gene, BjCHI1. *Plant Sci* **2014**, *215-216*, 190-198, doi:10.1016/j.plantsci.2013.11.008.
318. Lay, F.T.; Poon, S.; McKenna, J.A.; Connelly, A.A.; Barbeta, B.L.; McGinness, B.S.; Fox, J.L.; Daly, N.L.; Craik, D.J.; Heath, R.L., et al. The C-terminal propeptide of a plant defensin confers cytoprotective and subcellular targeting functions. *BMC Plant Biol* **2014**, *14*, 41, doi:10.1186/1471-2229-14-41.
319. Zheng, C.K.; Wang, C.L.; Zhang, X.P.; Wang, F.J.; Qin, T.F.; Zhao, K.J. The last half-repeat of transcription activator-like effector (TALE) is dispensable and thereby TALE-based technology can be simplified. *Mol Plant Pathol* **2014**, *15*, 690-697, doi:10.1111/mpp.12125.
320. Sahana, N.; Kaur, H.; Jain, R.K.; Palukaitis, P.; Canto, T.; Praveen, S. The asparagine residue in the FRNK box of potyviral helper-component protease is critical for its small RNA binding and subcellular localization. *J Gen Virol* **2014**, *95*, 1167-1177, doi:10.1099/vir.0.060269-0.
321. Del Toro, F.; Tenllado, F.; Chung, B.N.; Canto, T. A procedure for the transient expression of genes by agroinfiltration above the permissive threshold to study temperature-sensitive processes in plant-pathogen interactions. *Mol Plant Pathol* **2014**, *15*, 848-857, doi:10.1111/mpp.12136.
322. Huang, Y.Y.; Shi, Y.; Lei, Y.; Li, Y.; Fan, J.; Xu, Y.J.; Ma, X.F.; Zhao, J.Q.; Xiao, S.; Wang, W.M. Functional identification of multiple nucleocytoplasmic trafficking signals in the broad-spectrum resistance protein RPW8.2. *Planta* **2014**, *239*, 455-468, doi:10.1007/s00425-013-1994-x.

323. Hu, S.; Dong, G.; Xu, J.; Su, Y.; Shi, Z.; Ye, W.; Li, Y.; Li, G.; Zhang, B.; Hu, J., et al. A point mutation in the zinc finger motif of RID1/EHD2/OsID1 protein leads to outstanding yield-related traits in japonica rice variety Wuyunjing 7. *Rice (N Y)* **2013**, *6*, 24, doi:10.1186/1939-8433-6-24.
324. Ginglinger, J.F.; Boachon, B.; Hofer, R.; Paetz, C.; Kollner, T.G.; Miesch, L.; Lugan, R.; Baltenweck, R.; Mutterer, J.; Ullmann, P., et al. Gene coexpression analysis reveals complex metabolism of the monoterpene alcohol linalool in Arabidopsis flowers. *Plant Cell* **2013**, *25*, 4640-4657, doi:10.1105/tpc.113.117382.
325. Li, Z.; Zhang, L.; Li, J.; Xu, X.; Yao, Q.; Wang, A. Isolation and functional characterization of the ShCBF1 gene encoding a CRT/DRE-binding factor from the wild tomato species Solanum habrochaites. *Plant Physiol Biochem* **2014**, *74*, 294-303, doi:10.1016/j.plaphy.2013.11.024.
326. Hwang, I.S.; Choi, D.S.; Kim, N.H.; Kim, D.S.; Hwang, B.K. Pathogenesis-related protein 4b interacts with leucine-rich repeat protein 1 to suppress PR4b-triggered cell death and defense response in pepper. *Plant J* **2014**, *77*, 521-533, doi:10.1111/tpj.12400.
327. Gawehns, F.; Houterman, P.M.; Ichou, F.A.; Michielse, C.B.; Hijdra, M.; Cornelissen, B.J.; Rep, M.; Takken, F.L. The Fusarium oxysporum effector Six6 contributes to virulence and suppresses I-2-mediated cell death. *Mol Plant Microbe Interact* **2014**, *27*, 336-348, doi:10.1094/MPMI-11-13-0330-R.
328. Bruckner, K.; Tissier, A. High-level diterpene production by transient expression in Nicotiana benthamiana. *Plant Methods* **2013**, *9*, 46, doi:10.1186/1746-4811-9-46.
329. Stirnweis, D.; Milani, S.D.; Jordan, T.; Keller, B.; Brunner, S. Substitutions of two amino acids in the nucleotide-binding site domain of a resistance protein enhance the hypersensitive response and enlarge the PM3F resistance spectrum in wheat. *Mol Plant Microbe Interact* **2014**, *27*, 265-276, doi:10.1094/MPMI-10-13-0297-FI.
330. Choi, H.W.; Kim, D.S.; Kim, N.H.; Jung, H.W.; Ham, J.H.; Hwang, B.K. Xanthomonas filamentous hemagglutinin-like protein Fha1 interacts with pepper hypersensitive-induced reaction protein CaHIR1 and functions as a virulence factor in host plants. *Mol Plant Microbe Interact* **2013**, *26*, 1441-1454, doi:10.1094/MPMI-07-13-0204-R.
331. Acosta-Maspons, A.; Sepulveda-Garcia, E.; Sanchez-Baldoquin, L.; Marrero-Gutierrez, J.; Pons, T.; Rocha-Sosa, M.; Gonzalez, L. Two aspartate residues at the putative p10 subunit of a type II metacaspase from Nicotiana tabacum L. may contribute to the substrate-binding pocket. *Planta* **2014**, *239*, 147-160, doi:10.1007/s00425-013-1975-0.
332. Hwang, I.S.; Choi, D.S.; Kim, N.H.; Kim, D.S.; Hwang, B.K. The pepper cysteine/histidine-rich DC1 domain protein CaDC1 binds both RNA and DNA and is required for plant cell death and defense response. *New Phytol* **2014**, *201*, 518-530, doi:10.1111/nph.12521.
333. Pitzschke, A. Tropaeolum tops tobacco - simple and efficient transgene expression in the order Brassicales. *PLoS One* **2013**, *8*, e73355, doi:10.1371/journal.pone.0073355.
334. Golan, G.; Betzer, R.; Wolf, S. Phloem-specific expression of a melon Aux/IAA in tomato plants alters auxin sensitivity and plant development. *Front Plant Sci* **2013**, *4*, 329, doi:10.3389/fpls.2013.00329.
335. Geisler, K.; Hughes, R.K.; Sainsbury, F.; Lomonossoff, G.P.; Rejzek, M.; Fairhurst, S.; Olsen, C.E.; Motawia, M.S.; Melton, R.E.; Hemmings, A.M., et al. Biochemical analysis of a multifunctional cytochrome P450 (CYP51) enzyme required for synthesis of antimicrobial triterpenes in plants. *Proc Natl Acad Sci U S A* **2013**, *110*, E3360-3367, doi:10.1073/pnas.1309157110.
336. McLellan, H.; Boevink, P.C.; Armstrong, M.R.; Pritchard, L.; Gomez, S.; Morales, J.; Whisson, S.C.; Beynon, J.L.; Birch, P.R. An RxLR effector from Phytophthora infestans prevents re-localisation of two plant NAC transcription factors from the endoplasmic reticulum to the nucleus. *PLoS Pathog* **2013**, *9*, e1003670, doi:10.1371/journal.ppat.1003670.
337. Chen, S.; Chronis, D.; Wang, X. The novel GrCEP12 peptide from the plant-parasitic nematode Globodera rostochiensis suppresses flg22-mediated PTI. *Plant Signal Behav* **2013**, *8*, doi:10.4161/psb.25359.
338. Gupta, M.; Yoshioka, H.; Ohnishi, K.; Mizumoto, H.; Hikichi, Y.; Kiba, A. A translationally controlled tumor protein negatively regulates the hypersensitive response in Nicotiana benthamiana. *Plant Cell Physiol* **2013**, *54*, 1403-1414, doi:10.1093/pcp/pct090.

339. Sugawara, K.; Honma, Y.; Komatsu, K.; Himeno, M.; Oshima, K.; Namba, S. The alteration of plant morphology by small peptides released from the proteolytic processing of the bacterial peptide TENGU. *Plant Physiol* **2013**, *162*, 2005-2014, doi:10.1104/pp.113.218586.
340. Zhu, F.Y.; Li, L.; Lam, P.Y.; Chen, M.X.; Chye, M.L.; Lo, C. Sorghum extracellular leucine-rich repeat protein SbLRR2 mediates lead tolerance in transgenic Arabidopsis. *Plant Cell Physiol* **2013**, *54*, 1549-1559, doi:10.1093/pcp/pct101.
341. Bamunusinghe, D.; Chaturvedi, S.; Seo, J.K.; Rao, A.L. Mutations in the capsid protein of Brome mosaic virus affecting encapsidation eliminate vesicle induction in planta: implications for virus cell-to-cell spread. *J Virol* **2013**, *87*, 8982-8992, doi:10.1128/JVI.01253-13.
342. Abdel-Hamid, H.; Chin, K.; Moeder, W.; Shahinas, D.; Gupta, D.; Yoshioka, K. A suppressor screen of the chimeric AtCNGC11/12 reveals residues important for intersubunit interactions of cyclic nucleotide-gated ion channels. *Plant Physiol* **2013**, *162*, 1681-1693, doi:10.1104/pp.113.217539.
343. Hoser, R.; Zurczak, M.; Lichocka, M.; Zuzga, S.; Dadlez, M.; Samuel, M.A.; Ellis, B.E.; Stuttmann, J.; Parker, J.E.; Hennig, J., et al. Nucleocytoplasmic partitioning of tobacco N receptor is modulated by SGT1. *New Phytol* **2013**, *200*, 158-171, doi:10.1111/nph.12347.
344. Fischer, M.J.; Meyer, S.; Claudel, P.; Perrin, M.; Ginglinger, J.F.; Gertz, C.; Masson, J.E.; Werck-Reinhardt, D.; Hugueney, P.; Karst, F. Specificity of Ocimum basilicum geraniol synthase modified by its expression in different heterologous systems. *J Biotechnol* **2013**, *163*, 24-29, doi:10.1016/j.jbiotec.2012.10.012.
345. Yin, W.; Dong, S.; Zhai, L.; Lin, Y.; Zheng, X.; Wang, Y. The Phytophthora sojae Avr1d gene encodes an RxLR-dEER effector with presence and absence polymorphisms among pathogen strains. *Mol Plant Microbe Interact* **2013**, *26*, 958-968, doi:10.1094/MPMI-02-13-0035-R.
346. Chronis, D.; Chen, S.; Lu, S.; Hewezi, T.; Carpenter, S.C.; Loria, R.; Baum, T.J.; Wang, X. A ubiquitin carboxyl extension protein secreted from a plant-parasitic nematode Globodera rostochiensis is cleaved in planta to promote plant parasitism. *Plant J* **2013**, *74*, 185-196, doi:10.1111/tpj.12125.
347. Giska, F.; Lichocka, M.; Piechocki, M.; Dadlez, M.; Schmelzer, E.; Hennig, J.; Krzymowska, M. Phosphorylation of HopQ1, a type III effector from Pseudomonas syringae, creates a binding site for host 14-3-3 proteins. *Plant Physiol* **2013**, *161*, 2049-2061, doi:10.1104/pp.112.209023.
348. Cao, S.; Zhou, X.R.; Wood, C.C.; Green, A.G.; Singh, S.P.; Liu, L.; Liu, Q. A large and functionally diverse family of Fad2 genes in safflower (Carthamus tinctorius L.). *BMC Plant Biol* **2013**, *13*, 5, doi:10.1186/1471-2229-13-5.
349. Zhang, H.; Liu, Y.; Xu, Y.; Chapman, S.; Love, A.J.; Xia, T. A newly isolated Na+/H+ antiporter gene, DmNHX1, confers salt tolerance when expressed transiently in Nicotiana benthamiana or stably in Arabidopsis thaliana. *Plant Cell, Tissue and Organ Culture (PCTOC)* **2012**, *110*, 189-200, doi:10.1007/s11240-012-0142-9.
350. Hernández, M.L.; Whitehead, L.; He, Z.; Gazda, V.; Gilday, A.; Kozhevnikova, E.; Vaistij, F.E.; Larson, T.R.; Graham, I.A. A Cytosolic Acyltransferase Contributes to Triacylglycerol Synthesis in Sucrose-Rescued Arabidopsis Seed Oil Catabolism Mutants. *Plant Physiology* **2012**, *160*, 215-225, doi:10.1104/pp.112.201541.
351. Kobayashi, M.; Yamamoto-Katou, A.; Katou, S.; Hirai, K.; Meshi, T.; Ohashi, Y.; Mitsuhara, I. Identification of an amino acid residue required for differential recognition of a viral movement protein by the Tomato mosaic virus resistance gene Tm-22. *Journal of Plant Physiology* **2011**, *168*, 1142-1145, doi:https://doi.org/10.1016/j.jplph.2011.01.002.
352. Au - Kabbage, M.; Au - Ek-Ramos, M.; Au - Dickman, M. A β-glucuronidase (GUS) Based Cell Death Assay

JoVE **2011**, doi:10.3791/2680, e2680, doi:doi:10.3791/2680.
353. Matousek, J.; Kocabek, T.; Patzak, J.; Stehlik, J.; Fussy, Z.; Krofta, K.; Heyerick, A.; Roldan-Ruiz, I.; Maloukh, L.; De Keukeleire, D. Cloning and molecular analysis of HlbZip1 and HlbZip2 transcription factors putatively involved in the regulation of the lupulin metabolome in hop (Humulus lupulus L.). *J Agric Food Chem* **2010**, *58*, 902-912, doi:10.1021/jf9043106.
354. Ma, P.; Liu, J.; He, H.; Yang, M.; Li, M.; Zhu, X.; Wang, X. A viral suppressor P1/HC-pro increases the GFP gene expression in agrobacterium-mediated transient assay. *Appl Biochem Biotechnol* **2009**, *158*, 243-252, doi:10.1007/s12010-008-8332-y.

355. Senshu, H.; Ozeki, J.; Komatsu, K.; Hashimoto, M.; Hatada, K.; Aoyama, M.; Kagiwada, S.; Yamaji, Y.; Namba, S. Variability in the level of RNA silencing suppression caused by triple gene block protein 1 (TGBp1) from various potexviruses during infection. *J Gen Virol* **2009**, *90*, 1014-1024, doi:10.1099/vir.0.008243-0.
356. Condori, J.; Medrano, G.; Sivakumar, G.; Nair, V.; Cramer, C.; Medina-Bolivar, F. Functional characterization of a stilbene synthase gene using a transient expression system in planta. *Plant Cell Rep* **2009**, *28*, 589-599, doi:10.1007/s00299-008-0664-0.
357. Gurlebeck, D.; Jahn, S.; Gurlebeck, N.; Szczesny, R.; Szurek, B.; Hahn, S.; Hause, G.; Bonas, U. Visualization of novel virulence activities of the Xanthomonas type III effectors AvrBs1, AvrBs3 and AvrBs4. *Mol Plant Pathol* **2009**, *10*, 175-188, doi:10.1111/j.1364-3703.2008.00519.x.
358. Vargas, M.; Martinez-Garcia, B.; Diaz-Ruiz, J.R.; Tenllado, F. Transient expression of homologous hairpin RNA interferes with PVY transmission by aphids. *Virol J* **2008**, *5*, 42, doi:10.1186/1743-422X-5-42.
359. Hawes, C.; Brandizzil, F.; Batoko, H.; Moore, I. Organelle motility in plant cells: imaging golgi and ER dynamics with GFP. *Curr Protoc Cell Biol* **2001**, *Chapter 13*, Unit 13 13, doi:10.1002/0471143030.cb1303s09.
360. Tai, T.H.; Dahlbeck, D.; Clark, E.T.; Gajiwala, P.; Pasion, R.; Whalen, M.C.; Stall, R.E.; Staskawicz, B.J. Expression of the Bs2 pepper gene confers resistance to bacterial spot disease in tomato. *Proc Natl Acad Sci U S A* **1999**, *96*, 14153-14158, doi:10.1073/pnas.96.24.14153.
361. Chen, H.M.; Chen, L.T.; Patel, K.; Li, Y.H.; Baulcombe, D.C.; Wu, S.H. 22-Nucleotide RNAs trigger secondary siRNA biogenesis in plants. *Proc Natl Acad Sci U S A* **2010**, *107*, 15269-15274, doi:10.1073/pnas.1001738107.
362. Kanagarajan, S.; Muthusamy, S.; Gliszczynska, A.; Lundgren, A.; Brodelius, P.E. Functional expression and characterization of sesquiterpene synthases from Artemisia annua L. using transient expression system in Nicotiana benthamiana. *Plant Cell Rep* **2012**, *31*, 1309-1319, doi:10.1007/s00299-012-1250-z.
363. Yuasa, T.; Sugiki, M.; Watanabe, Y. Activation of SIPK in response to UV-C irradiation: utility of a glutathione-S transferase–tagged plant MAP kinase by transient expression with agroinfiltration. *Plant Biotechnology* **2005**, *22*, 7-12, doi:10.5511/plantbiotechnology.22.7.
364. Renovell, Á.; Vives, M.C.; Ruiz-Ruiz, S.; Navarro, L.; Moreno, P.; Guerri, J. The Citrus leaf blotch virus movement protein acts as silencing suppressor. *Virus Genes* **2012**, *44*, 131-140, doi:10.1007/s11262-011-0674-9.
365. Ogata, T.; Kida, Y.; Arai, T.; Kishi, Y.; Manago, Y.; Murai, M.; Matsushita, Y. Overexpression of tobacco ethylene response factor NtERF3 gene and its homologues from tobacco and rice induces hypersensitive response-like cell death in tobacco. *Journal of General Plant Pathology* **2012**, *78*, 8-17, doi:10.1007/s10327-011-0355-5.
366. Ghazala, W.; Waltermann, A.; Pilot, R.; Winter, S.; Varrelmann, M. Functional characterization and subcellular localization of the 16K cysteine-rich suppressor of gene silencing protein of tobacco rattle virus. *J Gen Virol* **2008**, *89*, 1748-1758, doi:10.1099/vir.0.83503-0.
367. Son, O.; Cho, S.K.; Kim, S.J.; Kim, W.T. In vitro and in vivo interaction of AtRma2 E3 ubiquitin ligase and auxin binding protein 1. *Biochem Biophys Res Commun* **2010**, *393*, 492-497, doi:10.1016/j.bbrc.2010.02.032.
368. Ma, L.; Lukasik, E.; Gawehns, F.; Takken, F.L. The use of agroinfiltration for transient expression of plant resistance and fungal effector proteins in Nicotiana benthamiana leaves. *Methods Mol Biol* **2012**, *835*, 61-74, doi:10.1007/978-1-61779-501-5_4.
369. Hori, K.; Watanabe, Y. Context analysis of termination codons in mRNA that are recognized by plant NMD. *Plant Cell Physiol* **2007**, *48*, 1072-1078, doi:10.1093/pcp/pcm075.
370. Jacques, A.; Ghannam, A.; Erhardt, M.; de Ruffray, P.; Baillieul, F.; Kauffmann, S. NtLRP1, a tobacco leucine-rich repeat gene with a possible role as a modulator of the hypersensitive response. *Mol Plant Microbe Interact* **2006**, *19*, 747-757, doi:10.1094/MPMI-19-0747.
371. Joensuu, J.J.; Conley, A.J.; Lienemann, M.; Brandle, J.E.; Linder, M.B.; Menassa, R. Hydrophobin fusions for high-level transient protein expression and purification in Nicotiana benthamiana. *Plant Physiol* **2010**, *152*, 622-633, doi:10.1104/pp.109.149021.

372. Seong, E.S.; Kwon, S.S.; Ghimire, B.K.; Yu, C.Y.; Cho, D.H.; Lim, J.D.; Kim, K.S.; Heo, K.; Lim, E.S.; Chung, I.M., et al. LebZIP2 induced by salt and drought stress and transient overexpression by Agrobacterium. *BMB Rep* **2008**, *41*, 693-698, doi:10.5483/bmbrep.2008.41.10.693.
373. Kataya, A.R.; Suliman, M.N.; Kalantidis, K.; Livieratos, I.C. Cucurbit yellow stunting disorder virus p25 is a suppressor of post-transcriptional gene silencing. *Virus Res* **2009**, *145*, 48-53, doi:10.1016/j.virusres.2009.06.010.
374. Yoon, J.; Chung, W.I.; Choi, D. NbHB1, Nicotiana benthamiana homeobox 1, is a jasmonic acid-dependent positive regulator of pathogen-induced plant cell death. *New Phytol* **2009**, *184*, 71-84, doi:10.1111/j.1469-8137.2009.02967.x.
375. Takahashi, S.; Komatsu, K.; Kagiwada, S.; Ozeki, J.; Mori, T.; Hirata, H.; Yamaji, Y.; Ugaki, M.; Namba, S. The efficiency of interference of Potato virus X infection depends on the target gene. *Virus Res* **2006**, *116*, 214-217, doi:10.1016/j.virusres.2005.11.002.
376. Sindarovska, Y.R.; Gerasymenko, I.M.; Kuchuk, M.V.; Sheludko, Y.V. Influence of exogenous phytohormones, methyl jasmonate and suppressors of jasmonate biosynthesis on Agrobacterium-mediated transient expression in Nicotiana excelsior. *Biopolymers and Cell* **2012**, *28*, 368-373, doi:10.7124/bc.000072.
377. Yu, Y.; Guo, D.; Li, G.; Yang, Y.; Zhang, G.; Li, S.; Liang, Z. The grapevine R2R3-type MYB transcription factor VdMYB1 positively regulates defense responses by activating the stilbene synthase gene 2 (VdSTS2). *BMC Plant Biology* **2019**, *19*, 478, doi:10.1186/s12870-019-1993-6.
378. Tounsi, S.; Kamoun, Y.; Feki, K.; Jemli, S.; Saidi, M.N.; Ziadi, H.; Alcon, C.; Brini, F. Localization and expression analysis of a novel catalase from Triticum monococcum TmCAT1 involved in response to different environmental stresses. *Plant Physiol Biochem* **2019**, *139*, 366-378, doi:10.1016/j.plaphy.2019.03.039.
379. Deb, D.; Dey, N. Synthetic Salicylic acid inducible recombinant promoter for translational research. *J Biotechnol* **2019**, *297*, 9-18, doi:10.1016/j.jbiotec.2019.03.004.
380. Bidarigh Fard, A.; Dehghan Nayeri, F.; Habibi Anbuhi, M. Transient expression of etanercept therapeutic protein in tobacco (Nicotiana tabacum L.). *Int J Biol Macromol* **2019**, *130*, 483-490, doi:10.1016/j.ijbiomac.2019.02.153.
381. Barabasz, A.; Palusinska, M.; Papierniak, A.; Kendziorek, M.; Kozak, K.; Williams, L.E.; Antosiewicz, D.M. Functional Analysis of NtZIP4B and Zn Status-Dependent Expression Pattern of Tobacco ZIP Genes. *Front Plant Sci* **2018**, *9*, 1984, doi:10.3389/fpls.2018.01984.
382. Hua-Ying, M.; Wen-Ju, W.; Wei-Hua, S.; Ya-Chun, S.; Feng, L.; Cong-Na, L.; Ling, W.; Xu, Z.; Li-Ping, X.; You-Xiong, Q. Genome-wide identification, phylogeny, and expression analysis of Sec14-like PITP gene family in sugarcane. *Plant Cell Rep* **2019**, *38*, 637-655, doi:10.1007/s00299-019-02394-1.
383. Wang, G.; Wang, P.; Gao, Y.; Li, Y.; Wu, L.; Gao, J.; Zhao, M.; Xia, Q. Isolation and functional characterization of a novel FLOWERING LOCUS T homolog (NtFT5) in Nicotiana tabacum. *J Plant Physiol* **2018**, *231*, 393-401, doi:10.1016/j.jplph.2018.10.021.
384. Rodamilans, B.; Valli, A.; Mingot, A.; San Leon, D.; Lopez-Moya, J.J.; Garcia, J.A. An atypical RNA silencing suppression strategy provides a snapshot of the evolution of sweet potato-infecting potyviruses. *Sci Rep* **2018**, *8*, 15937, doi:10.1038/s41598-018-34358-y.
385. Dickey, A.; Wang, N.; Cooper, E.; Tull, L.; Breedlove, D.; Mason, H.; Liu, D.; Wang, K.Y. Transient Expression of Lumbrokinase (PI239) in Tobacco (Nicotiana tabacum) Using a Geminivirus-Based Single Replicon System Dissolves Fibrin and Blood Clots. *Evid Based Complement Alternat Med* **2017**, *2017*, 6093017, doi:10.1155/2017/6093017.
386. Ferreira, M.J.; Vale, D.; Cunha, L.; Melo, P. Role of the C-terminal extension peptide of plastid located glutamine synthetase from Medicago truncatula: Crucial for enzyme activity and needless for protein import into the plastids. *Plant Physiol Biochem* **2017**, *111*, 226-233, doi:10.1016/j.plaphy.2016.11.015.
387. Vojta, L.; Ljuma-Skupnjak, L.; Budimir, A.; Vukicevic, S.; Fulgosi, H. Rapid transient expression of human granulocyte-macrophage colony-stimulating factor in two industrial cultivars of tobacco (Nicotiana tabacum L.) by agroinfiltration. *Biotechnol Rep (Amst)* **2015**, *7*, 81-86, doi:10.1016/j.btre.2015.05.006.

388. Jin, J.; Kim, M.J.; Dhandapani, S.; Tjhang, J.G.; Yin, J.L.; Wong, L.; Sarojam, R.; Chua, N.H.; Jang, I.C. The floral transcriptome of ylang ylang (Cananga odorata var. fruticosa) uncovers biosynthetic pathways for volatile organic compounds and a multifunctional and novel sesquiterpene synthase. *J Exp Bot* **2015**, *66*, 3959-3975, doi:10.1093/jxb/erv196.
389. Garvey, M.; Klinger, J.; Klose, H.; Fischer, R.; Commandeur, U. Expression of recombinant cellulase Cel5A from Trichoderma reesei in tobacco plants. *J Vis Exp* **2014**, 10.3791/51711, doi:10.3791/51711.
390. Zhang, Z.; Song, Y.; Liu, C.M.; Thomma, B.P. Mutational analysis of the Ve1 immune receptor that mediates Verticillium resistance in tomato. *PLoS One* **2014**, *9*, e99511, doi:10.1371/journal.pone.0099511.
391. Dittrich-Domergue, F.; Joubes, J.; Moreau, P.; Lessire, R.; Stymne, S.; Domergue, F. The bifunctional protein TtFARAT from Tetrahymena thermophila catalyzes the formation of both precursors required to initiate ether lipid biosynthesis. *J Biol Chem* **2014**, *289*, 21984-21994, doi:10.1074/jbc.M114.579318.
392. Buyel, J.F.; Kaever, T.; Buyel, J.J.; Fischer, R. Predictive models for the accumulation of a fluorescent marker protein in tobacco leaves according to the promoter/5'UTR combination. *Biotechnol Bioeng* **2013**, *110*, 471-482, doi:10.1002/bit.24715.
393. Huang, X.S.; Wang, W.; Zhang, Q.; Liu, J.H. A basic helix-loop-helix transcription factor, PtrbHLH, of Poncirus trifoliata confers cold tolerance and modulates peroxidase-mediated scavenging of hydrogen peroxide. *Plant Physiol* **2013**, *162*, 1178-1194, doi:10.1104/pp.112.210740.
394. Savada, R.P.; Bonham-Smith, P.C. Charge versus sequence for nuclear/nucleolar localization of plant ribosomal proteins. *Plant Mol Biol* **2013**, *81*, 477-493, doi:10.1007/s11103-013-0017-4.
395. Novakova, M.; Mackova, M.; Chrastilova, Z.; Viktorova, J.; Szekeres, M.; Demnerova, K.; Macek, T. Cloning the bacterial bphC gene into Nicotiana tabacum to improve the efficiency of PCB phytoremediation. *Biotechnol Bioeng* **2009**, *102*, 29-37, doi:10.1002/bit.22038.
396. Yanez, M.; Caceres, S.; Orellana, S.; Bastias, A.; Verdugo, I.; Ruiz-Lara, S.; Casaretto, J.A. An abiotic stress-responsive bZIP transcription factor from wild and cultivated tomatoes regulates stress-related genes. *Plant Cell Rep* **2009**, *28*, 1497-1507, doi:10.1007/s00299-009-0749-4.
397. Maimbo, M.; Ohnishi, K.; Hikichi, Y.; Yoshioka, H.; Kiba, A. Induction of a small heat shock protein and its functional roles in Nicotiana plants in the defense response against Ralstonia solanacearum. *Plant Physiol* **2007**, *145*, 1588-1599, doi:10.1104/pp.107.105353.
398. Zhao, M.M.; An, D.R.; Zhao, J.; Huang, G.H.; He, Z.H.; Chen, J.Y. Transiently expressed short hairpin RNA targeting 126 kDa protein of tobacco mosaic virus interferes with virus infection. *Acta Biochim Biophys Sin (Shanghai)* **2006**, *38*, 22-28, doi:10.1111/j.1745-7270.2006.00124.x.
399. Tarui, Y.; Iida, H.; Ono, E.; Miki, W.; Hirasawa, E.; Fujita, K.; Tanaka, T.; Taniguchi, M. Biosynthesis of poly-gamma-glutamic acid in plants: transient expression of poly-gamma-glutamate synthetase complex in tobacco leaves. *J Biosci Bioeng* **2005**, *100*, 443-448, doi:10.1263/jbb.100.443.
400. Liu, H.; Zhou, X.; Dong, N.; Liu, X.; Zhang, H.; Zhang, Z. Expression of a wheat MYB gene in transgenic tobacco enhances resistance to Ralstonia solanacearum, and to drought and salt stresses. *Funct Integr Genomics* **2011**, *11*, 431-443, doi:10.1007/s10142-011-0228-1.
401. Badel, J.L.; Shimizu, R.; Oh, H.S.; Collmer, A. A Pseudomonas syringae pv. tomato avrE1/hopM1 mutant is severely reduced in growth and lesion formation in tomato. *Mol Plant Microbe Interact* **2006**, *19*, 99-111, doi:10.1094/MPMI-19-0099.
402. Bendahmane, A.; Querci, M.; Kanyuka, K.; Baulcombe, D.C. Agrobacterium transient expression system as a tool for the isolation of disease resistance genes: application to the Rx2 locus in potato. *Plant J* **2000**, *21*, 73-81, doi:10.1046/j.1365-313x.2000.00654.x.
403. Lou, Y.; Ma, H.; Lin, W.H.; Chu, Z.Q.; Mueller-Roeber, B.; Xu, Z.H.; Xue, H.W. The highly charged region of plant beta-type phosphatidylinositol 4-kinase is involved in membrane targeting and phospholipid binding. *Plant Mol Biol* **2006**, *60*, 729-746, doi:10.1007/s11103-005-5548-x.
404. Tenea, G.N.; Spantzel, J.; Lee, L.Y.; Zhu, Y.; Lin, K.; Johnson, S.J.; Gelvin, S.B. Overexpression of several Arabidopsis histone genes increases agrobacterium-mediated transformation and transgene expression in plants. *Plant Cell* **2009**, *21*, 3350-3367, doi:10.1105/tpc.109.070607.

405. Laquitaine, L.; Gomes, E.; Francois, J.; Marchive, C.; Pascal, S.; Hamdi, S.; Atanassova, R.; Delrot, S.; Coutos-Thevenot, P. Molecular basis of ergosterol-induced protection of grape against botrytis cinerea: induction of type I LTP promoter activity, WRKY, and stilbene synthase gene expression. *Mol Plant Microbe Interact* **2006**, *19*, 1103-1112, doi:10.1094/MPMI-19-1103.
406. Cabral, A.; Oome, S.; Sander, N.; Kufner, I.; Nurnberger, T.; Van den Ackerveken, G. Nontoxic Nep1-like proteins of the downy mildew pathogen Hyaloperonospora arabidopsidis: repression of necrosis-inducing activity by a surface-exposed region. *Mol Plant Microbe Interact* **2012**, *25*, 697-708, doi:10.1094/MPMI-10-11-0269.
407. Jayaramaiah, R.H.; Anand, A.; Beedkar, S.D.; Dholakia, B.B.; Punekar, S.A.; Kalunke, R.M.; Gade, W.N.; Thulasiram, H.V.; Giri, A.P. Functional characterization and transient expression manipulation of a new sesquiterpene synthase involved in beta-caryophyllene accumulation in Ocimum. *Biochem Biophys Res Commun* **2016**, *473*, 265-271, doi:10.1016/j.bbrc.2016.03.090.
408. Ramesh, S.; Nagadhara, D.; Reddy, V.D.; Rao, K.V. Production of transgenic indica rice resistant to yellow stem borer and sap-sucking insects, using super-binary vectors of Agrobacterium tumefaciens. *Plant Science* **2004**, *166*, 1077-1085, doi:https://doi.org/10.1016/j.plantsci.2003.12.028.
409. Hosseini, B.; Shahriari-Ahmadi, F.; Hashemi, H.; Marashi, M.H.; Mohseniazar, M.; Farokhzad, A.; Sabokbari, M. Transient Expression of cor Gene in Papaver somniferum. *Bioimpacts* **2011**, *1*, 229-235, doi:10.5681/bi.2011.033.
410. Wang, X.; Zeng, W.; Ding, Y.; Wang, Y.; Niu, L.; Yao, J.L.; Pan, L.; Lu, Z.; Cui, G.; Li, G., et al. PpERF3 positively regulates ABA biosynthesis by activating PpNCED2/3 transcription during fruit ripening in peach. *Hortic Res* **2019**, *6*, 19, doi:10.1038/s41438-018-0094-2.
411. Zaragoza, C.; Munoz-Bertomeu, J.; Arrillaga, I. Regeneration of herbicide-tolerant black locust transgenic plants by SAAT. *Plant Cell Rep* **2004**, *22*, 832-838, doi:10.1007/s00299-004-0766-2.
412. Govind, K.; Makinen, K.; Savithri, H.S. Sesbania mosaic virus (SeMV) infectious clone: possible mechanism of 3' and 5' end repair and role of polyprotein processing in viral replication. *PLoS One* **2012**, *7*, e31190, doi:10.1371/journal.pone.0031190.
413. Fu, L.; Zhu, C.; Ding, X.; Yang, X.; Morris, P.F.; Tyler, B.M.; Zhang, X. Characterization of Cell-Death-Inducing Members of the Pectate Lyase Gene Family in Phytophthora capsici and Their Contributions to Infection of Pepper. *Mol Plant Microbe Interact* **2015**, *28*, 766-775, doi:10.1094/MPMI-11-14-0352-R.
414. Adkar-Purushothama, C.R.; Perreault, J.P. Alterations of the viroid regions that interact with the host defense genes attenuate viroid infection in host plant. *RNA Biol* **2018**, *15*, 955-966, doi:10.1080/15476286.2018.1462653.
415. Liu, X.; Dong, X.; Liu, Z.; Shi, Z.; Jiang, Y.; Qi, M.; Xu, T.; Li, T. Repression of ARF10 by microRNA160 plays an important role in the mediation of leaf water loss. *Plant Mol Biol* **2016**, *92*, 313-336, doi:10.1007/s11103-016-0514-3.
416. Bakshi, S.; Sadhukhan, A.; Mishra, S.; Sahoo, L. Improved Agrobacterium-mediated transformation of cowpea via sonication and vacuum infiltration. *Plant Cell Rep* **2011**, *30*, 2281-2292, doi:10.1007/s00299-011-1133-8.
417. He, Z.; Li, Z.; Lu, H.; Huo, L.; Wang, Z.; Wang, Y.; Ji, X. The NAC Protein from Tamarix hispida, ThNAC7, Confers Salt and Osmotic Stress Tolerance by Increasing Reactive Oxygen Species Scavenging Capability. *Plants (Basel)* **2019**, *8*, doi:10.3390/plants8070221.
418. Zhang, T.; Zhao, Y.; Wang, Y.; Liu, Z.; Gao, C. Comprehensive Analysis of MYB Gene Family and Their Expressions Under Abiotic Stresses and Hormone Treatments in Tamarix hispida. *Front Plant Sci* **2018**, *9*, 1303, doi:10.3389/fpls.2018.01303.
419. Yang, G.; Wang, C.; Wang, Y.; Guo, Y.; Zhao, Y.; Yang, C.; Gao, C. Overexpression of ThVHAc1 and its potential upstream regulator, ThWRKY7, improved plant tolerance of Cadmium stress. *Sci Rep* **2016**, *6*, 18752, doi:10.1038/srep18752.
420. Fister, A.S.; Landherr, L.; Maximova, S.N.; Guiltinan, M.J. Transient Expression of CRISPR/Cas9 Machinery Targeting TcNPR3 Enhances Defense Response in Theobroma cacao. *Front Plant Sci* **2018**, *9*, 268, doi:10.3389/fpls.2018.00268.
421. Helliwell, E.E.; Vega-Arreguin, J.; Shi, Z.; Bailey, B.; Xiao, S.; Maximova, S.N.; Tyler, B.M.; Guiltinan, M.J. Enhanced resistance in Theobroma cacao against oomycete and fungal

pathogens by secretion of phosphatidylinositol-3-phosphate-binding proteins. *Plant Biotechnol J* **2016**, *14*, 875-886, doi:10.1111/pbi.12436.
422. Viktorova, J.; Jandova, Z.; Madlenakova, M.; Prouzova, P.; Bartunek, V.; Vrchotova, B.; Lovecka, P.; Musilova, L.; Macek, T. Native Phytoremediation Potential of Urtica dioica for Removal of PCBs and Heavy Metals Can Be Improved by Genetic Manipulations Using Constitutive CaMV 35S Promoter. *PLoS One* **2016**, *11*, e0167927, doi:10.1371/journal.pone.0167927.
423. Guan, X.; Zhao, H.; Xu, Y.; Wang, Y. Transient expression of glyoxal oxidase from the Chinese wild grape Vitis pseudoreticulata can suppress powdery mildew in a susceptible genotype. *Protoplasma* **2011**, *248*, 415-423, doi:10.1007/s00709-010-0162-4.
424. Zhao, H.; Guan, X.; Xu, Y.; Wang, Y. Characterization of novel gene expression related to glyoxal oxidase by agro-infiltration of the leaves of accession Baihe-35-1 of Vitis pseudoreticulata involved in production of H2O2 for resistance to Erysiphe necator. *Protoplasma* **2013**, *250*, 765-777, doi:10.1007/s00709-012-0462-y.
425. Yu, Y.; Guo, D.; Li, G.; Yang, Y.; Zhang, G.; Li, S.; Liang, Z. The grapevine R2R3-type MYB transcription factor VdMYB1 positively regulates defense responses by activating the stilbene synthase gene 2 (VdSTS2). *BMC Plant Biol* **2019**, *19*, 478, doi:10.1186/s12870-019-1993-6.
426. Li, Z.T.; Dhekney, S.A.; Gray, D.J. Use of the VvMybA1 gene for non-destructive quantification of promoter activity via color histogram analysis in grapevine (Vitis vinifera) and tobacco. *Transgenic Res* **2011**, *20*, 1087-1097, doi:10.1007/s11248-010-9482-6.
427. Bertazzon, N.; Raiola, A.; Castiglioni, C.; Gardiman, M.; Angelini, E.; Borgo, M.; Ferrari, S. Transient silencing of the grapevine gene VvPGIP1 by agroinfiltration with a construct for RNA interference. *Plant Cell Rep* **2012**, *31*, 133-143, doi:10.1007/s00299-011-1147-2.
428. Yu, Y.; Jiao, L.; Fu, S.; Yin, L.; Zhang, Y.; Lu, J. Callose Synthase Family Genes Involved in the Grapevine Defense Response to Downy Mildew Disease. *Phytopathology* **2016**, *106*, 56-64, doi:10.1094/PHYTO-07-15-0166-R.
429. Xu, T.F.; Zhao, X.C.; Jiao, Y.T.; Wei, J.Y.; Wang, L.; Xu, Y. A pathogenesis related protein, VpPR-10.1, from Vitis pseudoreticulata: an insight of its mode of antifungal activity. *PLoS One* **2014**, *9*, e95102, doi:10.1371/journal.pone.0095102.
