# Supplementary material for: Transient Gene Expression is an Effective Experimental Tool for the Research into the Fine Mechanisms of Plant Gene Function: Advantages, Limitations, and Solutions
Source: Plants (Basel). 2020 Sep 11;9(9):1187. doi: 10.3390/plants9091187 (PMC7569937; doi:10.3390/plants9091187)
Supplement: Supplementary file 1 [file plants-09-01187-s001.zip › Table 2S.pdf]

Table 2S. Research areas utilizing transient gene expression in plants

	Vector name	Plant species	Experiment goals	Vector Construct Schemes	Reference
**Study of transcription factors (TF): plant cell localization (LOC) and physiological role (FR)**					
	pYBA1132-TwTGA1-eGFP	*Catharanthus roseus*	TF TwTGA1 *Tripterygium wilfordii*: LOC and FR in the synthesis of secondary metabolites	PCaMV35S;TwTGA1-**GFP**	[1]
	pTRV2-FvTCP6/9/14	*Fragaria vesca*	TF FvTCP9: LOC and FR in the ripening of strawberries	PCaMV35S;TRVstainppk20RNA5';FvTCP9;Tnos	[2]
	pLV07	*Nicotiana benthamiana*	TFs HlbZip1 и HlbZip2: LOC	P35S; HlbZip1 or HlbZip2 ;T35s	[3]
	pSAK277	*Nicotiana benthamiana*	Tobacco TF MYB: LOC	P35s;NtMYB3;ocs3';Pnos;nptII;Tnos	[4]
	pB7WG2D	*Nicotiana benthamiana*	Strawberry TF RsMYB1: LOC and FR in activation of anthocyanin synthesis	P35s;RsMYB1;T35s; proID;**EGFP**-ER;T35s	[5]
	pHEX2	*Nicotiana benthamiana*	Peach TF PpMYB10.4: LOC and FR in modification of anthocyanin biosynthesis	P35S;PpMYB10.4;TOCS	[6]
	PEAQ-HT	*Nicotiana benthamiana*	Lychee TF HB2 : LOC	P35S;CPMV RNA2 5';LcHB2 ;CPMV RNA2 3';Tnos	[7]
	pBI121	*Nicotiana benthamiana*	TF NAC: LOC	P35s;NAC-**GFP**;Tnos	[8]
	PBI121	*Allium cepa*	Maize TF NAC: LOC	P35s;ZmNAC55;**GUS**;Tnos	[9]
	pCAMBIA1301	*Juglans regia*	Walnut TF WRKY: LOC	P35S;JrWRKY2 or JrWRKY7 -**GUS**;Tnos	[10]
	pCAMBIA1301	*Juglans regia*	Walnut TF GRAS: LOC	P35S;JrGRAS2;cat1(int);**GUS**;Tnos	[11]
	pCAMBIA1381Z	*Nicotiana benthamiana*	Nanking Chrysanthemum TF TCP4: LOC	CnTCP4;cat1(int);**GUS**;Tnos	[12]
	pCAMBIA1303	*Nicotiana benthamiana*	Castor oil TF WRI1: LOC	RcWRI1;P35S;**GUS**;mgfp5;Tnos	[13]
	pCAMBIA1391	*Nicotiana tabacum*	*Trifoliate pontiurus* TF HLH: LOC	PtrbHLH -**GUS**;Tnos	[14]
	pGreenII0800-LUC	*Nicotiana benthamiana*	Cassava TFs MeWRKY79 , MeHsf20 or MeASMT2: LOC	P35s;Rluc;CaMV poly(A) signal; TF ;**LUC**, CaMV poly(A) signal	[15]
	pGreenII 0800-LUC	*Nicotiana benthamiana*	Hyacinth TF: LOC	P35S;RluC;MaAN2;CaMV poly(A) signal	[16]
	pGreenII0800-LUC	*Nicotiana benthamiana*	Rice TF: LOC	P35s;Rluc;CaMV poly(A) signal; OP1 ;**LUC**, CaMV poly(A) signal	[17]
	pGreenII0800-LUC	*Nicotiana benthamiana*	Rice TF WRKY80: LOC	P35s;Rluc;CaMV poly(A) signal; OsWRKY80-**EGFP**; CaMV poly(A) signal	[18]
**Functional role (FR) of gene products through silencing**					
	Ptrv	*Nicotiana benthamiana, Capsicum annuum*	patatin-like phospholipase CaPLP1 *Capsicum annuum*	PCaMV35S(;TRVstainppk20RNA5';CaDC1;Tnos	[19]
	pBIN35S	*Nicotiana benthamiana, Capsicum annuum*	Cysteine/histidine-rich DC1 domain protein *Capsicum annuum,*	P35S; CaPLP1 -mgfp5;Tnos	[20]

	pHANNIBAL	*Vitis vinifera*	VvPGIP1 *Vitis vinifera*	P35S;PDKnt;VvPGIP1;Tocs	[21]
	PFGC-Egfp, TRV2	*Nicotiana benthamiana*	kinase SlMKK2	P35S;TRVstainppk20rna5';SlMKK2;Tnos	[22]
	pCAMBIA2300U	*Nicotiana benthamiana*	acetylcholinesterase and ecdysone	P35S; AChE or EcR ;CaMV poly(A) signal	[23]
**Effector Study (E): Subcellular Localization (LOC) and Virulent Function (VIR-F)**					
	pTRV1	*Nicotiana benthamiana*	E phytophthora: LOC	P2x35S;pACP1/pEXP2;Tnoc	[24]
	pEGAD	*Nicotiana benthamiana*	E : LOC and VIR-F	P35S; Misp12 ;TNOS	[25]
	ImpGWB405	*Nicotiana benthamiana*	E : LOC	P35s;Las5315mp-**EGFP**	[26]
	Pff19-GUS	*Nicotiana benthamiana*	E : LOC	P35S;Avr1d -**GUS**;CaMV poly(A) signal	[27]
	pBin61	*Nicotiana spp.*	E : LOC	P35s;Avr3a;T35s	[28]
	PEAQ-HT	*Nicotiana benthamiana*	E phytophthora: LOC	P35S;CPMV RNA2 5';cEDIII;CPMV RNA2 3';Tnos	[29]
	PEAQ-HT	*Nicotiana benthamiana*	E phytophthora: VIR-F	P35S;CPMV RNA2 5';Lc-CedS;CPMV RNA2 3';Tnos	[30]
	pB7WGF2.0	*Nicotiana benthamiana*	E phytophthora: LOC	P35s;TmCAT1-**EGFP**;T35S	[31]
	pB7WGF2	*Nicotiana benthamiana*	E RXLR: LOC	P35s;EGFP-RXLR ;T35s	[32]
	pB7WG2	*Nicotiana benthamiana*	E : LOC	P35s;PiRXLR;T35S	[33]
	pB7WGF2	*Nicotiana benthamiana*	E phytophthora: LOC	P35s;FP-PiRXLR-**EGFP**;T35s	[34]
	pB7WGC2	*Nicotiana benthamiana*	E parasite salad: LOC	P35s;BLR38-**ECFP**;T35s	[35]
	pB7WGF2	*Nicotiana benthamiana*	E phytophthora: LOC	P35s;AVR2-**EGFP**;T35s	[36]
	pB7WGF2	*Nicotiana benthamiana*	E phytophthora: LOC	P35s;PiX3R-**EGFP**; T35s	[37]
	pH7FWG2,0	*Nicotiana benthamiana*	E RXlr from *Plasmopara viticola*: LOC	P35s;PvRxLR55-**EGFP**;T35s	[38]
	pK7WG2D	*Nicotiana benthamiana*	E phytophthora: LOC	P35S; BaP1;T35S;P35s;**EGFP**;T35S	[39]
	pk7WG2, pk7FWG2	*Nicotiana benthamiana*	E parasitic nematode: LOC and VIR-F	P35s; Mg16820;T35s	[40]
	PK7FWG2.0	*Nicotiana benthamiana*	E phytophthora	P35s;RXLR-**EGFP**	[41]
	pK7FWG2	*Nicotiana benthamiana*	E : LOC	P35s;XopQ-**EGFP**;T35S	[42]
	pK7WGF2	*Nicotiana benthamiana*	E parasitic nematode: LOC	P35s;Gp SPRY-414-2-**EGFP**;T35s	[43]
	pICH86988	*Nicotiana benthamiana*	E parasite fungus: LOC and VIR-F	P35S;HpSSP35.8;TOCS	[44]
	pICH86988	*Nicotiana benthamiana*	E : LOC and VIR-F	P35S;III Psa;TOCS	[45]
	pICH86988	*Nicotiana benthamiana*	E coniferous parasite fungus: VIR-F	P35S;HaSSPs;TOCS	[46]
	pGR107	*Nicotiana benthamiana*	E phytophthora: VIR-F	P35S; SCRE2	[47]
	pGR107	*Nicotiana benthamiana*	E phytophthora: LOC	P35S; Avh238	[48]
	pGR107	*Nicotiana benthamiana*	E Msp40: VIR-F	P35S;MSP4	[49]
	pBI121	*Nicotiana benthamiana*	E phytophthora: LOC	P35s;CsF3'H-**GUS**;Tnos	[50]
	pBI121	*Nicotiana benthamiana*	E phytophthora: LOC	P35s;ScDREB10-**GUS**;Tnos	[51]
	pBI121,	*Nicotiana benthamiana*	E phytophthora: LOC	P35s;Avr4/6-**GUS**;Tnos	[52]

	pCAMBIA1300				
	PCAMBIA1305.1	*Nicotiana benthamiana*	E phytophthora: LOC	P35S;**GUSplus**^MSP18;Tnoc	[53]
	pCAMBIA2300	*Nicotiana benthamiana*	E phytophthora: LOC and VIR-F	P35S; MbA;CaMV poly(A) signal	[54]
	PCAMBIA1305.1	*Nicotiana benthamiana*	E phytophthora: LOC	P35S;**GUSplus**;TNFR2;noc(term)	[55]
	pCAMBIA1301	*Nicotiana benthamiana*	E phytophthora: LOC	P35S;WRINKLED1-**GUS**;Tnos	[56]
	pCAMBIA1302	*Nicotiana benthamiana*	E parasitic nematode: LOC	P35S;MiISE5 ;mgfp5;Tnos	[57]
	PCAMBIA-CR1	*Nicotiana benthamiana, Helianthus annuus*	E oomycete, sunflower parasite: LOC	P35s;RXLR or CRN-**GFP** ;T35S	[58]
	pCambia1305	*Nicotiana benthamiana*	E MeTCTP из *Meloidogyne enterolobii*: LOC	P35S;MeTCTP-**GUSplus**;Tnos	[59]
	pCAMBIA1305	*Nicotiana benthamiana*	E : LOC and VIR-F	TALE;GUSplus;Tnos	[60]
	pGWB	*Nicotiana benthamiana*	E HopQ1: LOC	HopQ1/HopQ1-S51A ;Tnos	[61]
	pGWB5	*Citrus paradisi*	E AvrGf1 типа III из *Xanthomonas citri subsp*.: LOC	P35S; AvrGf1-**EGFP**;Tnos	[62]
	pGWB5	*Nicotiana benthamiana*	E *Xanthomonas* типа III AvrBs1, AvrBs3 и AvrBs4: VIR-F	P35S;AvrBs1/ AvrBs3/AvrBs4-EGFP;Tnos	[63]
**The study of the interaction type: plant-pathogen, plant-virus**					
	pGR107	*Nicotiana benthamiana*	FR SCRE2 gene from the parasite *Ustilaginoidea virens* in the development of infection in rice	P35S; SCRE2	[64]
	pgR107	*Nicotiana benthamiana*	The effect of temperature on the interaction of plants and pathogens	P35S;GFP	[65]
	pBI121	*Nicotiana benthamiana*	FR NbAGO2 tobacco gene in the development of viral infection	P35s;NbAGO2 ;**GUS**; Tnos; nptII	[66]
	pDONOR207	*Nicotiana benthamiana*	The interaction of the virus and plants	PPc;GmR;PLRV	[67]
	pTRV2	*Nicotiana benthamiana*	The study of Potato spindle tuber viroid	PCaMV35S;TRVstain ppk20 RNA5';PSTVd;Tnos	[68]
	PEarleyGate202	*Nicotiana benthamiana*	The study of viral RNA translation	P35S;TMVΩ;FLAG;DRP1;TOCS;PMAS;BlpR ;TMAS	[69]
	pCAMBIA2300	*Nicotiana benthamiana*	FR PvAvh74 gene in the formation of the immune response of tobacco	P35S; PvAvh74;CaMV poly(A) signal	[70]
**Protein screening, viral particle assembly, protein-protein interaction**					
	PEAQ-HT	*Nicotiana benthamiana*	Assembly of nano-sticks from viral proteins	P35S;CPMV RNA2 5';CP9,CPHis or OAS;CPMV RNA2 3';Tnos	[71]
	PEAQ-HT	*Nicotiana benthamiana*	Viral particle assembly	P35S;CPMV RNA2 5'; VLP BYDV, PLRV or PEMV ;CPMV RNA2 3';Tnos	[72]
	PCAMBIA 2300	*Nicotiana tabacum*	FR sugarcane phosphatidylinositol transporter	P35S; SlPAO1 , SlRboh3 or SlRboh4S;CaMV poly(A) signal	[73]

	pCambia3300u	*Nicotiana benthamiana*	Testing Renilla luciferase protein to study protein-protein interactions	P35S;GT;CaMV poly(A) signal	[74]
	pCAMBIA1302	*Nicotiana benthamiana*	FR calcineurin-B-like protein and its protein kinase interacting	P35S;MeCIPK23 or MeCBL1/9;mgfp5;Tnoc	[75]
	pGGB002	*Nicotiana benthamiana*	Screening for Proteins Involved in Unfold Protein Formation	PSp6;TMVΩ	[76]
	pBinAR	*Nicotiana benthamiana, Arabidopsis thaliana*	Development of a tandem non-invasive timer based on fluorescent proteins with different ripening times	P35S;SAT5-tFT;TOCS	[77]
	pBIN61	*Nicotiana benthamiana*	Assessment of the effect of microRNAs of different lengths (20 and 22 nucleotides) on the level of small interfering RNAs (siRNAs)	P35S;MIR173/MIR319/MIR828/MIR771-GFP;T35S	[78]
	pMDC32	*Nicotiana benthamiana*	Testing various fluorescent proteins in plants	P2X35S;FP;Tnos	[79]
	pMDC43	*Nicotiana tabacum*	Zinc and cadmium tobacco transporter studies	P2X35S;NtZIP4B-EGFP;Tnos	[80]
**Development of vectors and efficient transient expression (TE) techniques**					
	pSITE	*Nicotiana benthamiana*	PSITE Vector Family for Gateway Cloning	P2x35s;EGFP	[81]
	PSMV-Dual	*Nicotiana benthamiana*	New vector for TE	P1;PHC;p3;Cl;PNia;nib;CP	[82]
	pBGWL7	*Rosa chinensi*	TE for *Rosa chinensi*	RoKSN-**LUC**;T35s	[83]
	PEAQ-HT	*Nicotiana benthamiana*	Vectors for overexpression and subcellular localization	P35S;CPMV RNA2 5'; TagBFP;CPMV RNA2 3';Tnos	[84]
	pGreen 0029	*Vitis vinifera*	Vectors for assessing the localization of target proteins	**GFP/YFP/mRFP1**;Pnos;NeoR/KanR;Tnos	[85]
	pCAMBIA1301	*Narcissus tazzeta*	TE for *Narcissus tazzeta var chinensis*	P35S;anti-psy1-**GUS**;Tnos	[86]
	pCAMBIA1390, pBISN1	*Arabidopsis thaliana*	TE for Arabidopsis seedlings	**GUS**	[87]
	PCAMBIA 1303	*Coffea arabica*	TE for Coffee Leaves	cry10Aa;P35S;**GUS**;mgfp5;Tnos	[88]
	pCAMBIA 0390	*Nicotiana benthamiana*	New vector for TE	PMMoV BR-DF01;Tnos	[89]
	PCAMBIA1305.2	*Caragana intermedia*	TE for *Caragana intermedia*	CiDREB1C ;cat1(int);**GUSplus**;Tnos	[90]
	pCAMBIA2301	*Solanum Lycopersicum*	TE for tomato	P35S;**GUS**-6XHis;Tnos	[91]
	pK7FWGF2	*Maesa lanceolata*	TE for *Maesa lanceolata*	P35S;NLS-**GFP**;T35S	[92]
	pK7FWG2	*S. bulbocastanum*	TE for potatoes	P35s;RAR1-**EGFP**;T35S	[93]
**Analysis of factors affecting the efficiency of Transient Expression (TE) and optimization of TE methods**					
	pBI121	*Solanum lycopersicum*	Agrobacterium culture density and TE efficacy	P35s;**GUS**;Tnos	[94]
	pIG121Hm	*Lilium x formolongi*	The effect of pH on TE Lilium x formolongi	P35S; **int-GUS**;Tnos	[95]
	plG121Hm	*Saintpaidla ionantha*	Agrobacterium pretreatment for TE for *Saintpaidla ionantha*	P35S; **GUS**;Tnos	[96]
	PEAQ-HT	*Nicotiana benthamiana*	Optimization of TE due to modification of 5' and 3'-UTR	P35S;CPMV RNA2 5';gen;CPMV RNA2 3';Tnos	[97]

	PEAQ-HT	*Nicotiana benthamiana*	TE optimization for Tobacco	P35S;CPMV RNA2 5';HspL;CPMV RNA2 3';Tnos	[98]
	PEAQ-HT-DEST1	*Pisum sativum*	TE optimization for peas	P35S;CPMV RNA2 5'; **GUS** ;CPMV RNA2 3';Tnos	[99]
	PEAQexpress-HT-GFP	*Nicotiana benthamiana*	Modulation of TE through the use of various 5`UTR	P35s;CPMVRNA5';*GFP*;CPMVRNA3';TNOS	[100]
	pBIN-mgfp5-ER	*Daucus carota*	Monitoring GFP expression in carrots	P35s;AsFAD2-**m-gfp5** ER;Tnos	[101]
	pTJK136	*Lens culinaris*	The study of the effectiveness of strains of *Agrobacterium tumefaciens* for TE	P35S; **int-GUS**;Tnos	[102]
	pBISN1	*Panicum virgatum*	Studying the effect of various surfactants on the effectiveness of TE millet with different strains of agrobacteria.	Pmas; **GUS**;Tnos	[103]
	pIG101Hm	*Brassica rapa*	Factors affecting TE of *Brassica rapa*	P35S;**GUS**;Tnos	[104]
	pTF102	*Luffa cylindrica*	TE optimization for Luffa	P35S;bar; IV2intron	[105]
	pGreen	*Tropaeolum majus*	TE optimization for nasturtium	P35S;MPK3: At3g45640 or MKK4: At1g51660	[106]
	pPZP212	*Nicotiana benthamiana*	TE optimization for Tobacco	miR168a;P35s;NeoR/KaNR;CaMV poly(A) signal	[107]
	pB7WG2D	*Nicotiana benthamiana*	TE optimization for Tobacco	P35s;**LUC**;T35s; proID;**EGFP** ER;T35s	[108]
	PCambia1305.1	*Glycine max*	TE optimization for soybeans	P35S;**GUSplus**;Tnos	[109]
	pCAMBIA2301	*Prunus salicina*	The effectiveness of TE for plums, depending on the strain of agrobacteria.	P35S;**GUS**-6XHis;Tnos	[110]
	pCambia1301	*Artemisia pallens*	TE optimization for sagebrush	P35S;SuidA-**GUS**;Tnos	[111]
	pCAMBIA1301	*Citrus sinensis*	Optimization of TE for epicotyl segments of sweet pineapple	P35S;cat1(int);**GUS**;Tnos	[102]
	PCAMBIA 1305.1	*Ricinus communis*	Agroinfiltration of castor seeds	P35S;LfKCS3-**GUSplus**;Tnoc	[112]

1. Han, J.; Liu, H.T.; Wang, S.C.; Wang, C.R.; Miao, G.P. A class I TGA transcription factor from *Tripterygium wilfordii* Hook.f. modulates the biosynthesis of secondary metabolites in both native and heterologous hosts. *Plant Sci* **2020**, *290*, 110293, doi:10.1016/j.plantsci.2019.110293.
2. Xie, Y.G.; Ma, Y.Y.; Bi, P.P.; Wei, W.; Liu, J.; Hu, Y.; Gou, Y.J.; Zhu, D.; Wen, Y.Q.; Feng, J.Y. Transcription factor FvTCP9 promotes strawberry fruit ripening by regulating the biosynthesis of abscisic acid and anthocyanins. *Plant Physiol Biochem* **2020**, *146*, 374-383, doi:10.1016/j.plaphy.2019.11.004.
3. Matousek, J.; Kocabek, T.; Patzak, J.; Stehlik, J.; Fussy, Z.; Krofta, K.; Heyerick, A.; Roldan-Ruiz, I.; Maloukh, L.; De Keukeleire, D. Cloning and molecular analysis of HlbZip1 and HlbZip2 transcription factors putatively involved in the regulation of the lupulin metabolome in hop (*Humulus lupulus* L.). *J Agric Food Chem* **2010**, *58*, 902-912, doi:10.1021/jf9043106.
4. Anwar, M.; Yu, W.; Yao, H.; Zhou, P.; Allan, A.C.; Zeng, L. NtMYB3, an R2R3-MYB from Narcissus, Regulates Flavonoid Biosynthesis. *Int J Mol Sci* **2019**, *20*, doi:10.3390/ijms20215456.
5. Lim, S.H.; Song, J.H.; Kim, D.H.; Kim, J.K.; Lee, J.Y.; Kim, Y.M.; Ha, S.H. Activation of anthocyanin biosynthesis by expression of the radish R2R3-MYB transcription factor gene RsMYB1. *Plant Cell Rep* **2016**, *35*, 641-653, doi:10.1007/s00299-015-1909-3.
6. Zhou, Y.; Zhou, H.; Lin-Wang, K.; Vimolmangkang, S.; Espley, R.V.; Wang, L.; Allan, A.C.; Han, Y. Transcriptome analysis and transient transformation suggest an ancient duplicated MYB transcription factor as a candidate gene for leaf red coloration in peach. *BMC Plant Biol* **2014**, *14*, 388, doi:10.1186/s12870-014-0388-y.
7. Li, C.; Zhao, M.; Ma, X.; Wen, Z.; Ying, P.; Peng, M.; Ning, X.; Xia, R.; Wu, H.; Li, J. The HD-Zip transcription factor LcHB2 regulates litchi fruit abscission through the activation of two cellulase genes. *J Exp Bot* **2019**, *70*, 5189-5203, doi:10.1093/jxb/erz276.
8. Pascual, M.B.; Canovas, F.M.; Avila, C. The NAC transcription factor family in maritime pine (Pinus Pinaster): molecular regulation of two genes involved in stress responses. *BMC Plant Biol* **2015**, *15*, 254, doi:10.1186/s12870-015-0640-0.
9. Mao, H.; Yu, L.; Han, R.; Li, Z.; Liu, H. ZmNAC55, a maize stress-responsive NAC transcription factor, confers drought resistance in transgenic Arabidopsis. *Plant Physiol Biochem* **2016**, *105*, 55-66, doi:10.1016/j.plaphy.2016.04.018.
10. Yang, G.; Zhang, W.; Liu, Z.; Yi-Maer, A.Y.; Zhai, M.; Xu, Z. Both JrWRKY2 and JrWRKY7 of Juglans regia mediate responses to abiotic stresses and abscisic acid through formation of homodimers and interaction. *Plant Biol (Stuttg)* **2017**, *19*, 268-278, doi:10.1111/plb.12524.
11. Yang, G.; Gao, X.; Ma, K.; Li, D.; Jia, C.; Zhai, M.; Xu, Z. The walnut transcription factor JrGRAS2 contributes to high temperature stress tolerance involving in Dof transcriptional regulation and HSP protein expression. *BMC Plant Biol* **2018**, *18*, 367, doi:10.1186/s12870-018-1568-y.
12. Qi, X.; Qu, Y.; Gao, R.; Jiang, J.; Fang, W.; Guan, Z.; Zhang, F.; Zhao, S.; Chen, S.; Chen, F., et al. The Heterologous Expression of a Chrysanthemum nankingense TCP Transcription Factor Blocks Cell Division in Yeast and Arabidopsis thaliana. *Int J Mol Sci* **2019**, *20*, doi:10.3390/ijms20194848.
13. Ji, X.J.; Mao, X.; Hao, Q.T.; Liu, B.L.; Xue, J.A.; Li, R.Z. Splice Variants of the Castor WRI1 Gene Upregulate Fatty Acid and Oil Biosynthesis When Expressed in Tobacco Leaves. *Int J Mol Sci* **2018**, *19*, doi:10.3390/ijms19010146.
14. Huang, X.S.; Wang, W.; Zhang, Q.; Liu, J.H. A basic helix-loop-helix transcription factor, PtrbHLH, of Poncirus trifoliata confers cold tolerance and modulates peroxidase-mediated scavenging of hydrogen peroxide. *Plant Physiol* **2013**, *162*, 1178-1194, doi:10.1104/pp.112.210740.
15. Wei, Y.; Liu, G.; Bai, Y.; Xia, F.; He, C.; Shi, H.; Foyer, C. Two transcriptional activators of N-acetylserotonin O-methyltransferase 2 and melatonin biosynthesis in cassava. *J Exp Bot* **2017**, *68*, 4997-5006, doi:10.1093/jxb/erx305.
16. Chen, K.; Liu, H.; Lou, Q.; Liu, Y. Ectopic Expression of the Grape Hyacinth (*Muscari armeniacum)* R2R3-MYB Transcription Factor Gene, MaAN2, Induces Anthocyanin Accumulation in Tobacco. *Front Plant Sci* **2017**, *8*, 965, doi:10.3389/fpls.2017.00965.
17. Zhan, Y.; Sun, X.; Rong, G.; Hou, C.; Huang, Y.; Jiang, D.; Weng, X. Identification of two transcription factors activating the expression of OsXIP in rice defence response. *BMC Biotechnol* **2017**, *17*, 26, doi:10.1186/s12896-017-0344-7.
18. Peng, X.; Wang, H.; Jang, J.C.; Xiao, T.; He, H.; Jiang, D.; Tang, X. OsWRKY80-OsWRKY4 Module as a Positive Regulatory Circuit in Rice Resistance Against *Rhizoctonia solani*. *Rice (N Y)* **2016**, *9*, 63, doi:10.1186/s12284-016-0137-y.

19. Acosta-Maspons, A.; Sepulveda-Garcia, E.; Sanchez-Baldoquin, L.; Marrero-Gutierrez, J.; Pons, T.; Rocha-Sosa, M.; Gonzalez, L. Two aspartate residues at the putative p10 subunit of a type II metacaspase from *Nicotiana tabacum* L. may contribute to the substrate-binding pocket. *Planta* **2014**, *239*, 147-160, doi:10.1007/s00425-013-1975-0.
20. Kim, D.S.; Jeun, Y.; Hwang, B.K. The pepper patatin-like phospholipase CaPLP1 functions in plant cell death and defense signaling. *Plant Mol Biol* **2014**, *84*, 329-344, doi:10.1007/s11103-013-0137-x.
21. Bertazzon, N.; Raiola, A.; Castiglioni, C.; Gardiman, M.; Angelini, E.; Borgo, M.; Ferrari, S. Transient silencing of the grapevine gene VvPGIP1 by agroinfiltration with a construct for RNA interference. *Plant Cell Rep* **2012**, *31*, 133-143, doi:10.1007/s00299-011-1147-2.
22. Li, X.; Zhang, Y.; Huang, L.; Ouyang, Z.; Hong, Y.; Zhang, H.; Li, D.; Song, F. Tomato SlMKK2 and SlMKK4 contribute to disease resistance against *Botrytis cinerea*. *BMC Plant Biol* **2014**, *14*, 166, doi:10.1186/1471-2229-14-166.
23. Malik, H.J.; Raza, A.; Amin, I.; Scheffler, J.A.; Scheffler, B.E.; Brown, J.K.; Mansoor, S. RNAi-mediated mortality of the whitefly through transgenic expression of double-stranded RNA homologous to acetylcholinesterase and ecdysone receptor in tobacco plants. *Sci Rep* **2016**, *6*, 38469, doi:10.1038/srep38469.
24. Zhao, Y.; Mao, W.; Chen, Y.; Wang, W.; Dai, Z.; Dou, Z.; Zhang, K.; Wei, L.; Li, T.; Zeng, B., et al. Optimization and standardization of transient expression assays for gene functional analyses in strawberry fruits. *Hortic Res* **2019**, *6*, 53, doi:10.1038/s41438-019-0135-5.
25. Xie, J.; Li, S.; Mo, C.; Wang, G.; Xiao, X.; Xiao, Y. A Novel Meloidogyne incognita Effector Misp12 Suppresses Plant Defense Response at Latter Stages of Nematode Parasitism. *Front Plant Sci* **2016**, *7*, 964, doi:10.3389/fpls.2016.00964.
26. Pitino, M.; Armstrong, C.M.; Cano, L.M.; Duan, Y. Transient Expression of Candidatus Liberibacter Asiaticus Effector Induces Cell Death in *Nicotiana benthamiana*. *Front Plant Sci* **2016**, *7*, 982, doi:10.3389/fpls.2016.00982.
27. Yin, W.; Dong, S.; Zhai, L.; Lin, Y.; Zheng, X.; Wang, Y. The Phytophthora sojae Avr1d gene encodes an RxLR-dEER effector with presence and absence polymorphisms among pathogen strains. *Mol Plant Microbe Interact* **2013**, *26*, 958-968, doi:10.1094/MPMI-02-13-0035-R.
28. Vega-Arreguin, J.C.; Jalloh, A.; Bos, J.I.; Moffett, P. Recognition of an Avr3a homologue plays a major role in mediating nonhost resistance to *Phytophthora capsici* in Nicotiana species. *Mol Plant Microbe Interact* **2014**, *27*, 770-780, doi:10.1094/MPMI-01-14-0014-R.
29. Pang, E.L.; Peyret, H.; Ramirez, A.; Loh, H.S.; Lai, K.S.; Fang, C.M.; Rosenberg, W.M.; Lomonossoff, G.P. Epitope Presentation of Dengue Viral Envelope Glycoprotein Domain III on Hepatitis B Core Protein Virus-Like Particles Produced in *Nicotiana benthamiana*. *Front Plant Sci* **2019**, *10*, 455, doi:10.3389/fpls.2019.00455.
30. Luo, F.; Ling, Y.; Li, D.S.; Tang, T.; Liu, Y.C.; Liu, Y.; Li, S.H. Characterization of a sesquiterpene cyclase from the glandular trichomes of *Leucosceptrum canum* for sole production of cedrol in *Escherichia coli* and *Nicotiana benthamiana*. *Phytochemistry* **2019**, *162*, 121-128, doi:10.1016/j.phytochem.2019.03.009.
31. Tounsi, S.; Kamoun, Y.; Feki, K.; Jemli, S.; Saidi, M.N.; Ziadi, H.; Alcon, C.; Brini, F. Localization and expression analysis of a novel catalase from T*riticum monococcum* TmCAT1 involved in response to different environmental stresses. *Plant Physiol Biochem* **2019**, *139*, 366-378, doi:10.1016/j.plaphy.2019.03.039.
32. Hamel, L.P.; Sekine, K.T.; Wallon, T.; Sugiwaka, Y.; Kobayashi, K.; Moffett, P. The Chloroplastic Protein THF1 Interacts with the Coiled-Coil Domain of the Disease Resistance Protein N' and Regulates Light-Dependent Cell Death. *Plant Physiol* **2016**, *171*, 658-674, doi:10.1104/pp.16.00234.
33. Zheng, X.; McLellan, H.; Fraiture, M.; Liu, X.; Boevink, P.C.; Gilroy, E.M.; Chen, Y.; Kandel, K.; Sessa, G.; Birch, P.R., et al. Functionally redundant RXLR effectors from *Phytophthora infestans* act at different steps to suppress early flg22-triggered immunity. *PLoS Pathog* **2014**, *10*, e1004057, doi:10.1371/journal.ppat.1004057.
34. Wang, S.; McLellan, H.; Bukharova, T.; He, Q.; Murphy, F.; Shi, J.; Sun, S.; van Weymers, P.; Ren, Y.; Thilliez, G., et al. Phytophthora infestans RXLR effectors act in concert at diverse subcellular locations to enhance host colonization. *J Exp Bot* **2019**, *70*, 343-356, doi:10.1093/jxb/ery360.
35. Pelgrom, A.J.E.; Eikelhof, J.; Elberse, J.; Meisrimler, C.N.; Raedts, R.; Klein, J.; Van den Ackerveken, G. Recognition of lettuce downy mildew effector BLR38 in *Lactuca serriola* LS102 requires two unlinked loci. *Mol Plant Pathol* **2019**, *20*, 240-253, doi:10.1111/mpp.12751.
36. Turnbull, D.; Wang, H.; Breen, S.; Malec, M.; Naqvi, S.; Yang, L.; Welsh, L.; Hemsley, P.; Zhendong, T.; Brunner, F., et al. AVR2 Targets BSL Family Members, Which Act as Susceptibility Factors to Suppress Host Immunity. *Plant Physiol* **2019**, *180*, 571-581, doi:10.1104/pp.18.01143.

37. Murphy, F.; He, Q.; Armstrong, M.; Giuliani, L.M.; Boevink, P.C.; Zhang, W.; Tian, Z.; Birch, P.R.J.; Gilroy, E.M. The Potato MAP3K StVIK Is Required for the Phytophthora infestans RXLR Effector Pi17316 to Promote Disease. *Plant Physiol* **2018**, *177*, 398-410, doi:10.1104/pp.18.00028.
38. Xiang, J.; Li, X.; Wu, J.; Yin, L.; Zhang, Y.; Lu, J. Studying the Mechanism of *Plasmopara viticola* RxLR Effectors on Suppressing Plant Immunity. *Front Microbiol* **2016**, *7*, 709, doi:10.3389/fmicb.2016.00709.
39. Gomes, M.; Alvarez, M.A.; Quellis, L.R.; Becher, M.L.; Castro, J.M.A.; Gameiro, J.; Caporrino, M.C.; Moura-da-Silva, A.M.; de Oliveira Santos, M. Expression of an scFv antibody fragment in *Nicotiana benthamiana* and *in vitro* assessment of its neutralizing potential against the snake venom metalloproteinase BaP1 from Bothrops asper. *Toxicon* **2019**, *160*, 38-46, doi:10.1016/j.toxicon.2019.02.011.
40. Reis, R.S.; Litholdo, C.G., Jr.; Bally, J.; Roberts, T.H.; Waterhouse, P.M. A conditional silencing suppression system for transient expression. *Sci Rep* **2018**, *8*, 9426, doi:10.1038/s41598-018-27778-3.
41. Ren, Y.; Armstrong, M.; Qi, Y.; McLellan, H.; Zhong, C.; Du, B.; Birch, P.R.J.; Tian, Z. Phytophthora infestans RXLR Effectors Target Parallel Steps in an Immune Signal Transduction Pathway. *Plant Physiol* **2019**, *180*, 2227-2239, doi:10.1104/pp.18.00625.
42. Adlung, N.; Prochaska, H.; Thieme, S.; Banik, A.; Bluher, D.; John, P.; Nagel, O.; Schulze, S.; Gantner, J.; Delker, C., et al. Non-host Resistance Induced by the Xanthomonas Effector XopQ Is Widespread within the Genus Nicotiana and Functionally Depends on EDS1. *Front Plant Sci* **2016**, *7*, 1796, doi:10.3389/fpls.2016.01796.
43. Mei, Y.; Wright, K.M.; Haegeman, A.; Bauters, L.; Diaz-Granados, A.; Goverse, A.; Gheysen, G.; Jones, J.T.; Mantelin, S. The Globodera pallida SPRYSEC Effector GpSPRY-414-2 That Suppresses Plant Defenses Targets a Regulatory Component of the Dynamic Microtubule Network. *Front Plant Sci* **2018**, *9*, 1019, doi:10.3389/fpls.2018.01019.
44. Wen, Z.; Raffaello, T.; Zeng, Z.; Pavicic, M.; Asiegbu, F.O. Chlorophyll fluorescence imaging for monitoring effects of Heterobasidion parviporum small secreted protein induced cell death and in planta defense gene expression. *Fungal Genet Biol* **2019**, *126*, 37-49, doi:10.1016/j.fgb.2019.02.003.
45. Choi, S.; Jayaraman, J.; Segonzac, C.; Park, H.J.; Park, H.; Han, S.W.; Sohn, K.H. Pseudomonas syringae pv. actinidiae Type III Effectors Localized at Multiple Cellular Compartments Activate or Suppress Innate Immune Responses in Nicotiana benthamiana. *Front Plant Sci* **2017**, *8*, 2157, doi:10.3389/fpls.2017.02157.
46. Raffaello, T.; Asiegbu, F.O. Small secreted proteins from the necrotrophic conifer pathogen Heterobasidion annosum s.l. (HaSSPs) induce cell death in *Nicotiana benthamiana*. *Sci Rep* **2017**, *7*, 8000, doi:10.1038/s41598-017-08010-0.
47. Chen, X.R.; Huang, S.X.; Zhang, Y.; Sheng, G.L.; Zhang, B.Y.; Li, Q.Y.; Zhu, F.; Xu, J.Y. Transcription profiling and identification of infection-related genes in *Phytophthora cactorum*. *Mol Genet Genomics* **2018**, *293*, 541-555, doi:10.1007/s00438-017-1400-7.
48. Yang, B.; Wang, Q.; Jing, M.; Guo, B.; Wu, J.; Wang, H.; Wang, Y.; Lin, L.; Wang, Y.; Ye, W., et al. Distinct regions of the Phytophthora essential effector Avh238 determine its function in cell death activation and plant immunity suppression. *New Phytol* **2017**, *214*, 361-375, doi:10.1111/nph.14430.
49. Niu, J.; Liu, P.; Liu, Q.; Chen, C.; Guo, Q.; Yin, J.; Yang, G.; Jian, H. Msp40 effector of root-knot nematode manipulates plant immunity to facilitate parasitism. *Sci Rep* **2016**, *6*, 19443, doi:10.1038/srep19443.
50. Baba, S.A.; Ashraf, N. Functional characterization of flavonoid 3'-hydroxylase, CsF3'H, from *Crocus sativus* L: Insights into substrate specificity and role in abiotic stress. *Arch Biochem Biophys* **2019**, *667*, 70-78, doi:10.1016/j.abb.2019.04.012.
51. Li, X.; Liang, Y.; Gao, B.; Mijiti, M.; Bozorov, T.A.; Yang, H.; Zhang, D.; Wood, A.J. ScDREB10, an A-5c type of DREB Gene of the Desert Moss Syntrichia caninervis, Confers Osmotic and Salt Tolerances to Arabidopsis. *Genes (Basel)* **2019**, *10*, doi:10.3390/genes10020146.
52. Lan, X.; Liu, Y.; Song, S.; Yin, L.; Xiang, J.; Qu, J.; Lu, J. Plasmopara viticola effector PvRXLR131 suppresses plant immunity by targeting plant receptor-like kinase inhibitor BKI1. *Mol Plant Pathol* **2019**, *20*, 765-783, doi:10.1111/mpp.12790.
53. Grossi-de-Sa, M.; Petitot, A.S.; Xavier, D.A.; Sa, M.E.L.; Mezzalira, I.; Beneventi, M.A.; Martins, N.F.; Baimey, H.K.; Albuquerque, E.V.S.; Grossi-de-Sa, M.F., et al. Rice susceptibility to root-knot nematodes is enhanced by the Meloidogyne incognita MSP18 effector gene. *Planta* **2019**, *250*, 1215-1227, doi:10.1007/s00425-019-03205-3.
54. Irmisch, S.; Ruebsam, H.; Jancsik, S.; Man Saint Yuen, M.; Madilao, L.L.; Bohlmann, J. Flavonol Biosynthesis Genes and Their Use in Engineering the Plant Antidiabetic Metabolite Montbretin A. *Plant Physiol* **2019**, *180*, 1277-1290, doi:10.1104/pp.19.00254.

55. Bidarigh Fard, A.; Dehghan Nayeri, F.; Habibi Anbuhi, M. Transient expression of etanercept therapeutic protein in tobacco (*Nicotiana tabacum* L.). *Int J Biol Macromol* **2019**, *130*, 483-490, doi:10.1016/j.ijbiomac.2019.02.153.
56. Deng, S.; Mai, Y.; Shui, L.; Niu, J. WRINKLED1 transcription factor orchestrates the regulation of carbon partitioning for C18:1 (oleic acid) accumulation in Siberian apricot kernel. *Sci Rep* **2019**, *9*, 2693, doi:10.1038/s41598-019-39236-9.
57. Shi, Q.; Mao, Z.; Zhang, X.; Zhang, X.; Wang, Y.; Ling, J.; Lin, R.; Li, D.; Kang, X.; Sun, W., et al. A Meloidogyne incognita effector MiISE5 suppresses programmed cell death to promote parasitism in host plant. *Sci Rep* **2018**, *8*, 7256, doi:10.1038/s41598-018-24999-4.
58. Gascuel, Q.; Buendia, L.; Pecrix, Y.; Blanchet, N.; Munos, S.; Vear, F.; Godiard, L. RXLR and CRN Effectors from the Sunflower Downy Mildew Pathogen Plasmopara halstedii Induce Hypersensitive-Like Responses in Resistant Sunflower Lines. *Front Plant Sci* **2016**, *7*, 1887, doi:10.3389/fpls.2016.01887.
59. Zhuo, K.; Chen, J.; Lin, B.; Wang, J.; Sun, F.; Hu, L.; Liao, J. A novel Meloidogyne enterolobii effector MeTCTP promotes parasitism by suppressing programmed cell death in host plants. *Mol Plant Pathol* **2017**, *18*, 45-54, doi:10.1111/mpp.12374.
60. Zheng, C.K.; Wang, C.L.; Zhang, X.P.; Wang, F.J.; Qin, T.F.; Zhao, K.J. The last half-repeat of transcription activator-like effector (TALE) is dispensable and thereby TALE-based technology can be simplified. *Mol Plant Pathol* **2014**, *15*, 690-697, doi:10.1111/mpp.12125.
61. Giska, F.; Lichocka, M.; Piechocki, M.; Dadlez, M.; Schmelzer, E.; Hennig, J.; Krzymowska, M. Phosphorylation of HopQ1, a type III effector from Pseudomonas syringae, creates a binding site for host 14-3-3 proteins. *Plant Physiol* **2013**, *161*, 2049-2061, doi:10.1104/pp.112.209023.
62. Figueiredo, J.F.; Romer, P.; Lahaye, T.; Graham, J.H.; White, F.F.; Jones, J.B. Agrobacterium-mediated transient expression in citrus leaves: a rapid tool for gene expression and functional gene assay. *Plant Cell Rep* **2011**, *30*, 1339-1345, doi:10.1007/s00299-011-1045-7.
63. Gurlebeck, D.; Jahn, S.; Gurlebeck, N.; Szczesny, R.; Szurek, B.; Hahn, S.; Hause, G.; Bonas, U. Visualization of novel virulence activities of the Xanthomonas type III effectors AvrBs1, AvrBs3 and AvrBs4. *Mol Plant Pathol* **2009**, *10*, 175-188, doi:10.1111/j.1364-3703.2008.00519.x.
64. Fang, A.; Gao, H.; Zhang, N.; Zheng, X.; Qiu, S.; Li, Y.; Zhou, S.; Cui, F.; Sun, W. A Novel Effector Gene SCRE2 Contributes to Full Virulence of Ustilaginoidea virens to Rice. *Front Microbiol* **2019**, *10*, 845, doi:10.3389/fmicb.2019.00845.
65. Del Toro, F.; Tenllado, F.; Chung, B.N.; Canto, T. A procedure for the transient expression of genes by agroinfiltration above the permissive threshold to study temperature-sensitive processes in plant-pathogen interactions. *Mol Plant Pathol* **2014**, *15*, 848-857, doi:10.1111/mpp.12136.
66. Diao, P.; Zhang, Q.; Sun, H.; Ma, W.; Cao, A.; Yu, R.; Wang, J.; Niu, Y.; Wuriyanghan, H. miR403a and SA Are Involved in NbAGO2 Mediated Antiviral Defenses Against TMV Infection in *Nicotiana benthamiana*. *Genes (Basel)* **2019**, *10*, doi:10.3390/genes10070526.
67. Patton, M.F.; Bak, A.; Sayre, J.M.; Heck, M.L.; Casteel, C.L. A polerovirus, Potato leafroll virus, alters plant-vector interactions using three viral proteins. *Plant Cell Environ* **2020**, *43*, 387-399, doi:10.1111/pce.13684.
68. Adkar-Purushothama, C.R.; Iyer, P.S.; Perreault, J.P. Potato spindle tuber viroid infection triggers degradation of chloride channel protein CLC-b-like and Ribosomal protein S3a-like mRNAs in tomato plants. *Sci Rep* **2017**, *7*, 8341, doi:10.1038/s41598-017-08823-z.
69. Wu, G.; Cui, X.; Dai, Z.; He, R.; Li, Y.; Yu, K.; Bernards, M.; Chen, X.; Wang, A. A plant RNA virus hijacks endocytic proteins to establish its infection in plants. *Plant J* **2020**, *101*, 384-400, doi:10.1111/tpj.14549.
70. Yin, X.; Shang, B.; Dou, M.; Liu, R.; Chen, T.; Xiang, G.; Li, Y.; Liu, G.; Xu, Y. The Nuclear-Localized RxLR Effector PvAvh74 From Plasmopara viticola Induces Cell Death and Immunity Responses in *Nicotiana benthamiana*. *Front Microbiol* **2019**, *10*, 1531, doi:10.3389/fmicb.2019.01531.
71. Saunders, K.; Lomonossoff, G.P. In Planta Synthesis of Designer-Length Tobacco Mosaic Virus-Based Nano-Rods That Can Be Used to Fabricate Nano-Wires. *Front Plant Sci* **2017**, *8*, 1335, doi:10.3389/fpls.2017.01335.
72. Byrne, M.J.; Steele, J.F.C.; Hesketh, E.L.; Walden, M.; Thompson, R.F.; Lomonossoff, G.P.; Ranson, N.A. Combining Transient Expression and Cryo-EM to Obtain High-Resolution Structures of Luteovirid Particles. *Structure* **2019**, *27*, 1761-1770 e1763, doi:10.1016/j.str.2019.09.010.
73. Hua-Ying, M.; Wen-Ju, W.; Wei-Hua, S.; Ya-Chun, S.; Feng, L.; Cong-Na, L.; Ling, W.; Xu, Z.; Li-Ping, X.; You-Xiong, Q. Genome-wide identification, phylogeny, and expression analysis of Sec14-like PITP gene family in sugarcane. *Plant Cell Rep* **2019**, *38*, 637-655, doi:10.1007/s00299-019-02394-1.
74. Lund, C.H.; Bromley, J.R.; Stenbaek, A.; Rasmussen, R.E.; Scheller, H.V.; Sakuragi, Y. A reversible Renilla luciferase protein complementation assay for rapid identification of protein-protein interactions reveals

the existence of an interaction network involved in xyloglucan biosynthesis in the plant Golgi apparatus. *J Exp Bot* **2015**, *66*, 85-97, doi:10.1093/jxb/eru401.
75. Yan, Y.; He, X.; Hu, W.; Liu, G.; Wang, P.; He, C.; Shi, H. Functional analysis of MeCIPK23 and MeCBL1/9 in cassava defense response against *Xanthomonas axonopodis* pv. manihotis. *Plant Cell Rep* **2018**, *37*, 887-900, doi:10.1007/s00299-018-2276-7.
76. Alcantara, A.; Seitner, D.; Navarrete, F.; Djamei, A. A high-throughput screening method to identify proteins involved in unfolded protein response of the endoplasmic reticulum in plants. *Plant Methods* **2020**, *16*, 4, doi:10.1186/s13007-020-0552-3.
77. Zhang, H.; Linster, E.; Gannon, L.; Leemhuis, W.; Rundle, C.A.; Theodoulou, F.L.; Wirtz, M. Tandem Fluorescent Protein Timers for Noninvasive Relative Protein Lifetime Measurement in Plants. *Plant Physiol* **2019**, *180*, 718-731, doi:10.1104/pp.19.00051.
78. Chen, H.M.; Chen, L.T.; Patel, K.; Li, Y.H.; Baulcombe, D.C.; Wu, S.H. 22-Nucleotide RNAs trigger secondary siRNA biogenesis in plants. *Proc Natl Acad Sci U S A* **2010**, *107*, 15269-15274, doi:10.1073/pnas.1001738107.
79. Stoddard, A.; Rolland, V. I see the light! Fluorescent proteins suitable for cell wall/apoplast targeting in *Nicotiana benthamiana* leaves. *Plant Direct* **2019**, *3*, e00112, doi:10.1002/pld3.112.
80. Barabasz, A.; Palusinska, M.; Papierniak, A.; Kendziorek, M.; Kozak, K.; Williams, L.E.; Antosiewicz, D.M. Functional Analysis of NtZIP4B and Zn Status-Dependent Expression Pattern of Tobacco ZIP Genes. *Front Plant Sci* **2018**, *9*, 1984, doi:10.3389/fpls.2018.01984.
81. Chakrabarty, R.; Banerjee, R.; Chung, S.M.; Farman, M.; Citovsky, V.; Hogenhout, S.A.; Tzfira, T.; Goodin, M. PSITE vectors for stable integration or transient expression of autofluorescent protein fusions in plants: probing *Nicotiana benthamiana*-virus interactions. *Mol Plant Microbe Interact* **2007**, *20*, 740-750, doi:10.1094/MPMI-20-7-0740.
82. Seo, J.K.; Choi, H.S.; Kim, K.H. Engineering of soybean mosaic virus as a versatile tool for studying protein-protein interactions in soybean. *Sci Rep* **2016**, *6*, 22436, doi:10.1038/srep22436.
83. Lu, J.; Bai, M.; Ren, H.; Liu, J.; Wang, C. An efficient transient expression system for gene function analysis in rose. *Plant Methods* **2017**, *13*, 116, doi:10.1186/s13007-017-0268-1.
84. Berthold, F.; Roujol, D.; Hemmer, C.; Jamet, E.; Ritzenthaler, C.; Hoffmann, L.; Schmitt-Keichinger, C. Inside or outside? A new collection of Gateway vectors allowing plant protein subcellular localization or over-expression. *Plasmid* **2019**, *105*, 102436, doi:10.1016/j.plasmid.2019.102436.
85. Zottini, M.; Barizza, E.; Costa, A.; Formentin, E.; Ruberti, C.; Carimi, F.; Lo Schiavo, F. Agroinfiltration of grapevine leaves for fast transient assays of gene expression and for long-term production of stable transformed cells. *Plant Cell Rep* **2008**, *27*, 845-853, doi:10.1007/s00299-008-0510-4.
86. Lu, G.; Zou, Q.; Guo, D.; Zhuang, X.; Yu, X.; Xiang, X.; Cao, J. Agrobacterium tumefaciens-mediated transformation of *Narcissus tazzeta* var. chinensis. *Plant Cell Rep* **2007**, *26*, 1585-1593, doi:10.1007/s00299-007-0382-z.
87. Wu, H.Y.; Liu, K.H.; Wang, Y.C.; Wu, J.F.; Chiu, W.L.; Chen, C.Y.; Wu, S.H.; Sheen, J.; Lai, E.M. AGROBEST: an efficient Agrobacterium-mediated transient expression method for versatile gene function analyses in Arabidopsis seedlings. *Plant Methods* **2014**, *10*, 19, doi:10.1186/1746-4811-10-19.
88. Vargas-Guevara, C.; Vargas-Segura, C.; Villalta-Villalobos, J.; Pereira, L.F.P.; Gatica-Arias, A. A simple and efficient agroinfiltration method in coffee leaves (Coffea arabica L.): assessment of factors affecting transgene expression. *3 Biotech* **2018**, *8*, 471, doi:10.1007/s13205-018-1495-5.
89. Vasques, R.M.; Lacorte, C.; da Luz, L.L.; Aranda, M.A.; Nagata, T. Development of a new tobamovirus-based viral vector for protein expression in plants. *Mol Biol Rep* **2019**, *46*, 97-103, doi:10.1007/s11033-018-4449-4.
90. Liu, K.; Yang, Q.; Yang, T.; Wu, Y.; Wang, G.; Yang, F.; Wang, R.; Lin, X.; Li, G. Development of Agrobacterium-mediated transient expression system in Caragana intermedia and characterization of CiDREB1C in stress response. *BMC Plant Biol* **2019**, *19*, 237, doi:10.1186/s12870-019-1800-4.
91. Cruz-Mendívil, A.; Rivera-López, J.; Germán-Báez, L.J.; López-Meyer, M.; Hernández-Verdugo, S.; López-Valenzuela, J.A.; Reyes-Moreno, C.; Valdez-Ortiz, A. A Simple and Efficient Protocol for Plant Regeneration and Genetic Transformation of Tomato cv. Micro-Tom from Leaf Explants. **2011**, *46*, 1655, doi:10.21273/hortsci.46.12.1655.
92. Faizal, A.; Geelen, D. Agroinfiltration of intact leaves as a method for the transient and stable transformation of saponin producing *Maesa lanceolata*. *Plant Cell Reports* **2012**, *31*, 1517-1526, doi:10.1007/s00299-012-1266-4.
93. Faizal, A.; Geelen, D. Agroinfiltration of intact leaves as a method for the transient and stable transformation of saponin producing *Maesa lanceolata*. *Plant Cell Rep* **2012**, *31*, 1517-1526, doi:10.1007/s00299-012-1266-4.

94. Gao, N.; Shen, W.; Cao, Y.; Su, Y.; Shi, W. Influence of bacterial density during preculture on Agrobacterium-mediated transformation of tomato. *Plant Cell, Tissue and Organ Culture (PCTOC)* **2009**, *98*, 321-330, doi:10.1007/s11240-009-9566-2.
95. Ogaki, M.; Furuichi, Y.; Kuroda, K.; Chin, D.P.; Ogawa, Y.; Mii, M. Importance of co-cultivation medium pH for successful Agrobacterium-mediated transformation of Lilium x formolongi. *Plant Cell Rep* **2008**, *27*, 699-705, doi:10.1007/s00299-007-0481-x.
96. Kushikawa, S.; Miyoshi, K.; Mii, M. Pre-Culture Treatment Enhances Transient GUS Gene Expression in Leaf Segment of *Saintpaulia ionantha* Wendl. after Inoculation with *Agrobacterium tumefaciens*. *Plant Biotechnology* **2002**, *19*, 149-152, doi:10.5511/plantbiotechnology.19.149.
97. Peyret, H.; Brown, J.K.M.; Lomonossoff, G.P. Improving plant transient expression through the rational design of synthetic 5' and 3' untranslated regions. *Plant Methods* **2019**, *15*, 108, doi:10.1186/s13007-019-0494-9.
98. Norkunas, K.; Harding, R.; Dale, J.; Dugdale, B. Improving agroinfiltration-based transient gene expression in *Nicotiana benthamiana*. *Plant Methods* **2018**, *14*, 71, doi:10.1186/s13007-018-0343-2.
99. Guy, E.; Boulain, H.; Aigu, Y.; Le Pennec, C.; Chawki, K.; Morliere, S.; Schadel, K.; Kunert, G.; Simon, J.C.; Sugio, A. Optimization of Agroinfiltration in *Pisum sativum* Provides a New Tool for Studying the Salivary Protein Functions in the Pea Aphid Complex. *Front Plant Sci* **2016**, *7*, 1171, doi:10.3389/fpls.2016.01171.
100. Meshcheriakova, Y.A.; Saxena, P.; Lomonossoff, G.P. Fine-tuning levels of heterologous gene expression in plants by orthogonal variation of the untranslated regions of a nonreplicating transient expression system. *Plant Biotechnol J* **2014**, *12*, 718-727, doi:10.1111/pbi.12175.
101. Baranski, R.; Klocke, E.; Ryschka, U. Monitoring the expression of green fluorescent protein in carrot. *Acta Physiologiae Plantarum* **2007**, *29*, 239-246, doi:10.1007/s11738-007-0030-9.
102. de Oliveira, M.L.; Febres, V.J.; Costa, M.G.; Moore, G.A.; Otoni, W.C. High-efficiency Agrobacterium-mediated transformation of citrus via sonication and vacuum infiltration. *Plant Cell Rep* **2009**, *28*, 387-395, doi:10.1007/s00299-008-0646-2.
103. Song, G.-q.; Walworth, A.; Hancock, J.F. Factors influencing Agrobacterium-mediated transformation of switchgrass cultivars. *Plant Cell, Tissue and Organ Culture (PCTOC)* **2012**, *108*, 445-453, doi:10.1007/s11240-011-0056-y.
104. Takasaki, T.; Hatakeyama, K.; Kunihiko; Ojima, W.; Toriyama, K.; Hinata, K.; . Factors Influencing Agrobacterium-mediated Transformation of *Brassica rapa* L.
105. Li, S.; Cong, Y.; Liu, Y.; Wang, T.; Shuai, Q.; Chen, N.; Gai, J.; Li, Y. Optimization of Agrobacterium-Mediated Transformation in Soybean. *Front Plant Sci* **2017**, *8*, 246, doi:10.3389/fpls.2017.00246.
106. Pitzschke, A. Tropaeolum tops tobacco - simple and efficient transgene expression in the order Brassicales. *PLoS One* **2013**, *8*, e73355, doi:10.1371/journal.pone.0073355.
107. Bhagwat, B.; Chi, M.; Su, L.; Tang, H.; Tang, G.; Xiang, Y. An in vivo transient expression system can be applied for rapid and effective selection of artificial microRNA constructs for plant stable genetic transformation. *J Genet Genomics* **2013**, *40*, 261-270, doi:10.1016/j.jgg.2013.03.012.
108. Bashandy, H.; Jalkanen, S.; Teeri, T.H. Within leaf variation is the largest source of variation in agroinfiltration of *Nicotiana benthamiana*. *Plant Methods* **2015**, *11*, 47, doi:10.1186/s13007-015-0091-5.
109. King, J.L.; Finer, J.J.; McHale, L.K. Development and optimization of agroinfiltration for soybean. *Plant Cell Rep* **2015**, *34*, 133-140, doi:10.1007/s00299-014-1694-4.
110. Zong, X.; Chen, Q.; Nagaty, M.A.; Kang, Y.; Lang, G.; Song, G.-q. Adventitious shoot regeneration and Agrobacterium tumefaciens-mediated transformation of leaf explants of sweet cherry (*Prunus avium* L.). *The Journal of Horticultural Science and Biotechnology* **2019**, *94*, 229-236, doi:10.1080/14620316.2018.1470908.
111. Alok, A.; Shukla, V.; Pala, Z.; Kumar, J.; Kudale, S.; Desai, N. In vitro regeneration and optimization of factors affecting Agrobacterium mediated transformation in Artemisia Pallens, an important medicinal plant. *Physiol Mol Biol Plants* **2016**, *22*, 261-269, doi:10.1007/s12298-016-0353-3.
112. Sanchez-Alvarez, A.; Ruiz-Lopez, N.; Moreno-Perez, A.J.; Martinez-Force, E.; Garces, R.; Salas, J.J. Agrobacterium-Mediated Transient Gene Expression in Developing Ricinus communis Seeds: A First Step in Making the Castor Oil Plant a Chemical Biofactory. *Front Plant Sci* **2019**, *10*, 1410, doi:10.3389/fpls.2019.01410.
